# Interaction Hierarchy and Polymorphic Structure–Property Dynamics in Luminescent Molecular Crystals

**DOI:** 10.1002/anie.8807652

**Published:** 2026-06-30

**Authors:** Mahiro Nakabayashi, Shotaro Hayashi

**Affiliations:** ^1^ School of Engineering Science Kochi University of Technology, Kami Kochi Japan; ^2^ FOREST Center, Research Institute Kochi University of Technology, Kami Kochi Japan

**Keywords:** crystal engineering, intermolecular interactions, polymorphs, solid‐state phase transition, stimulus responses

## Abstract

Solid‐state phase transitions provide a powerful platform for translating subtle molecular‐level interactions into macroscopic functional responses; however, rational design strategies that enable predictable control over such transitions remain limited. Herein, we report polymorphic structure–property switching behaviors associated with competing intermolecular interactions in luminescent molecular crystals. Cyano‐*β*‐substituted distyrylbenzene derivatives bearing bromo and methoxy side chains were designed to incorporate competing homotypic and heterotypic noncovalent interactions with distinct interaction characteristics. Single‐crystal analyses reveal that subtle differences in the hierarchical ordering of dispersion‐, dipole–dipole‐, and electrostatically dominated interactions give rise to polymorphic crystal structures with distinct molecular orientations and photoluminescence properties. Thermal and mechanical stimuli induce distinct phase transitions: an irreversible thermally induced single‐crystal‐to‐single‐crystal transition and a pseudo‐reversible mechanochemical pathway via an amorphous intermediate, both directly visualized as pronounced emission color changes. Kinetic and thermodynamic controls over polymorph formation are elucidated through a combination of structural analysis, photophysical measurements, and crystal framework calculations. This study suggests that multifunctional molecular side chains enable access to diverse interaction landscapes, allowing multiple structure–property switching pathways to be encoded within a single molecular framework. The presented framework provides a qualitative perspective for interpreting dynamic structural behaviors in molecular crystals.

## Graphical abstract

Competing homotypic and heterotypic intermolecular interactions give rise to polymorphic structure–property dynamics in luminescent molecular crystals. Thermal and mechanical stimuli induce distinct phase‐transition pathways accompanied by pronounced photoluminescence changes.

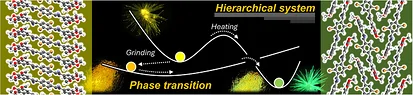

## 1 Introduction

Morphological changes at the molecular and supramolecular levels are fundamental phenomena governing the functions and responses of materials. These dynamics can be understood in terms of hierarchical relationships that extend across chemistry to physics, materials science, and the life sciences. Accordingly, a trans‐scale understanding spanning multiple structural hierarchies—from molecular architecture to the design of external physical fields—provides a useful perspective for analyzing such systems.

Precise control of solid‐state phase transitions has long been pursued as a means of creating new functionalities in molecular materials. Because such transitions involve reorganizations of atomic and molecular configurations, they can dramatically alter numerous material properties, including optical [1, 2, 3], electronic [4], magnetic [5], and mechanical [6] responses, as well as chemical structures [7] and even biological functions [8]. In all such cases, subtle molecular‐level structural transformations manifest as macroscale phenomena that are readily observable. However, only a limited number of molecular systems can accommodate the dynamic structural reconfigurations required for solid‐state sensing mechanisms. Azobenzene, diarylethene, and anthracene derivatives are well‐known examples, exhibiting diverse functions such as chromic and dynamic behavior owing to their ability to undergo light‐ and thermal‐driven structural rearrangements [9, 10, 11]. Yet few other molecular systems offer comparable functionality, resulting in a clear trade‐off between molecular design freedom and the complexity of achievable functions. To overcome this limitation, increasing attention has been directed toward strategies that employ the switching of intermolecular interactions as the driving force for solid‐state phase transitions [6, 12, 13, 14]. Because such transitions proceed according to thermodynamic principles governed by the enthalpy of noncovalent interactions, intermolecular forces—often regarded as complex—can be organized into a thermodynamic hierarchical framework. This hierarchy is particularly valuable as a descriptive and design‐oriented perspective for understanding and accessing dynamic structural behaviors in molecular crystals. Unlike conventional packing analysis that retrospectively rationalizes structures, the present interaction‐hierarchy‐based framework provides a qualitative perspective for interpreting competing intermolecular interactions associated with polymorphic behavior.

Two‐body intermolecular interactions can be broadly classified into homogeneous and heterogeneous types. Homogeneous interactions include hydrogen bonding between carboxylic acids, π–π interaction, dispersion‐dominated contacts between halogens, and dipole–dipole interactions between methoxy groups [15, 16, 17, 18]. The strengths of these interactions typically range from several to tens of kJ mol^−1^, enabling the construction of targeted supramolecular frameworks through appropriate selection of the interaction type [19]. Heterogeneous interactions, by contrast, include hydrogen bonding between carboxylic acids and pyridyl groups, CH–π interactions, and halogen bonding between halogens and heteroatoms [20, 21, 22]. Most heterogeneous interactions are predominantly governed by electrostatic forces arising from functional‐group polarization and therefore often exhibit stronger electrostatic contributions than dispersion‐ or dipole–dipole‐dominated interactions, depending on the structural context [23]. Several thermodynamically driven phase transitions based on both homogeneous and heterogeneous interactions have been reported. For example, the thermodynamic preference of CH–π interactions over π–π interactions has been exploited to induce single‐crystal‐to‐single‐crystal phase transitions upon thermal stimulation [24]. This behavior can be interpreted as a transition from a dispersion‐dominated molecular assembly to a more electrostatically stabilized framework in response to external stimulus. Accordingly, π–π and CH–π interactions can be regarded as distinct types of intermolecular interactions that contribute differently to molecular packing, depending on the specific structural context. The classification of these interactions serves as a qualitative framework to describe interaction patterns and does not necessarily correspond directly to interaction strength.

The design and spatial arrangement of intermolecular interactions play a crucial role in governing the mechanisms and conditions of phase transitions in molecular solids. A hierarchical understanding of electrostatic, dispersion, and dipole–dipole interactions provides a useful perspective for realizing molecular aggregates that undergo solid‐state phase transitions. Here, we report luminescent molecular aggregates exhibiting solid‐state phase transitions, achieved by multifunctionalizing the side chains of cyano‐*β*‐substituted distyrylbenzene (CDSB)—a scaffold with high conformational flexibility—with bromo and methoxy groups. Multifunctionality and molecular flexibility are essential prerequisites for realizing polymorphism and solid‐state phase transitions [25, 26, 27]. Whereas each homotypic interaction is classified as a relatively weak interaction, such as dispersion or dipole–dipole interactions, the bromo–methoxy halogen bond constitutes an interaction involving significant electrostatic contributions. Phase transitions arising from molecules that conform to this interaction hierarchy are captured as distinct changes in solid‐state photoluminescence (PL). This work highlights the usefulness of interaction hierarchy as a qualitative framework for understanding trans‐scale structure–property switching in molecular solids.

## 2 Results and Discussion

**FIGURE 1:**
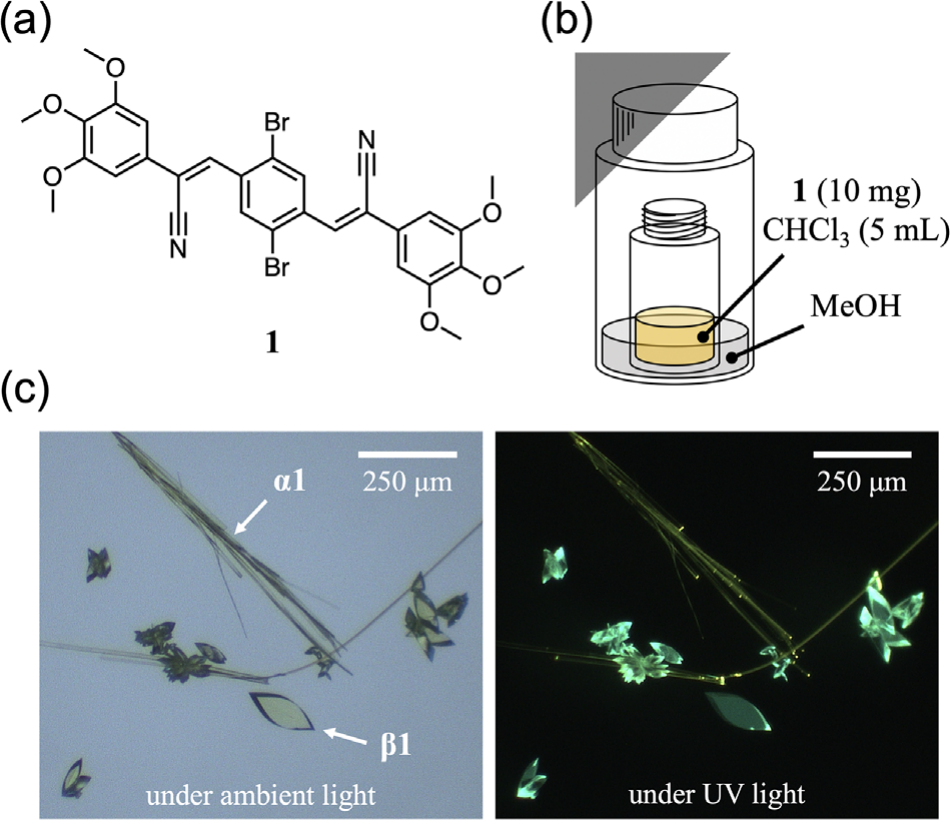
Compounds and crystals. (a) Chemical structures of **1**. (b) Illustration of crystallization conditions. (c) Optical microscope images of **α1** and **β1**.

The designed CDSB derivatives **1–4** possess bromine substituents along the molecular short axis and multiple methoxy groups along the molecular long axis (Figure 1a and Chart S1). Compounds **1–4** were synthesized via Knoevenagel condensation of 2,5‐dibromoterephthalaldehyde with methoxyphenylacetonitrile derivatives in methanol in the presence of a base (Schemes S1 and S2 and Figures S1–S12). Crystallization screening revealed that only compound **1** exhibited polymorphism and stimuli‐responsive phase‐transition behavior, whereas the other derivatives did not yield single crystals suitable for structural analysis despite extensive crystallization trials, preventing direct evaluation of their packing structures and phase‐transition behavior. Single crystals of **1** were obtained by a vapor diffusion method using chloroform as a good solvent and methanol as a poor solvent at room temperature in the dark. Optical microscopy of crystals **1** showed two distinct crystal morphologies, needle‐shaped crystals (**α1**) and block‐shaped crystals (**β1**) (Figure 1b). Under UV light irradiation, **α1** and **β1** exhibited yellow‐ and green‐emission, respectively (Figure 1c). Solid‐state PL spectra revealed that the maximum PL wavelengths of **α1** and **β1** were located at 550 and 525 nm (Figure S13). Additionally, the PL lifetimes of **α1** and **β1** were distinct and were markedly shorter than that of **1** dispersed in a polymer matrix, suggesting that the emission proceeds via aggregation‐specific photophysical pathways (Figures S14 and S15 and Table S1). To further elucidate this behavior, the radiative (*k*_r_) and nonradiative (*k*_nr_) rate constants were estimated from the experimentally determined photoluminescence quantum yields and lifetimes (Table S2). The *k*_nr_ values of **α1** and **β1** are comparable, whereas the *k*_r_ values differ by approximately a factor of 2.5, indicating that the variation in emission properties primarily originates from differences in the radiative decay process. Compared with the PMMA‐dispersed state, both *k*_r_ and *k*_nr_ are larger in the crystalline phases, suggesting that intermolecular electronic interactions in the aggregated state significantly modify both radiative and nonradiative decay pathways. Such behavior is consistent with previous studies on molecular aggregates and crystalline solids, where excitonic coupling and packing‐dependent interactions give rise to distinct emission dynamics compared to isolated molecules [28, 29, 30]. Therefore, the differences in crystal morphology and emission color between **α1** and **β1** indicate that these crystals are distinct polymorphs.

**FIGURE 2:**
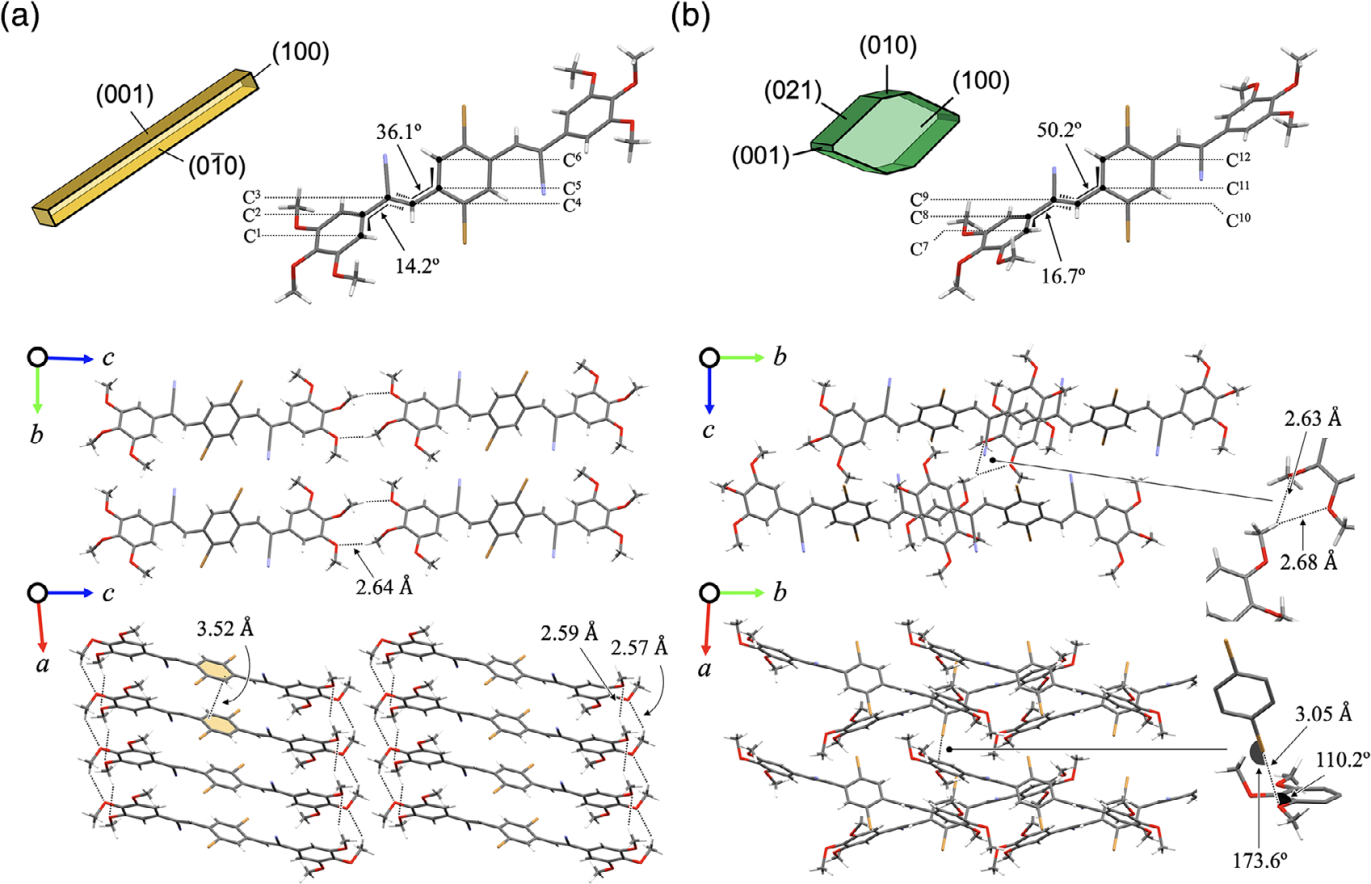
Compounds and crystal structures. (a, b) Single‐crystal structures of the **α1** and **β1** phases, respectively. Thermal ellipsoids are drawn at the 50% probability level.

Figure 2a shows the single‐crystal x‐ray structure of the **α1** polymorph (Table S3; CCDC dep. no.: 2523856, triclinic, *P*‒1). All crystal faces of **α1** were indexed as low‐index planes, and anisotropic crystal growth along the *a*‐axis was observed. The torsion angle between the acrylonitrile and trimethoxyphenylene moieties (C^1–^C^2–^C^3–^C^4^) is 14.2°, indicating a relatively high degree of planarity. In contrast, the torsion angle between the acrylonitrile and dibromophenylene moieties (C^3–^C^4–^C^5–^C^6^) is 36.1°. This twisted conformation is presumably caused by exchange repulsion between the nitrogen atom of the acrylonitrile and the hydrogen atoms on the dibromophenylene ring. Indeed, conformational analysis based on theoretical calculations suggests that a molecule **1** in **α1** adopts a geometry close to the most stable conformation (Figures S16 and S17). Focusing on the intermolecular interactions in **α1**, a molecule **1** forms methoxy–methoxy dipole–dipole interactions along the *a*‐ and *c*‐axes. For all methoxy–methoxy interactions, the OCH_2–_H···O–CH_3_ intermolecular distances fell within the range of 2.5–2.7 Å. Although all of these contacts correspond to the homogeneous type of pairwise interaction, two methoxy groups participate in the interactions along the *c*‐axis, whereas four methoxy groups are involved along the *a*‐axis. In addition, π–π interaction of the dibromophenylene units was observed along the *a*‐axis, with an interplanar separation of approximately 3.5 Å. Consistent with these observations, crystal framework calculations suggest strong interactions along the *a*‐axis, in good agreement with the observed anisotropy in crystal morphology (Figure S18, Table S4). While no significant short contacts involving bromine atoms are observed, the bromine atoms are located in close proximity within the crystal lattice. Compared with electrostatic interactions, dispersion forces operate over relatively longer distances and are therefore considered to influence the aggregation state [31, 32, 33]. Overall, all intermolecular interactions present in **α1** are classified as homotypic in nature.

Figure 2b shows the single‐crystal x‐ray structure of the **β1** polymorph (Table S5; CCDC dep. no.: 2523857, monoclinic, *P*2_1_/*c*). A unique crystal morphology is observed for **β1**, with several crystal faces indexed as high‐index planes. The torsion angle between the acrylonitrile and trimethoxyphenyl moieties (C^7–^C^8–^C^9–^C^10^) is 16.7°, which is close to that observed for C^1–^C^2–^C^3–^C^4^ in **α1**. By contrast, the acrylonitrile–dibromophenyl dihedral angle (C^9–^C^10–^C^11–^C^12^) reaches 50.2°, resulting in a substantial deviation from planarity. Relative to **1** in **α1**, theoretical calculations indicate that **1** in **β1** is less stable, with an estimated energy difference of approximately 5 kJ mol^−1^ (Figure S18). The relative energy differences between conformers fall within the tolerable range for polymorph formation [34, 35]. In **β1**, compound **1** forms a complex framework, in which heterotypic methoxy–methoxy interactions and halogen bonds are particularly noteworthy. Along the *b*‐ and *c*‐axes, the terminal 3‐ and 4‐methoxy groups on one molecule are positioned in close proximity to the 5‐methoxy group of a neighboring molecule, forming a chelate‐like interaction motif (Figure 2). The corresponding CH_3_–O···OCH_2_–H···O–CH_3_ intermolecular distances are 2.68 and 2.63 Å, respectively, which are comparable to those observed in **α1**. However, these pairwise interactions cannot be classified as homotypic because the number of participating methoxy groups differs. In addition, along the *a*‐axis, a contact is observed between the oxygen atom of the 3‐methoxy group and a bromine atom. The corresponding interatomic distance is 3.05 Å, which is shorter than the total of the van der Waals radii of oxygen (1.52 Å) and bromine (1.85 Å) [36]. The nearly linear C–Br···O angle of 173.6° indicates a halogen‐bonding interaction mediated by the σ‐hole on the bromine atom [37]. Accordingly, **β1** is inferred to construct heterotypic interactions of moderate strength along the *a*‐, *b*‐, and *c*‐axes, consistent with the crystal framework calculations (Figure S19 and Table S6).

The results of the conformational analysis indicate that the kinetically formed **α1** possesses a slightly more favorable conformational energy, with an estimated difference of approximately 5 kJ mol^−1^. However, crystal framework calculations for both polymorphs indicate comparable framework strengths, with no significant energetic preference. These results suggest that the energetic difference between the two polymorphs is small and cannot be clearly resolved by the present calculations. On this basis, **α1** is considered to be formed under kinetic control, whereas **β1** appears as a relatively more persistent phase under the present experimental conditions. This interpretation is supported by the observation that **β1** is obtained upon thermal or mechanical treatment and is not readily converted back to **α1** under the examined conditions. The energetic difference between the two polymorphs is sufficiently small that it cannot be resolved by crystal framework calculations alone, suggesting that the observed polymorphism arises from a delicate balance between kinetic and thermodynamic factors.

**FIGURE 3:**
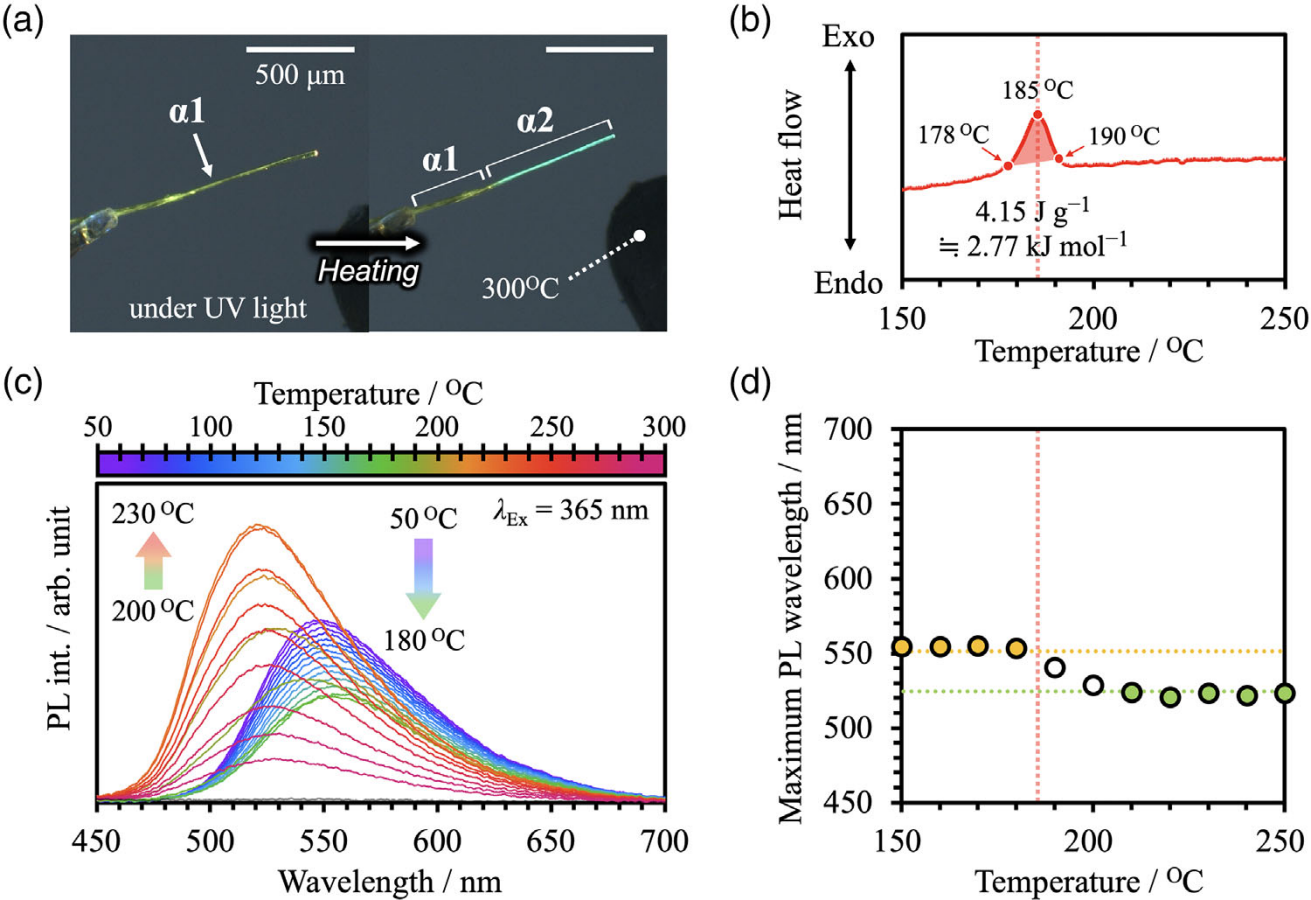
Solid‐state phase transition and luminescence changes. (a) Optical microscope images of **α1** under UV irradiation before and after spot‐heating. (b) DSC trace of **α1**. (c) Temperature‐dependent PL spectra of **α1** during the heating process. (d) Temperature–maximum PL wavelength profile of **α1**.

Herein, the aforementioned hierarchical model is visualized through a photoluminescent response by monitoring the physical behavior of the crystals under external stimuli. Fluorescence microscopy revealed that bringing a heat source into proximity to **α1** induces a partial solid‐state phase transition to a green‐emissive phase (**α2**) (Figure 3a). Notably, the thermally induced **α1** → **α2** transition is irreversible under the present experimental conditions. The observations suggest that α1 behaves as a metastable solid phase under the present experimental conditions, whereas **α2** represents a more persistent phase that is not readily converted back to **α1**. However, because no reverse transition from **α2** to **α1** was observed under the examined conditions, the present results do not unambiguously establish the thermodynamic stability relationship between the two phases, and kinetic inaccessibility of the reverse transformation cannot be excluded. Differential scanning calorimetry (DSC) of **α1** revealed an exothermic peak at approximately 180°C corresponding to the phase transition, with an enthalpy change of approximately 2.8 kJ mol^−1^ (Figure 3b). Heating‐rate‐dependent DSC measurements and Kissinger analysis support a cooperative phase‐transition mechanism, with no signatures indicative of solvation or desolvation processes (Figures S20 and S21). Cyclic DSC measurements over multiple heating–cooling cycles show no reproducible heat flow corresponding to a reverse transition, confirming the irreversible nature of this process (Figure S22). Moreover, by tracking the phase transition using temperature‐dependent solid‐state PL spectra, a dramatic change in the maximum PL wavelength was observed in the temperature range of 180°C–200°C (Figure 3c and S23). These results collectively demonstrate a clear correlation between thermal stimuli and the PL properties (Figure 3d).

The PL spectrum of **α2** closely matches that of **β1** (Figure S24). Single‐crystal x‐ray diffraction analysis of **α2** revealed that its crystal system, space group, and lattice parameters are nearly identical to those of **β1**, a conclusion further supported by powder x‐ray diffraction results (Table S7, Figures S25 and S26). Interestingly, analysis of the crystal face indexing and the polarization dependence of the PL intensity revealed that the molecular orientation changes significantly before and after the phase transition (Figures S27 and S28). The thermally induced single‐crystal‐to‐single‐crystal phase transition is consistent with **β1** (= **α2**) representing a more persistent phase under the examined conditions. Therefore, the hierarchical model based on homogeneous/heterogeneous interactions is consistent with both the experimental observations and theoretical calculations, supporting its usefulness as a qualitative framework for interpreting the observed phase‐transition behavior.

**FIGURE 4:**
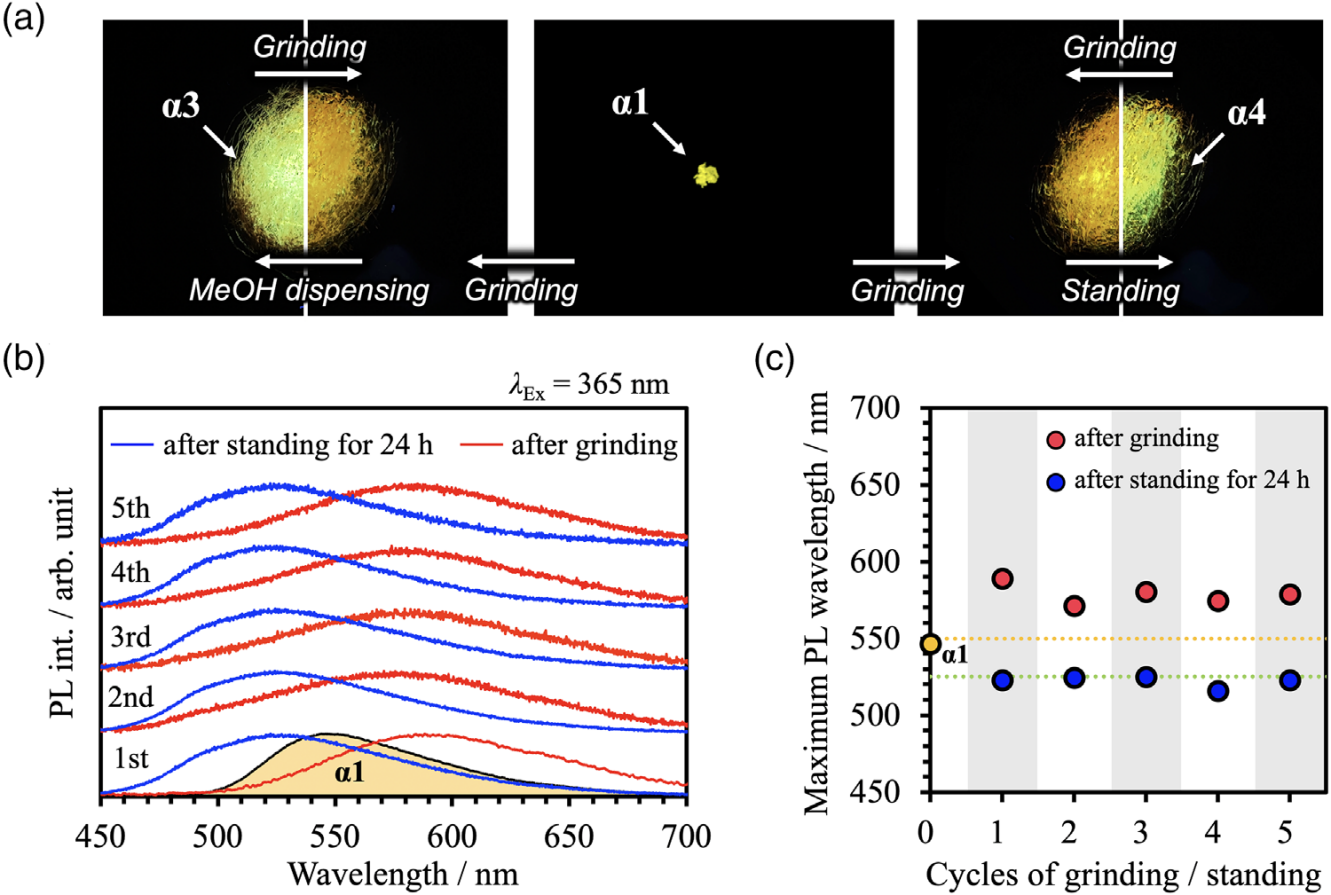
Mechanically‐induced PL changes. (a) Optical microscopy images of **α1**, ground **α1**, **α3**, and **α4**. (b) PL spectra of the freshly ground **α1** powder and **α4** obtained after standing for 24 h. Grinding **α4** resulted in similar behavior. (c) Plot of the maximum PL wavelength of the powder after grinding and standing.

Having clarified the hierarchical relationship between **α1** and **β1**, the mechanoresponsive luminescent response was examined in detail to explore analogous behavior under a different external stimulus. Mechanical grinding of **α1** resulted in a transformation into an orange‐emissive powder (Figure 4a). This powder exhibits an emission maximum at approximately 590 nm, suggesting an amorphous‐like aggregated state distinct from those of **α1** and **β1** (Figure S29). Powder x‐ray diffraction patterns of the ground sample show no discernible diffraction peaks, and DSC measurements reveal no thermal events associated with phase transitions, indicating the absence of long‐range crystalline order (Figure S30). The diffraction patterns of the ground sample also show no discernible diffraction peaks (Figure S31), indicating the absence of long‐range crystalline order. These results support the assignment of the ground powder as an amorphous‐like aggregated state. This behavior is consistent with emission from disordered aggregates with enhanced intermolecular electronic coupling. Mechanochemical experiments performed under a nitrogen atmosphere showed emission behavior identical to that observed in air, indicating that oxidation or hydration effects are negligible (Figure S31). Although direct identification of the aggregate structure was not achieved, the results suggest that mechanical compression induces disordered amorphous‐like aggregation states accompanied by changes in intermolecular distances and local molecular arrangements, thereby giving rise to altered luminescence properties [33, 38, 39, 40]. Next, when methanol—a poor solvent for **1—**was dropped onto the ground powder, it immediately transformed into a green‐emissive solid phase (**α3**). Similarly, standing the ground powder under vacuum in the dark resulted in its conversion into another green‐emissive solid phase (**α4**) (Figure S32). Under solvent‐free conditions, the transformation required up to 24 h, suggesting that molecular density and molecular rearrangement govern the timescale of the phase transition. Because both **α3** and **α4** reverted to an orange‐emissive powder upon grinding, the aggregation state and luminescent properties of the amorphous‐like powder are independent of the initial state. The emission color change induced by the grinding/standing process is pseudo‐reversible and exhibits mechanoresponsive luminescent behavior over up to five cycles (Figure 4b). Here, the term “pseudo‐reversible” refers to the recovery from the amorphous‐like powder to crystalline phases (**α3**/**α4**) upon standing, rather than a direct interconversion between **α1** and **β1**. Monitoring the emission maxima shows that those of **α3**/**α4** are close to, but not identical to, those of **β1** (Figure 4c). Specifically, **α1** exhibits an emission maximum at 550 nm, **β1** at 525 nm, and **α3**/**α4** in the range of approximately 530–535 nm. The slight discrepancy arises from a characteristic shoulder in the **α3**/**α4** spectra on the short‐wavelength side, which affects the apparent peak position. In addition, the excitation spectra of **α3**/**α4** closely resemble that of **β1**, further supporting their structural similarity. Powder x‐ray diffraction analysis of **α3** and **α4** verified that both exhibit diffraction patterns consistent with that of **β1** (Figure S33), further supporting that these phases correspond to the same or closely related structural state. Therefore, these results suggest that the mechanically induced transformation pathway from **α1** involves an amorphous‐like intermediate state prior to the formation of **β1**‐like crystalline phases. Although the present observations establish this pathway for **α1**‐derived samples, whether an identical mechanochemical process operates directly from **β1** remains unclear. Notably, **α1** is not regenerated from **β1** or from the amorphous intermediate under the present conditions, indicating that the mechanochemical process does not constitute a fully reversible **α1** ↔ **β1** cycle.

**FIGURE 5:**
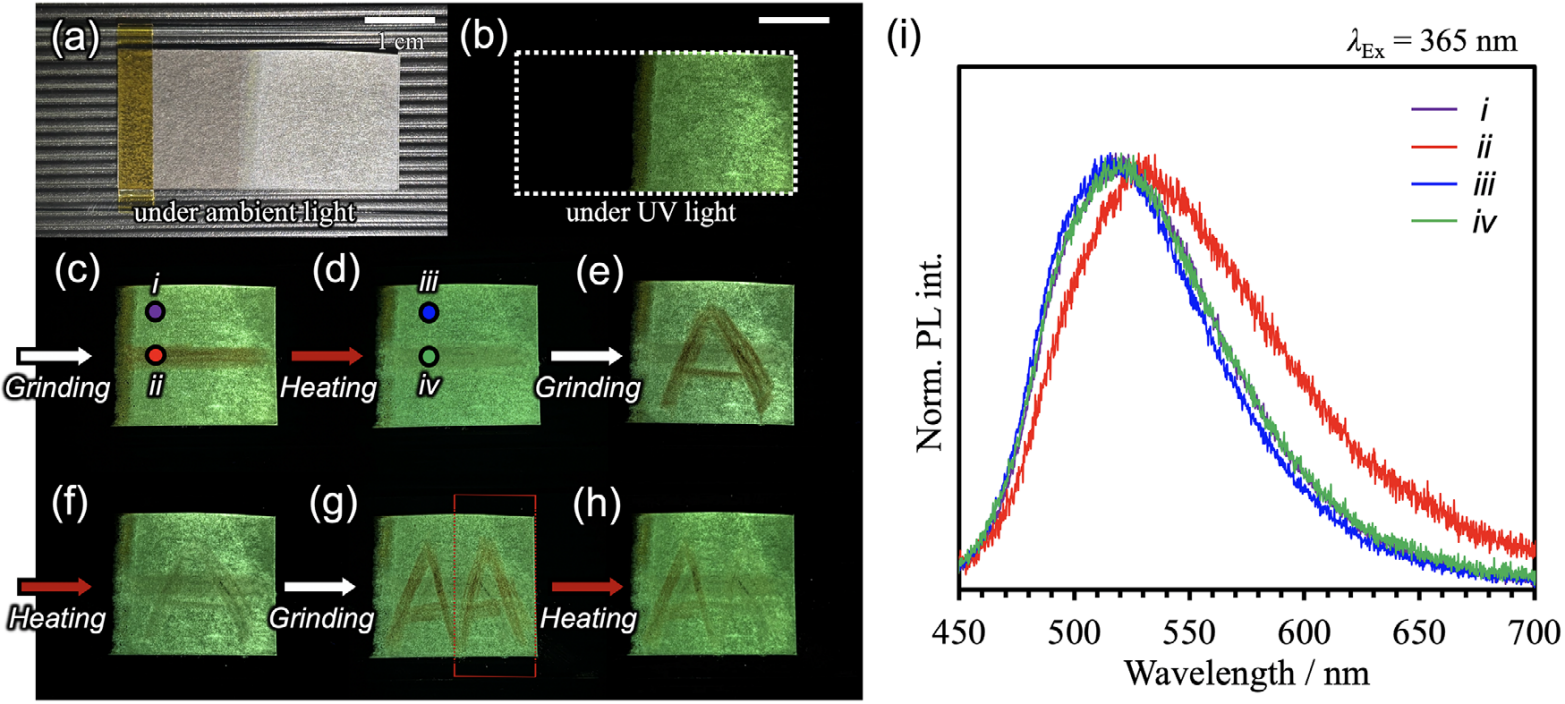
Secret paper application. (a) Photographs of the sample paper under ambient light (b) under ambient light. (c) Scraping the sample with a spatula induces a change in emission color, (d) whereas heating restores the original emission. (e, f) This cycle can be repeated, and (g, h) localized heating allows selective recovery of the original emission color only within the heated region. (i) PL spectra recorded at positions *i–iv*.

Finally, we demonstrate a secret paper application that exploits the phase‐transition characteristics. A sample paper was prepared by impregnating black drawing paper with a chloroform solution of **1**, followed by drying at room temperature (Figure 5a). Under UV irradiation, the impregnated regions exhibited green emission originating from **β1** (Figure 5b). The preferential precipitation of **β1** is attributed to the heterogeneous and spatially confined crystallization environment provided by the porous cellulose network of the paper substrate. In contrast to bulk solution crystallization, the substrate introduces multiple nucleation sites and locally restricted diffusion fields, which are known to influence polymorph selection. Given that **β1** represents the relatively stable phase in this system, such conditions likely suppress the formation of kinetically favored **α1** and promote nucleation and growth of **β1**. Mechanical rubbing of the sample paper with a spatula induced a change to orange emission (Figure 5c). Because the molecular motion of **1** is severely reduced within the paper matrix, this emission change can be maintained for more than 3 days at room temperature. Although the dispersed amorphous powder does not recover upon standing, heating the sample paper at 100°C restores the original green emission (Figure 5d). This process is repeatable using the same sample paper, and localized heating enables partial recovery of the emission (Figure 5e–h). Solid‐state PL spectra recorded for the rubbed regions exhibited a red‐shifted emission maximum along with a weak shoulder at approximately 625 nm (Figure 5i). This feature is attributed to the contribution from emissions associated with an amorphous‐like powder generated by mechanical stimulation.

## 3 Conclusion

Understanding phase transition behavior is expected to contribute not only to advanced security materials but also to the rational design of dynamic functions across physics, materials science, and the life sciences. In this work, we investigated polymorphic structure–property dynamics associated with competing intermolecular interactions in molecular crystals. By classifying noncovalent interactions according to their energetic contributions, the results suggest that subtle changes in intermolecular interaction patterns are associated with distinct phase‐transition behaviors and photophysical responses. The present study reveals that even small differences in thermodynamic hierarchy are closely associated with phase transition pathways and associated photophysical responses in molecular solids. The present case study highlights how multifunctional molecular side chains can generate competing intermolecular interaction motifs within a single molecular framework. Overall, our results show how subtle side‐chain modifications within a single molecular framework can modulate structural diversity and dynamic behavior in molecular crystals.

## Author Contributions

**Mahiro Nakabayashi**: conceptualization, investigation, writing – original draft, formal analysis, visualization, methodology. **Shotaro Hayashi**: conceptualization, writing – original draft, funding acquisition, visualization, writing – review and editing, resources.

## Conflicts of Interest

The authors declare no conflicts of interest.

## Supporting information




**Supporting File**: anie73282‐sup‐0001‐SuppMat.pdf.

## Data Availability

The data that support the findings of this study are available from the corresponding author upon reasonable request.
