# Supplementary material for: Interaction Hierarchy and Polymorphic Structure–Property Dynamics in Luminescent Molecular Crystals
Source: Angew Chem Int Ed Engl. 2026 Jun 30;65(36):e8807652. doi: 10.1002/anie.8807652 (PMC13528507; doi:10.1002/anie.8807652)
Supplement: Supplementary file 1 [file ANIE-65-e8807652-s001.pdf]

Supporting Information

Interaction Hierarchy and Polymorphic Structure–Property Dynamics in Luminescent Molecular Crystals

Mahiro Nakabayashi,*[a] and Shotaro Hayashi*[a,b]

[a] M. Nakabayashi, S. Hayashi
School of Engineering Science
Kochi University of Technology
185, Miyanokuchi, Tosayamada, Kami, Kochi, 782-8502
E-mail: mahiro0915@icloud.com; hayashi.shotaro@kochi-tech.ac.jp

[b] S. Hayashi
FOREST Center, Research Institute
Kochi University of Technology
185, Miyanokuchi, Tosayamada, Kami, Kochi, 782-8502

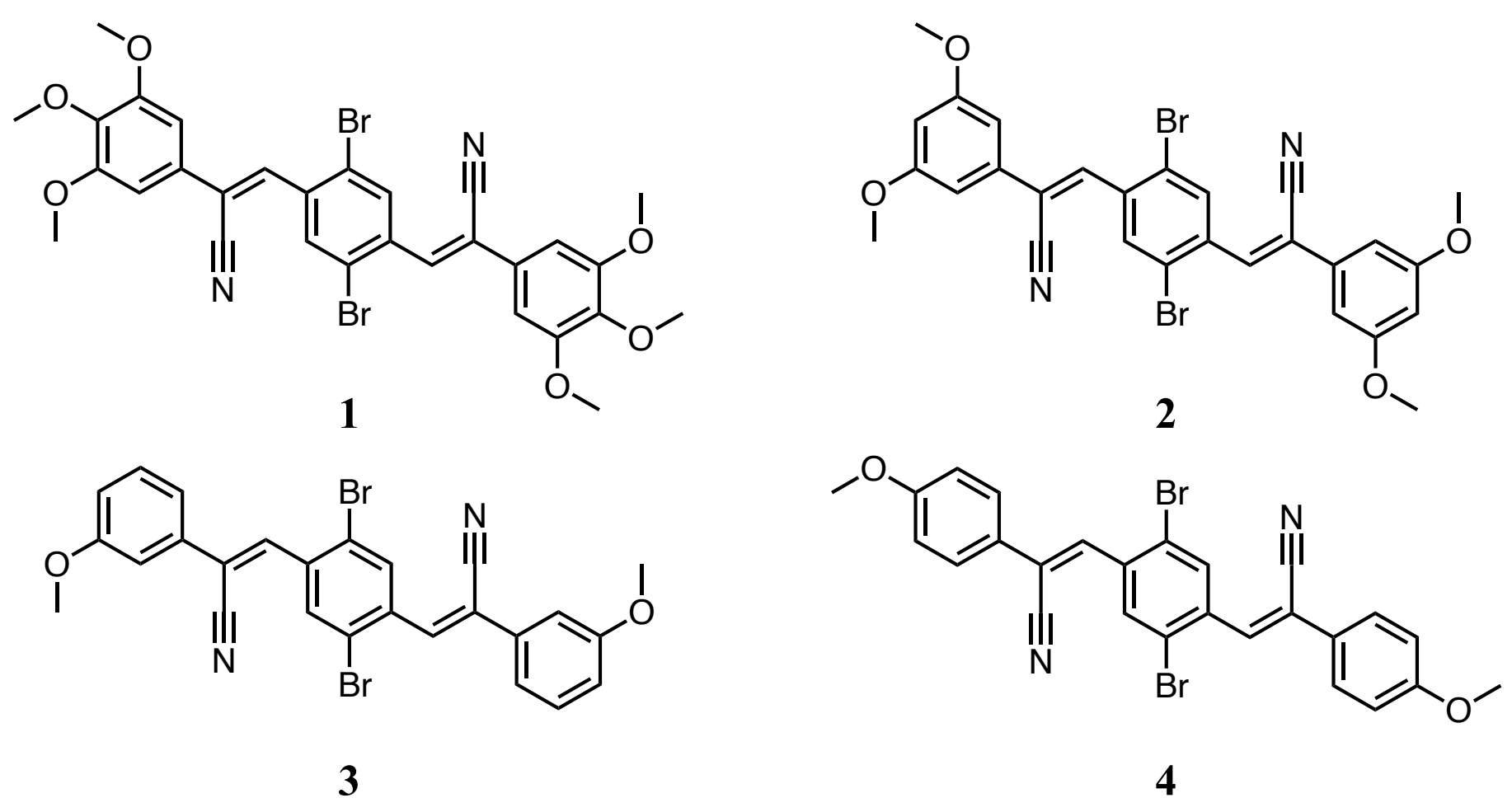

Chart S1. Chemical structures of synthesized CDSB derivatives **1–4**.

Chemicals

Terephthalaldehyde (TCI), *N*-bromosuccinimide (TCI), 3,4,5-trimethoxyphenylacetonitrile (TCI), 3,5-dimethoxyphenylacetonitrile (TCI), 3-methoxyphenylacetonitrile (TCI), 4-methoxyphenylacetonitrile (TCI), 4-(4,4,5,5-tetramethyl-1,3,2-dioxaborolan-2-yl)pyridine (TCI), sulfuric acid (KANTO), 28% sodium methoxide methanol solution (KANTO), anhydrous magnesium sulfate (Hayashi Pure Chemical Industry), methyl methacrylate polymer (TCI), Rhodamine B (TCI) and ethylene glycol (TCI) were used as received. Solvents were used as received.

Synthesis

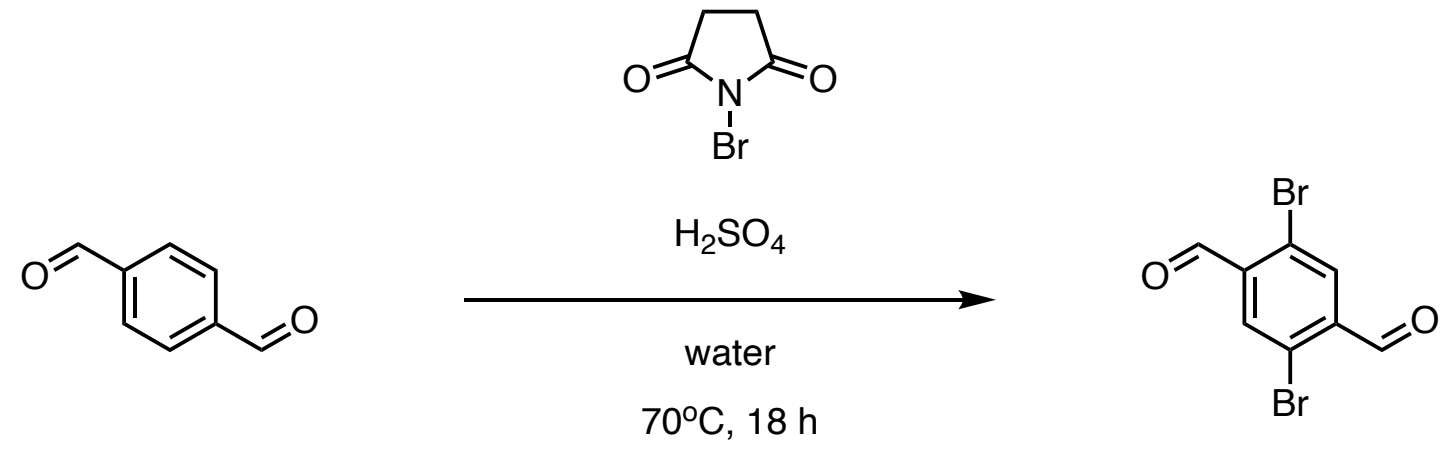

Scheme S1. Synthesis of molecule 2,5-dibromoterephthalaldehyde.

In a 100 mL flask, terephthalaldehyde (2.7 g, 20 mmol) was dissolved in concentrated sulfuric acid (26 mL) at 70°C. *N*-Bromosuccinimide (8.5 g, 48 mmol) was added to the solution over 40 minutes and heated at 70°C for 18 hours. After cooling to room temperature, the mixture was neutralized with aqueous sodium hydrogen carbonate. Then, extraction was carried out with dichloromethane. The organic layer was dried over anhydrous magnesium sulfate and concentrated in a vacuum. The product was purified by recrystallization in ethyl acetate at elevated temperature from 65°C to room temperature, resulting pale yellow solid (2.5 g, 8.7 mmol, 43%); ^{1}H NMR (400 MHz, $CDCl_3$) δ 10.35 (s, 2H), 8.16 (s, 2H); ^{13}C NMR (100 MHz, $CDCl_3$) δ 189.8, 137.3, 135.0, 125.5; HRMS (APCI/TOF) calcd. for $[M+H]^+$ $C_8H_5Br_2O_2$: 290.8651, found: 290.8624.

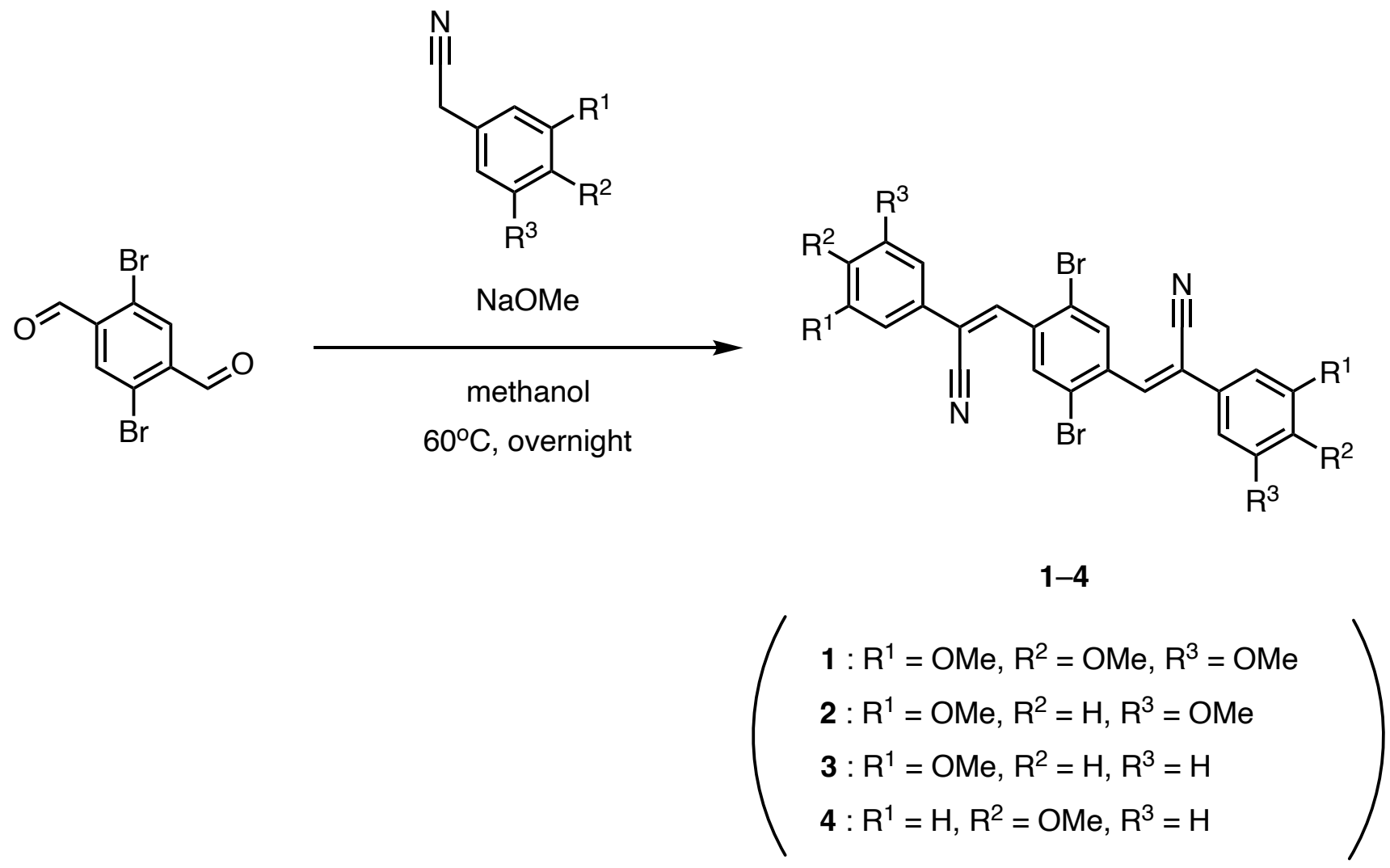

Scheme S2. Synthesis of **1–4**.

In a 50 mL flask, 2,5-dibromoterephthalaldehyde (146 mg, 0.50 mmol) and phenylacetonitrile derivative (1.50 mmol) were dissolved in methanol (3 mL). After the 60ºC was reached, 28% sodium methoxide methanol solution

(1 mL) was added to the solution and heated for overnight. After cooling to room temperature, the precipitated solid in the flask was separated by suction filtration, resulting in solid **1–4**. For ^{13}C NMR measurements, compounds **2** and **4** were not analyzed due to their extremely low solubility, which prevented the preparation of sufficiently concentrated solutions.

1. Yellow solid (329 mg, 0.49 mmol, 98%); ^{1}H NMR (400 MHz, $CDCl_3$) δ (ppm) = 8.33 (s, 2H), 7.65 (s, 2H), 6.92 (s, 4H), 3.96 (s, 12H), 3.92 (s, 6H); ^{13}C NMR (100 MHz, $CDCl_3$) δ (ppm) = 153.8, 146.6, 140.1, 137.8, 136.5, 133.2, 128.8, 124.0, 116.7, 103.9, 61.1, 56.4; HRMS (APCI/TOF) calcd. for $[M+H]^+$ $C_{30}H_{27}Br_2N_2O_6$: 669.0230, found: 669.0268.

2. Yellow solid (298 mg, 0.49 mmol, 97%); ^{1}H NMR (400 MHz, $CDCl_3$) δ (ppm) = 8.33 (s, 2H), 7.73 (s, 2H), 6.85 (d, J = 2.4 Hz, 4H), 6.56 (t, J = 2.4 Hz, 2H), 3.87 (s, 12H); HRMS (APCI/TOF) calcd. for $[M+H]^+$ $C_{28}H_{23}Br_2N_2O_4$: 609.0019, found: 608.9991.

3. Yellow solid (262 mg, 0.48 mmol, 95%); ^{1}H NMR (400 MHz, $CDCl_3$) δ (ppm) = 8.35 (s, 2H), 7.75 (s, 2H), 7.41 (dd, J = 8.0, 8.0 Hz, 2H), 7.32 (dd, J = 8.0, 2.0 Hz, 2H), 7.24 (dd, J = 2.0, 2.0 Hz, 2H), 7.01 (dd, J = 8.0, 2.0 Hz, 2H), 3.89 (s, 6H); ^{13}C NMR (100 MHz, $CDCl_3$) δ (ppm) = 160.2, 138.6, 136.6, 134.6, 133.3, 130.4, 124.1, 118.8, 116.6, 116.6, 115.8, 112.1; HRMS (APCI/TOF) calcd. for $[M+H]^+$ $C_{26}H_{19}Br_2N_2O_2$: 548.9808, found: 548.9801.

4. Yellow solid (256 mg, 0.47 mmol, 93%); ^{1}H NMR (400 MHz, $CDCl_3$) δ (ppm) = 8.34 (s, 2H), 7.67 (d, J = 9.2 Hz, 4H), 7.63 (s, 2H), 7.00 (d, J = 9.2 Hz, 4H), 3.88 (s, 6H); HRMS (APCI/TOF) calcd. for $[M+H]^+$ $C_{26}H_{19}Br_2N_2O_2$: 548.9808, found: 548.9802.

Polymer Matrix

Polymethyl methacrylate (1000 mg) and **1** (1 mg) were separately dissolved in chloroform, and the solutions were combined and thoroughly mixed. The resulting mixture was cast into a Teflon dish using a Pasteur pipette and dried at room temperature and atmospheric pressure for 24 hours, followed by vacuum drying for 24 hours to obtain a free-standing polymer matrix film.

Security Paper

Black drawing paper was impregnated with a chloroform solution of **1** (30 mg / 15 mL) and dried at room temperature. This procedure was repeated five times to prepare the sample paper.

General Information

Unless otherwise declared, experiments were all performed at room temperature (25±3°C). Liquid-state ^{1}H (400 MHz) and ^{13}C (100 MHz) nuclear magnetic resonance (NMR) spectra were recorded on a JEOL JNM-ECZ400S in chloroform-d_1 with 0.03% tetramethylsilane (Kanto Chemical Co.). Chemical shifts (δ) were expressed relative to the resonances of tetramethylsilane ^{1}H [Si(CH_3)$_4$: δ = 0.00 ppm] and ^{13}C [Si(CH$_3$)$_4$: δ = 0.00 ppm]. Mass spectral analysis was performed by compact QTOF (Bruker), and the data were analyzed with DataAnalysis 5.1. APCI-MS spectrum was measured using the direct insertion probe (Bruker). Calibration was performed with Tuning Mix (Agilent Technologies) in the range of 100 m/z to 3000 m/z. Optical and fluorescence microscope images were acquired using a Shimadzu STZ-171 equipped with a moticam1080BMH camera and a WUBEN E19-UV light source. Single-crystal X-ray diffraction data were collected on a Rigaku XtaLAB Synergy-S/Mo diffractometer equipped with Mo Kα radiation (λ = 0.71073 Å) using a crystal mounted on a loop at 113 K. Data collection was carried out using the CrysAlisPro[1] software package, and structure solution and refinement were performed with Olex2[2] as the graphical user interface. Initial phases were obtained by SHELXT[3], and the non-hydrogen atoms were refined anisotropically by full-matrix least-squares methods on F^2 using SHELXL[4]. Hydrogen atoms were placed in calculated positions and included in the structure factor calculations, but were not refined. Graphics of the crystal structure were represented using Mercury.[5] Powder X-ray diffraction measurements were performed on Rigaku SmartLab diffractometer with graphite-monochromatized Cu Kα radiation (λ = 1.5418 Å). Density functional theory: DFT calculation was performed using Gaussian 16[6] suit of programs by optimization using the B3LYP/6-31G(d) (C, H, N, O), LanL2DZ (Br, ECP) methods, respectively. The orbital diagrams were generated by using the GaussView 6.0[7] program. Energy calculation of crystal flamework was performed using CrystalExplorer.[8] Differential scanning calorimetry was measured by Shimazu DSC-69A Plus. Photoluminescence and excitation spectra were measured on JASCO FP-8500. The respective corrections were carried out using Rhodamine B in ethylene glycol and a secondary standard halogen light source, JASCO ESC-842, respectively. Emission decay profiles were measured using a Hamamatsu C5094 spectrometer coupled with a C4334 streak camera. The excitation light was generated from a Spectra-Physics Tsunami 3941-M1BB femtosecond Ti:sapphire oscillator using a GWU-UHG-2PSK-W pulse picker and frequency doubler. Temperature-dependent PL spectra were measured using an Ocean Optics LDC-1C as the excitation light source, an Ocean Optics P400-2-UV-VIS optical fiber for UV–vis transmission, and an Ocean Optics FLAME-T-UV-VIS spectrometer. Temperature control was achieved using a JAPAN HIGH TECH 10002L stage, and all measurements were performed under a nitrogen atmosphere. The polarization dependence of the PL intensity was measured using the setup shown in **Figure S25**.

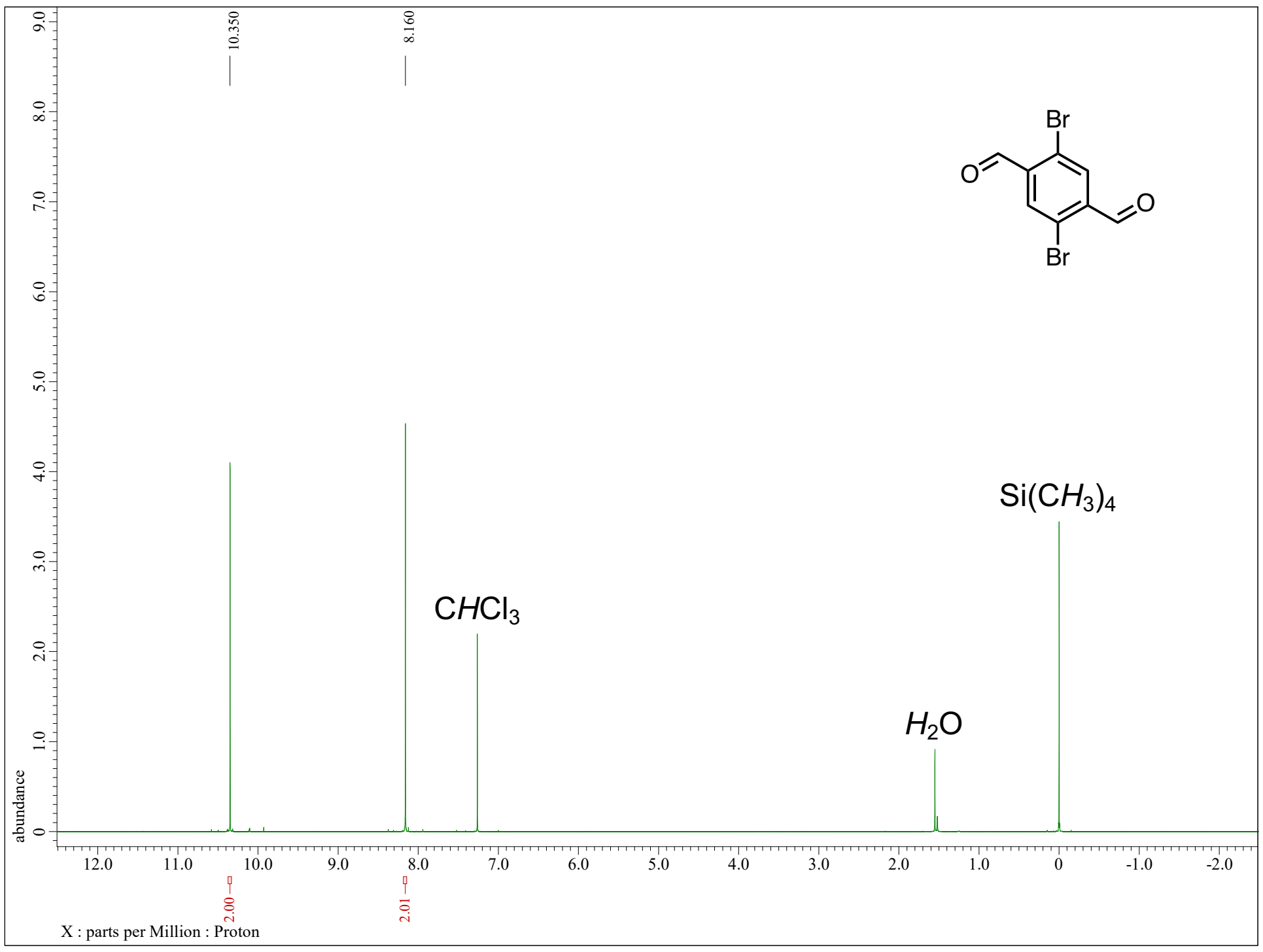

Figure S1. ^{1}H NMR spectrum of 2,5-dibromoterephthalaldehyde (400 MHz, in $CDCl_3$).

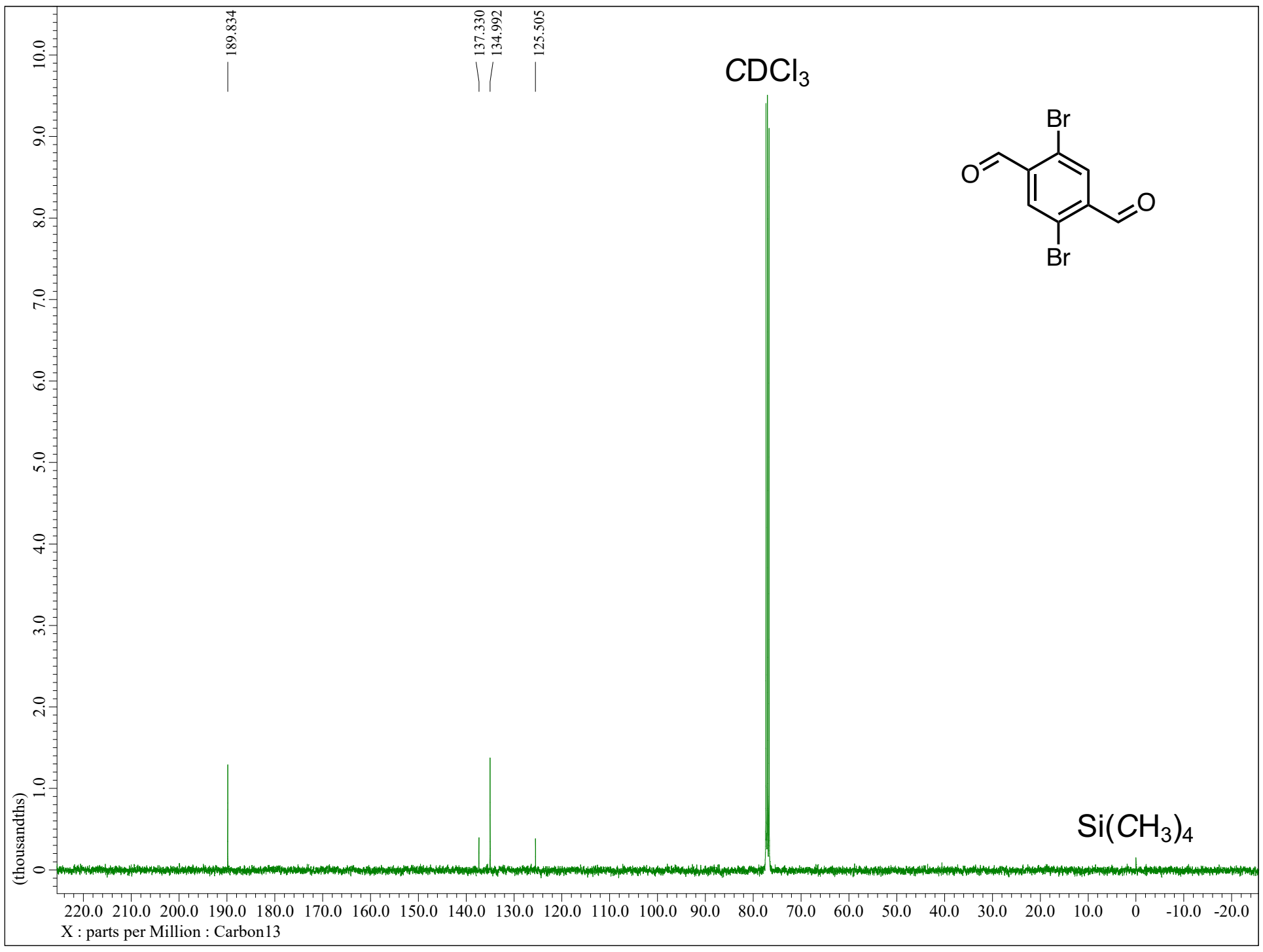

Figure S2. ^{13}C NMR spectrum of 2,5-dibromoterephthalaldehyde (100 MHz, in $CDCl_3$).

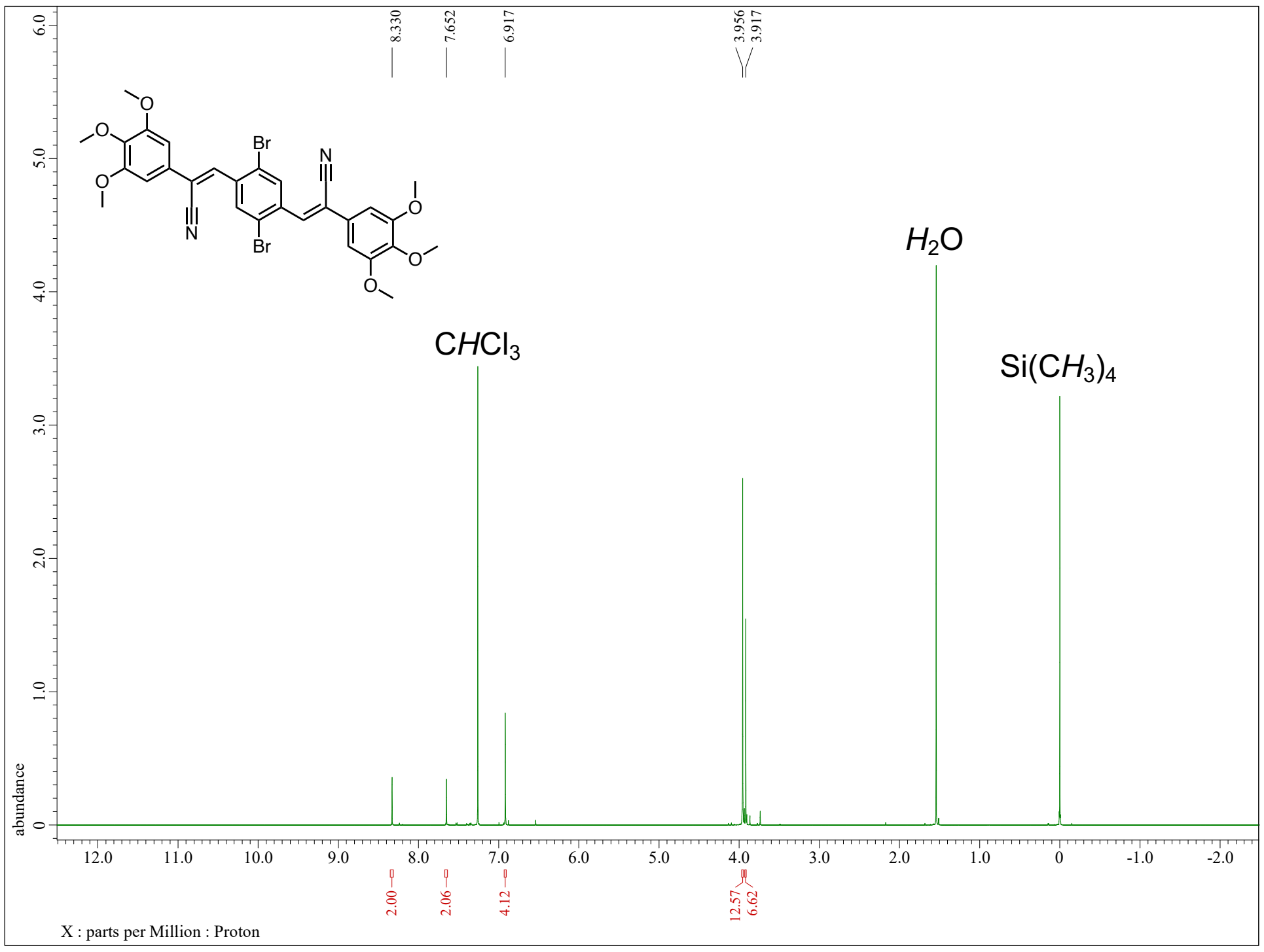

Figure S3. ^{1}H NMR spectrum of **1** (400 MHz, in $CDCl_3$).

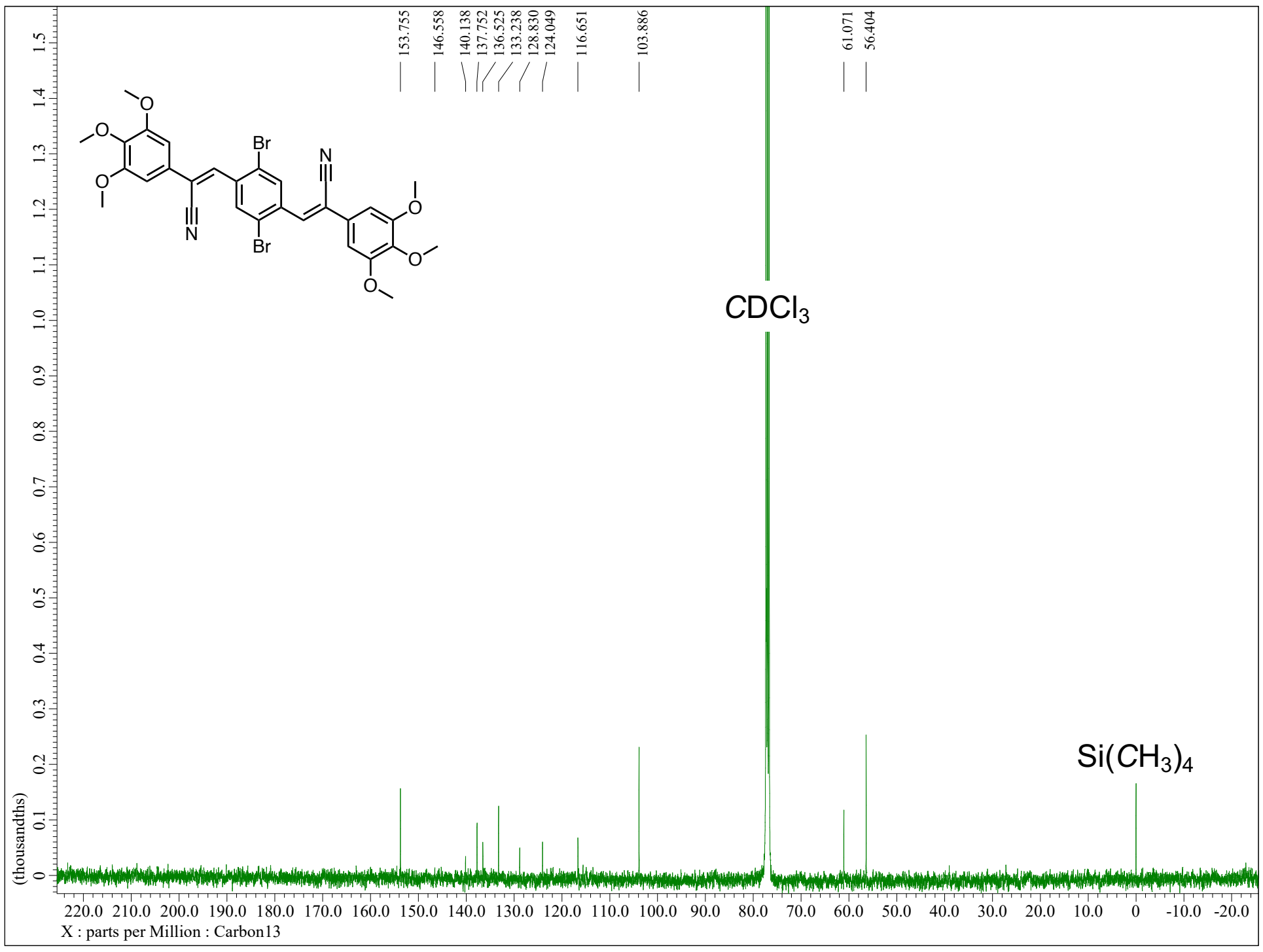

Figure S4. ^{13}C NMR spectrum of **1** (100 MHz, in $CDCl_3$).

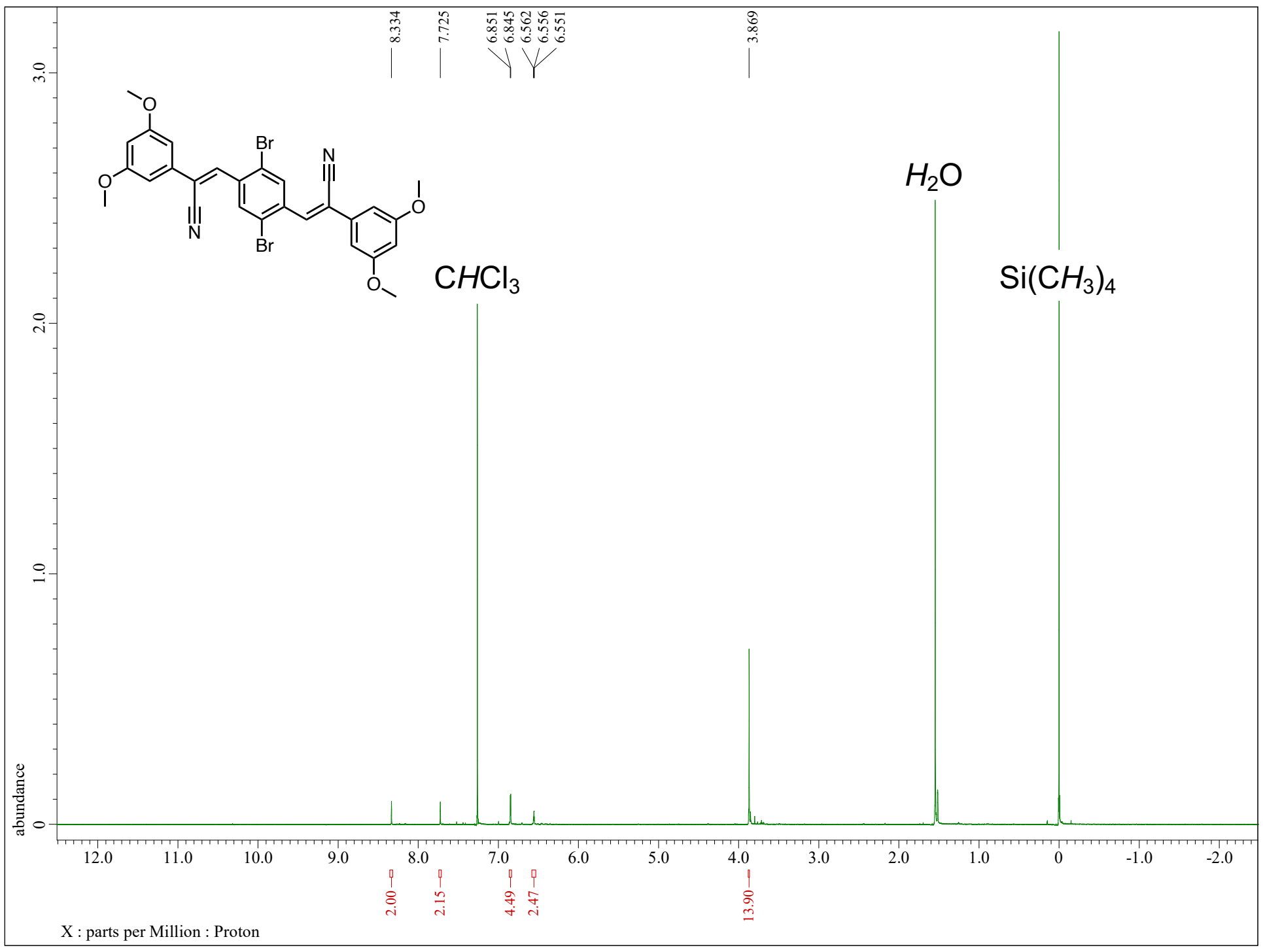

Figure S5. ^{1}H NMR spectrum of **2** (400 MHz, in $CDCl_3$).

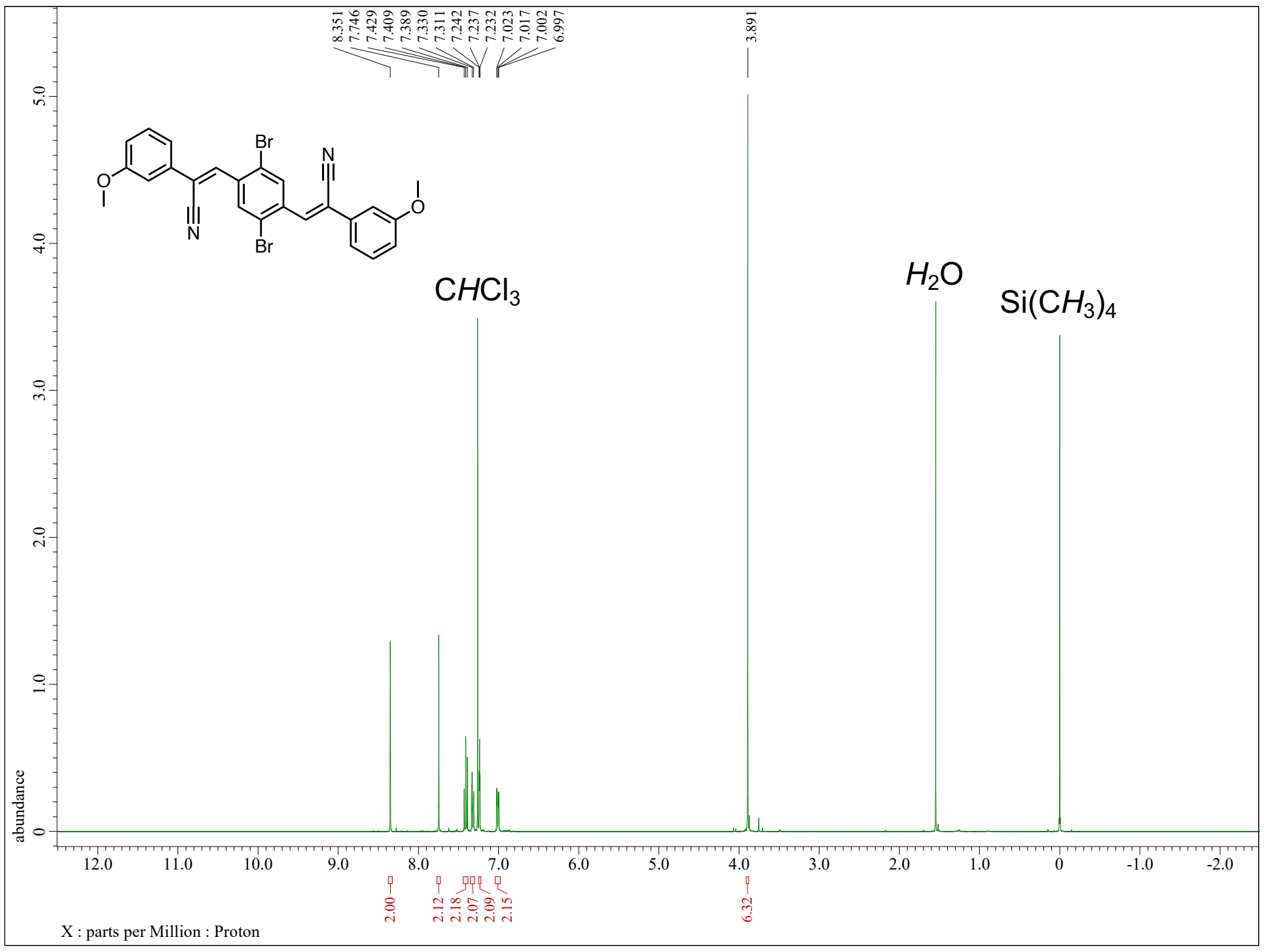

Figure S6. ^{1}H NMR spectrum of **3** (400 MHz, in $CDCl_3$).

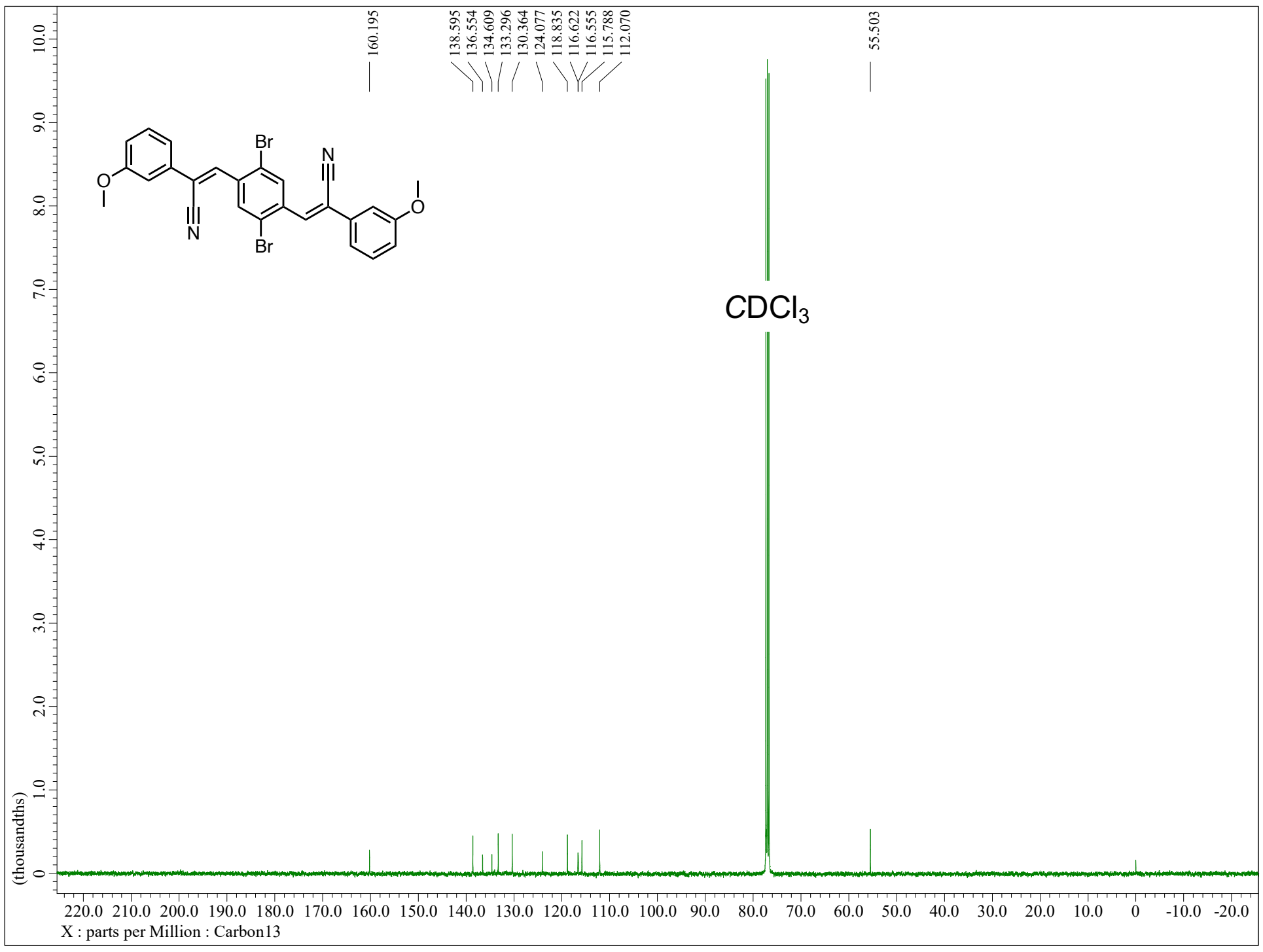

Figure S7. ^{13}C NMR spectrum of **3** (100 MHz, in $CDCl_3$).

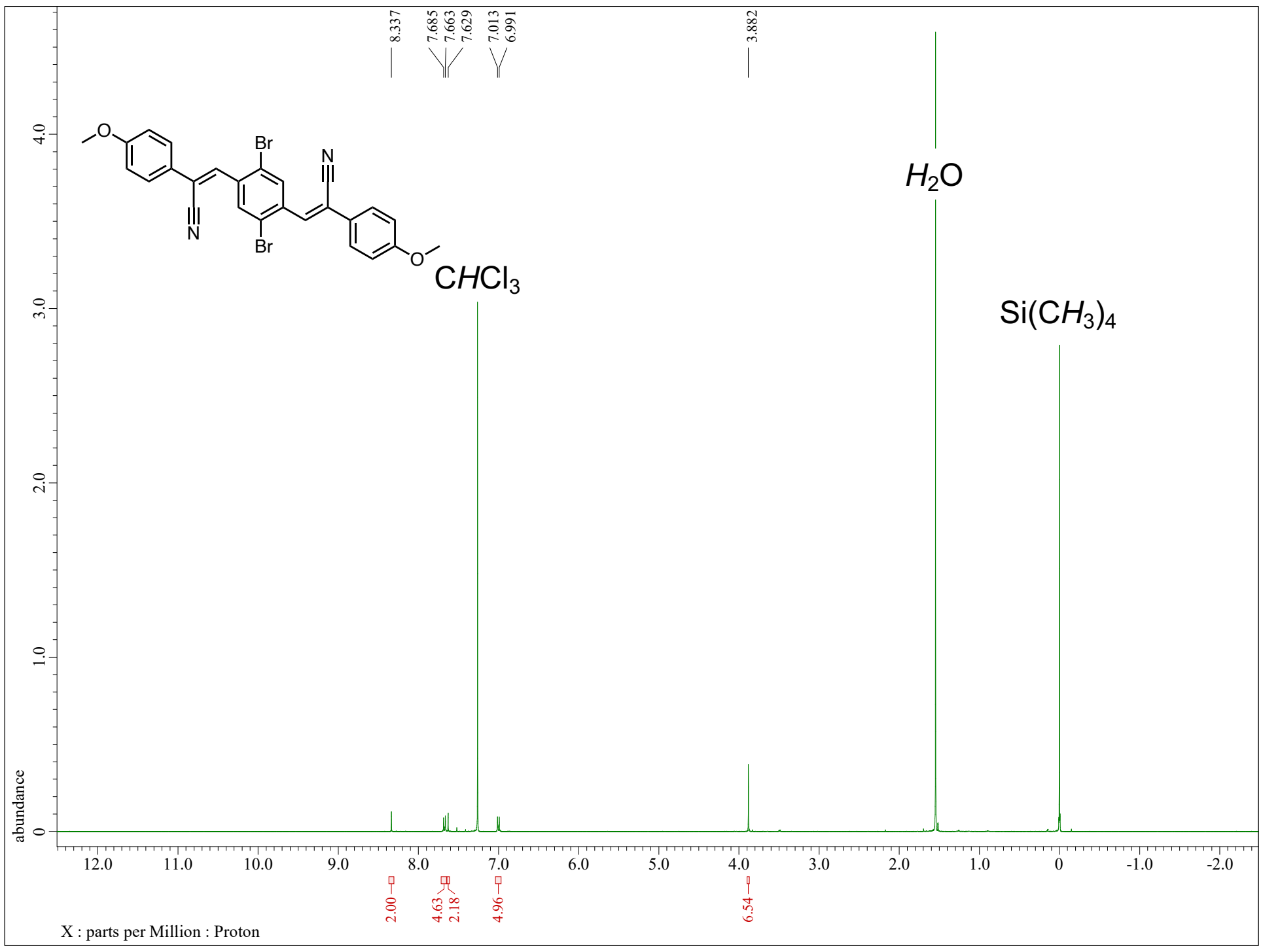

Figure S8. ^{1}H NMR spectrum of **4** (400 MHz, in $CDCl_3$).

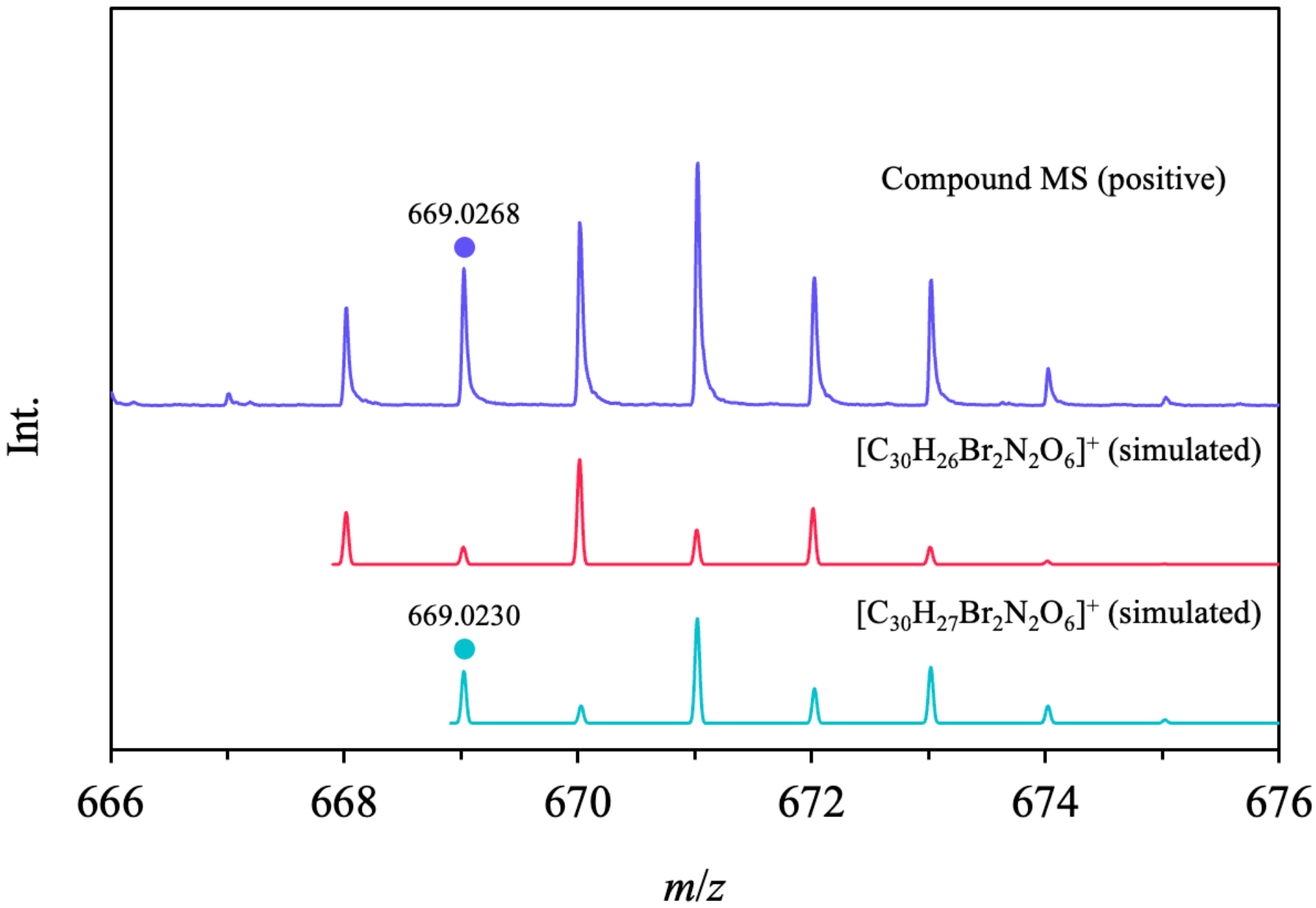

Figure S9. HR-MS spectrum of **1**.

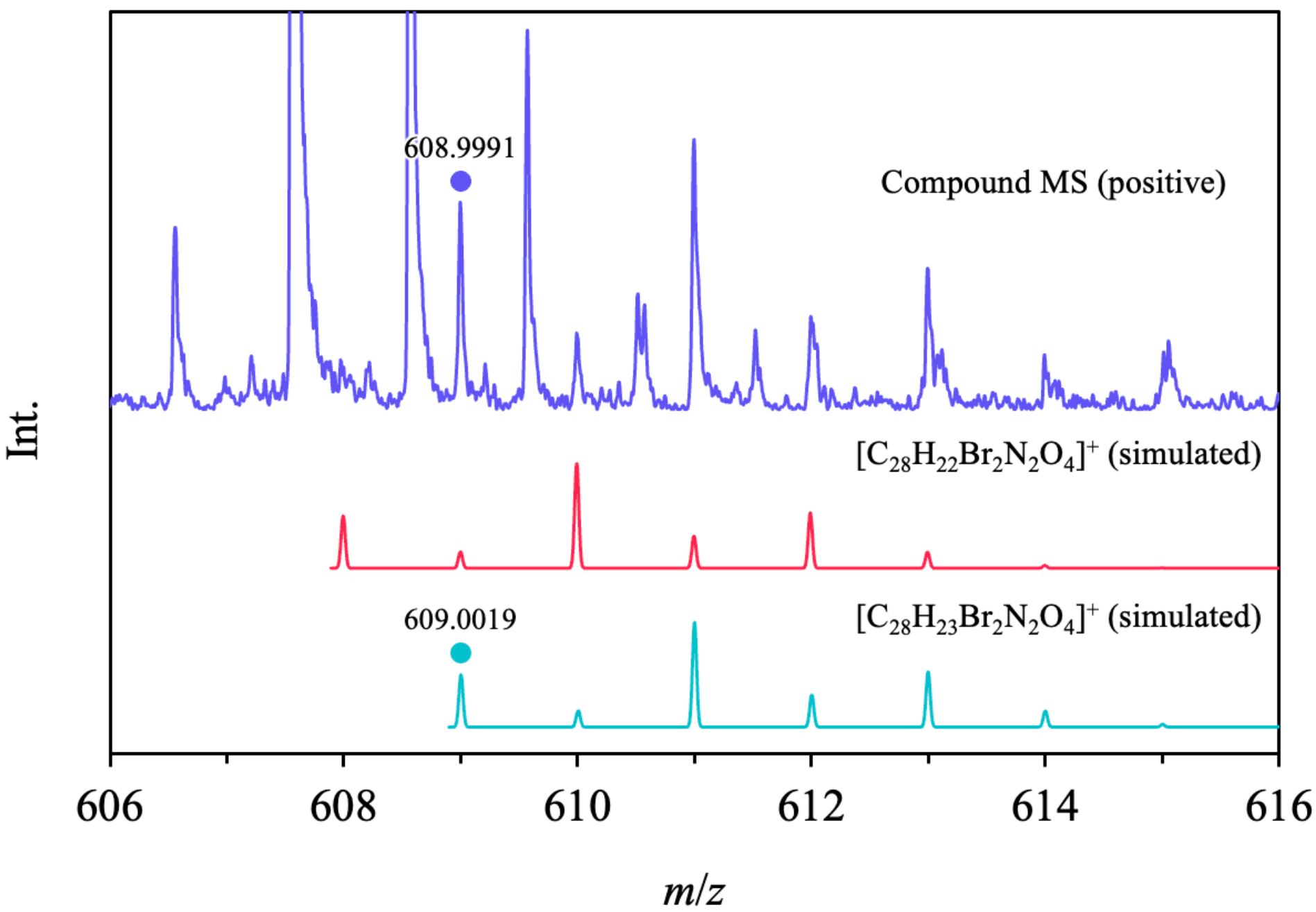

Figure S10. HR-MS spectrum of **2**.

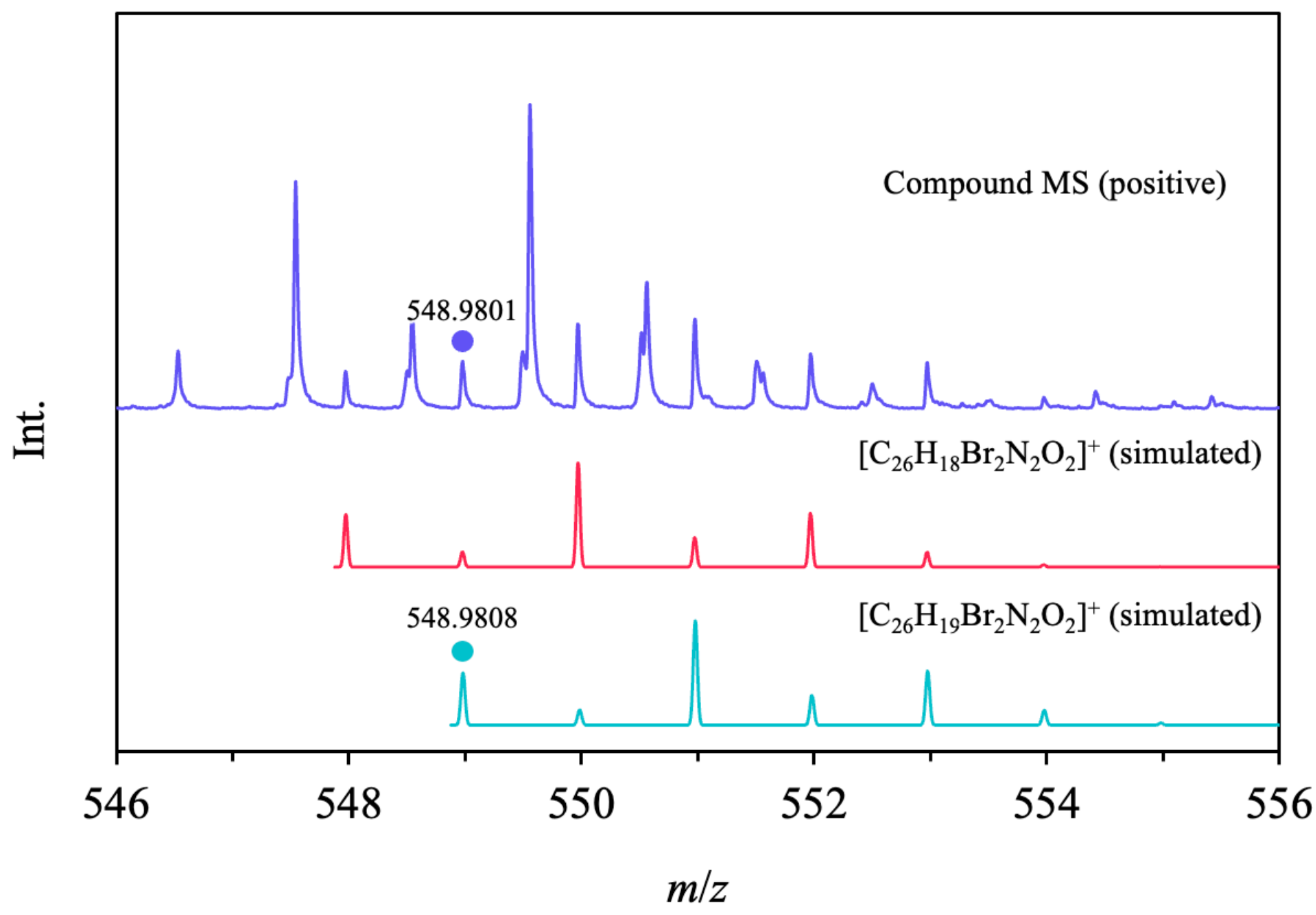

Figure S11. HR-MS spectrum of **3**.

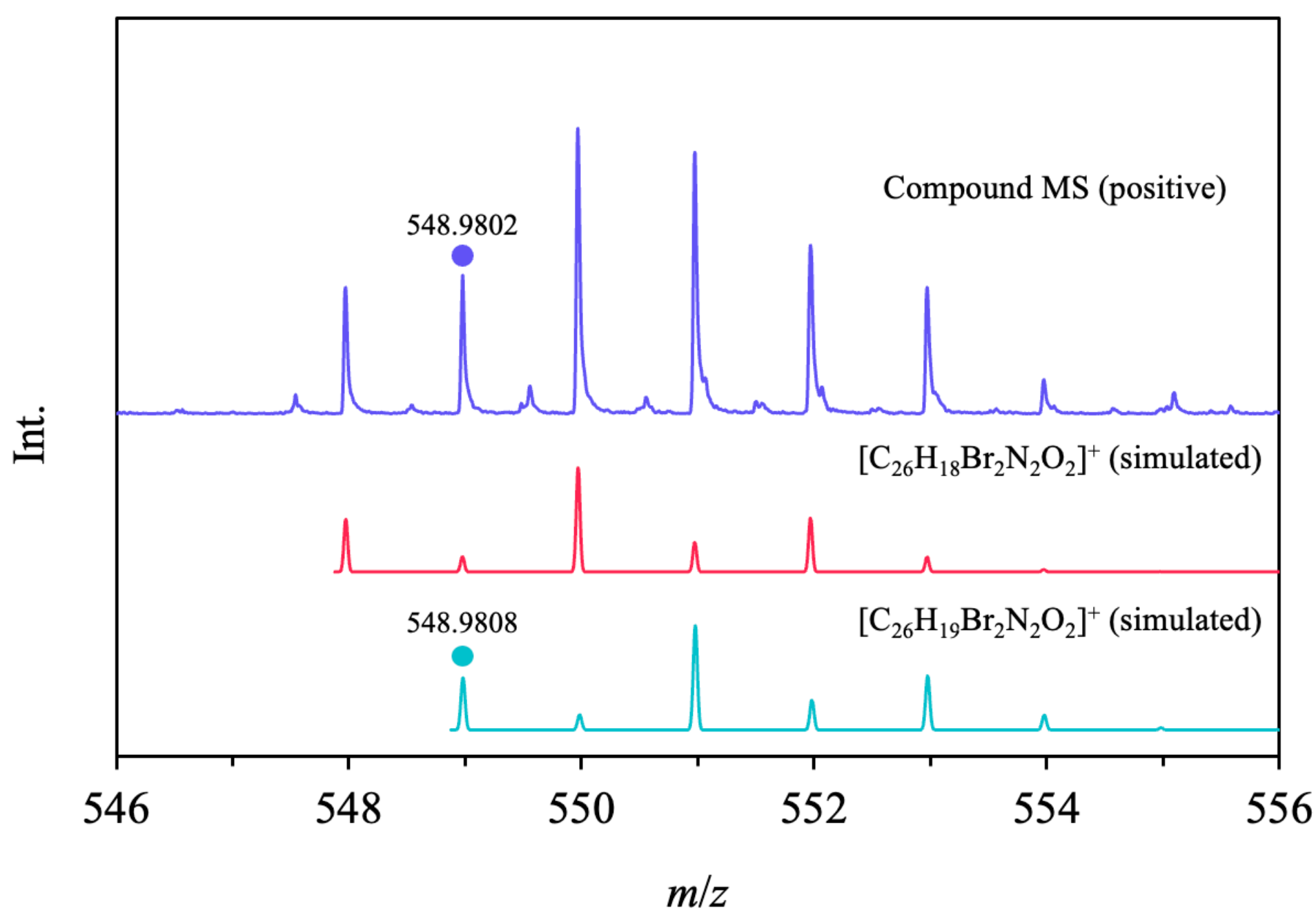

Figure S12. HR-MS spectrum of **4**.

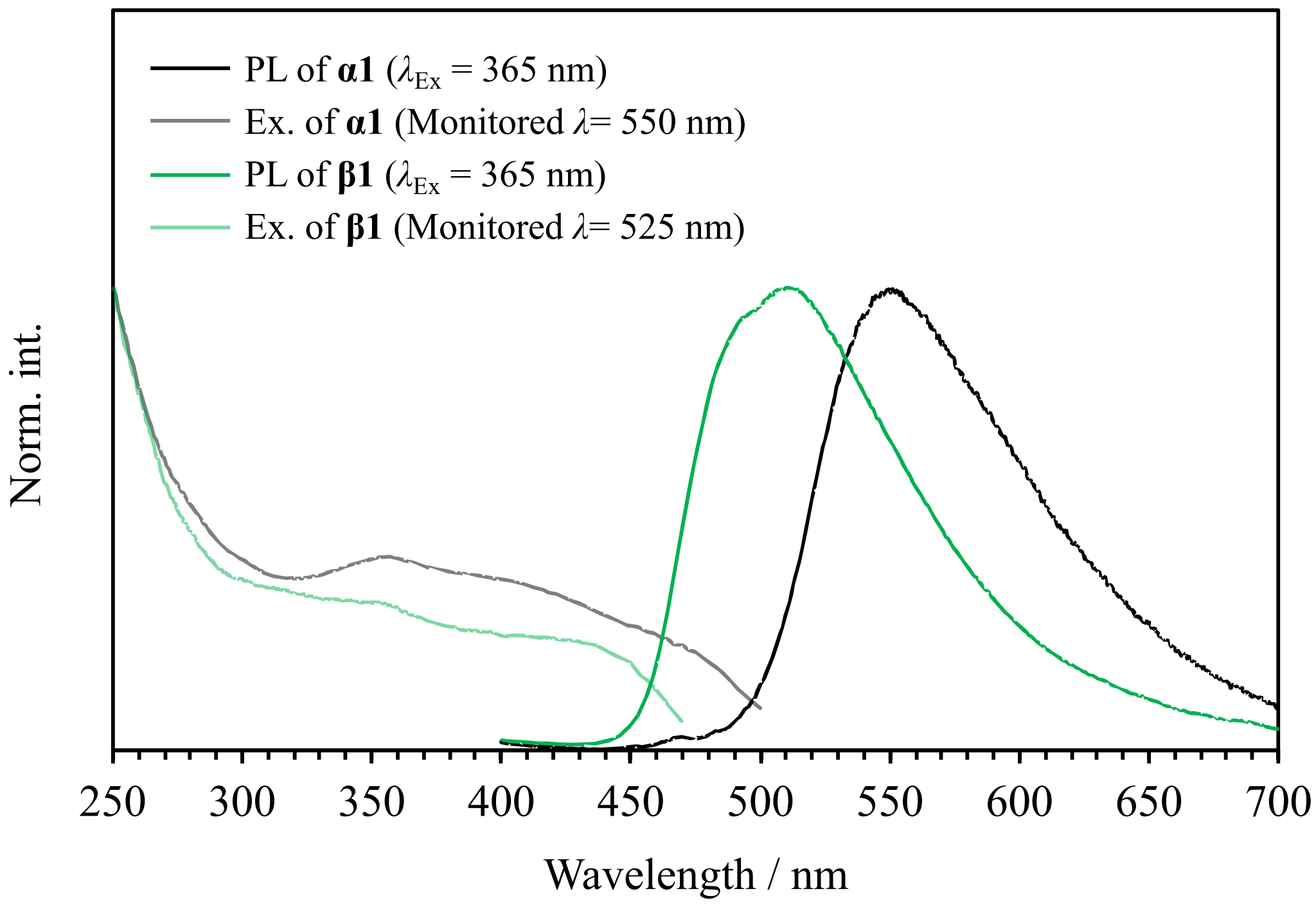

Figure S13. Photoluminescence (PL) and excitation (Ex.) spectra of **α1** and **β1**. The apparent emission maximum may vary slightly depending on the measurement conditions (e.g., instrumental setup and spectral processing), which can influence the spectral profile and peak position. The overall emission features, however, are consistent with those of the **β1** phase discussed in the main text.

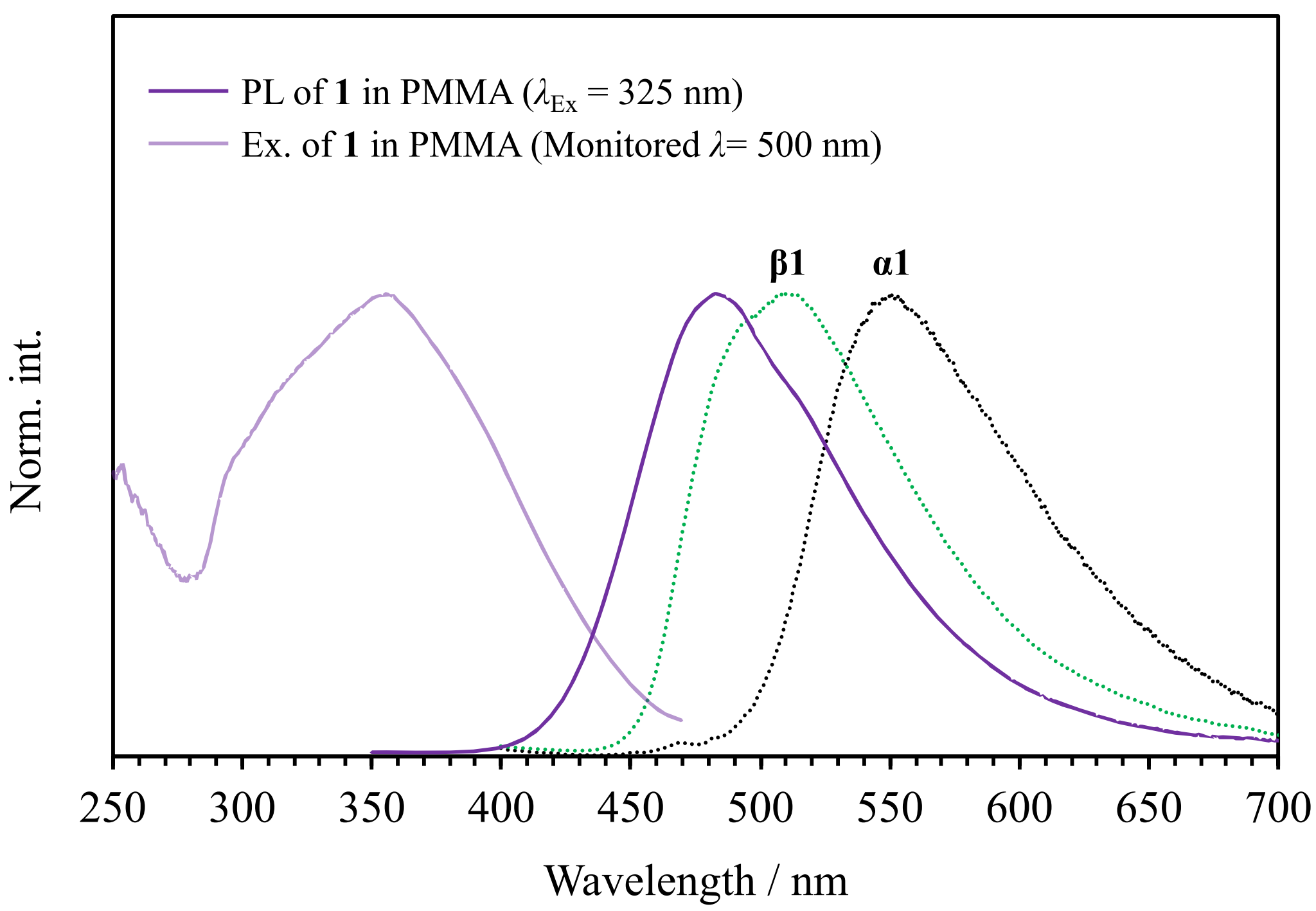

Figure S14. Photoluminescence (PL) and excitation (Ex.) spectra of **1** in polymethyl methacrylate (PMMA).

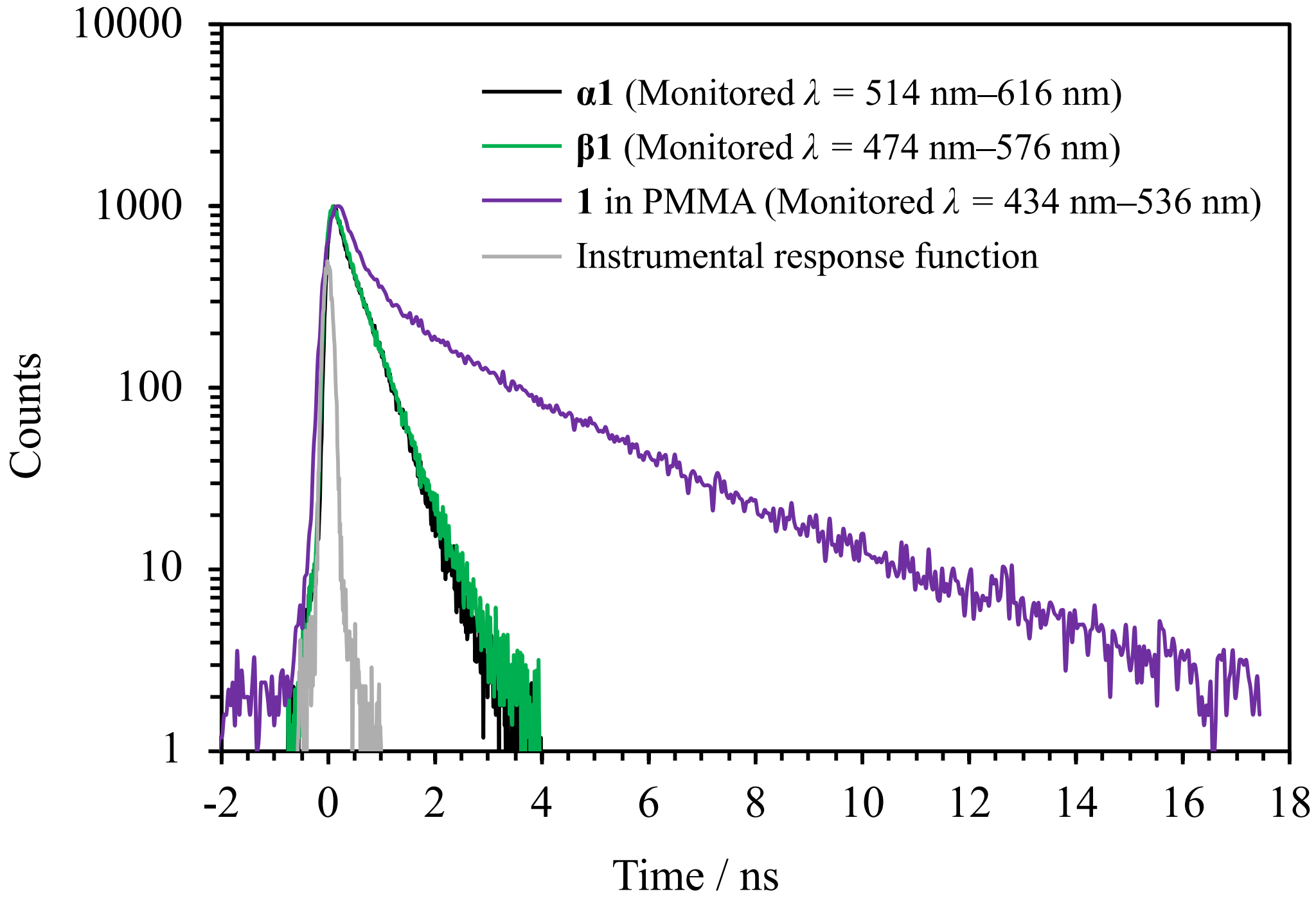

Figure S15. PL decay profiles measured for **α1**, **β1** and **1** in PMMA film.

Table S1. Fluorescence Lifetime

	A_1	τ_1 / ns	A_2	τ_2 / ns	A_3	τ_3 / ns
**α1**	0.50	0.07	0.06	0.50	–	–
**β1**	0.54	0.17	0.03	0.54	–	–
**1** in PMMA	0.15	0.22	0.04	1.07	0.02	3.33

The decay profile using the fitting function: $f(t) = A_1\exp(-t/\tau_1)+A_2\exp(-t/\tau_2)+A_3\exp(-t/\tau_3)$ according to a constant (A), and decay time (t) revealed that the luminescence lifetime could be decomposed into two or three parts.

Table S2. Radiative and Nonradiative Rate Constants

	τ_{ave} / ns	Φ_{PL}	k_r / 10^9 s^{-1}	k_{nr} / 10^9 s^{-1}
**α1**	0.27	0.054	0.20	3.52
**β1**	0.23	0.131	0.58	3.85
**1** in PMMA	1.93	0.024	0.01	0.51

Table S3. Crystallographic Data for **α1**

	**α1**
CCDC No.	2523856
Empirical formula	$C_{30}H_{26}Br_2N_2O_6$
Formula weight	670.35
Temperature [K]	113
Radiation [Å]	0.71073 (MoKα)
Crystal system	triclinic
Space group	*P*-1
a [Å]	4.0411(3)
b [Å]	8.0465(6)
c [Å]	21.2304(13)
α [°]	87.447(6)
β [°]	85.175(5)
γ [°]	81.129(6)
Volume [$Å^3$]	679.32(8)
Z	1
Z'	0.5
Density (calculated) [g/cm^3]	1.639
Absorption coefficient [mm^{-1}]	3.032
F(000)	338.0
2*θ* range [°]	3.852 to 52.746
Reflections collected	7619
Independent reflections	2740 [R_{int} = 0.0695]
Data / restraints / parameters	2740 / 0 / 184
Goodness-of-fit on F^2	1.099
R_1[a] [I≧2σ(I)]	0.0764
wR_2[b] (all data)	0.1974
Largest diff. peak / hole [e.$Å^{-3}$]	2.66 / −1.12

a: $R_1 = (\sum||F_o|-|F_c||)/(\sum|F_o|)$. b: $wR_2 = [\sum w(F_o^2-F_c^2)^2/\sum wF_o^4]^{1/2}$

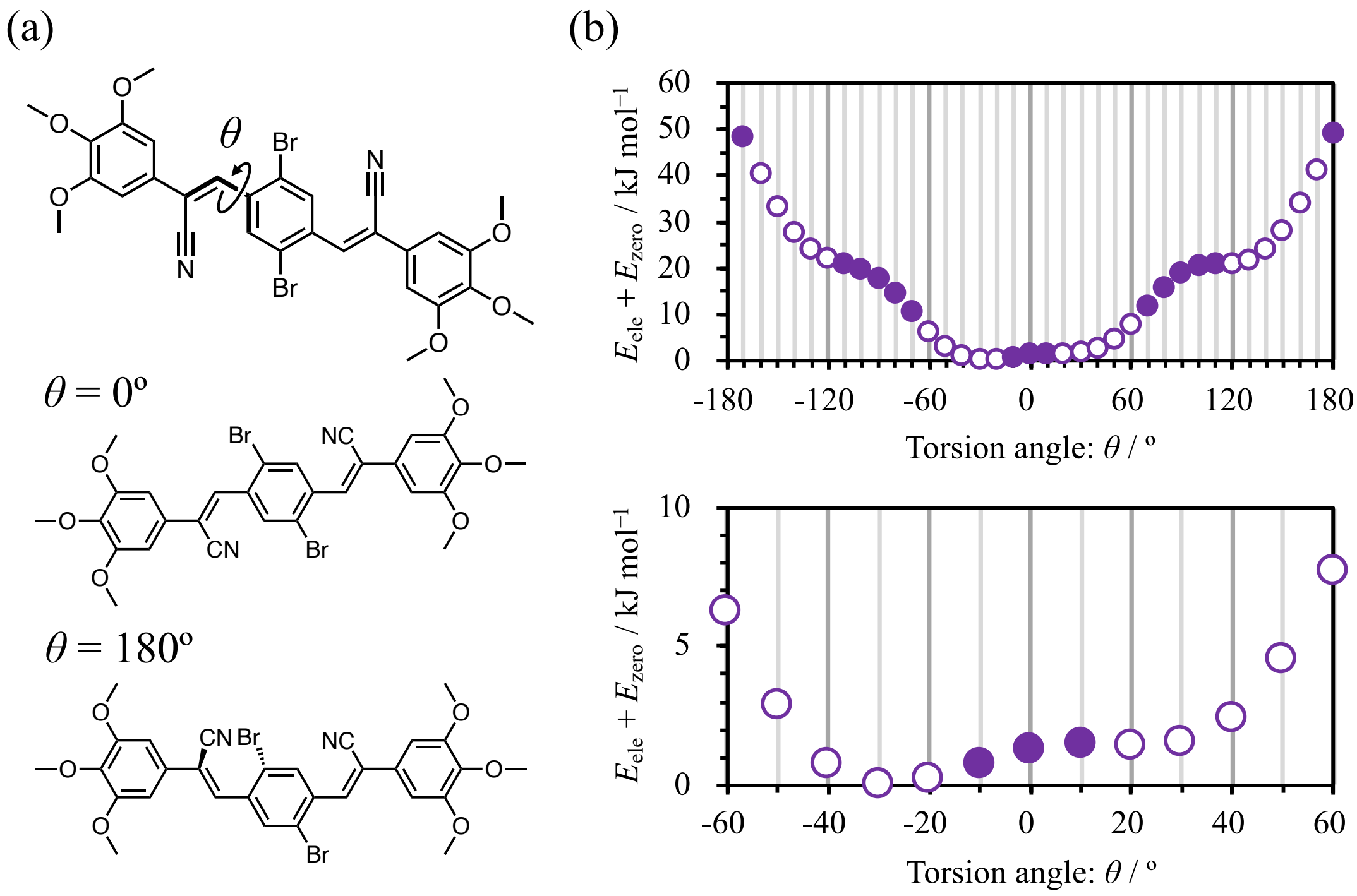

Figure S16. (a) The torsion angle (θ) is frozen at **1** within the seating analysis. For example, structures with $\theta = 0$º and $\theta = 180$º. (b) Relative energy plot calculated from total electron energy and zero-point energy. using DFT calculation. Hollow point: No imaginary frequency, Solid point: Possesses one imaginary frequency.

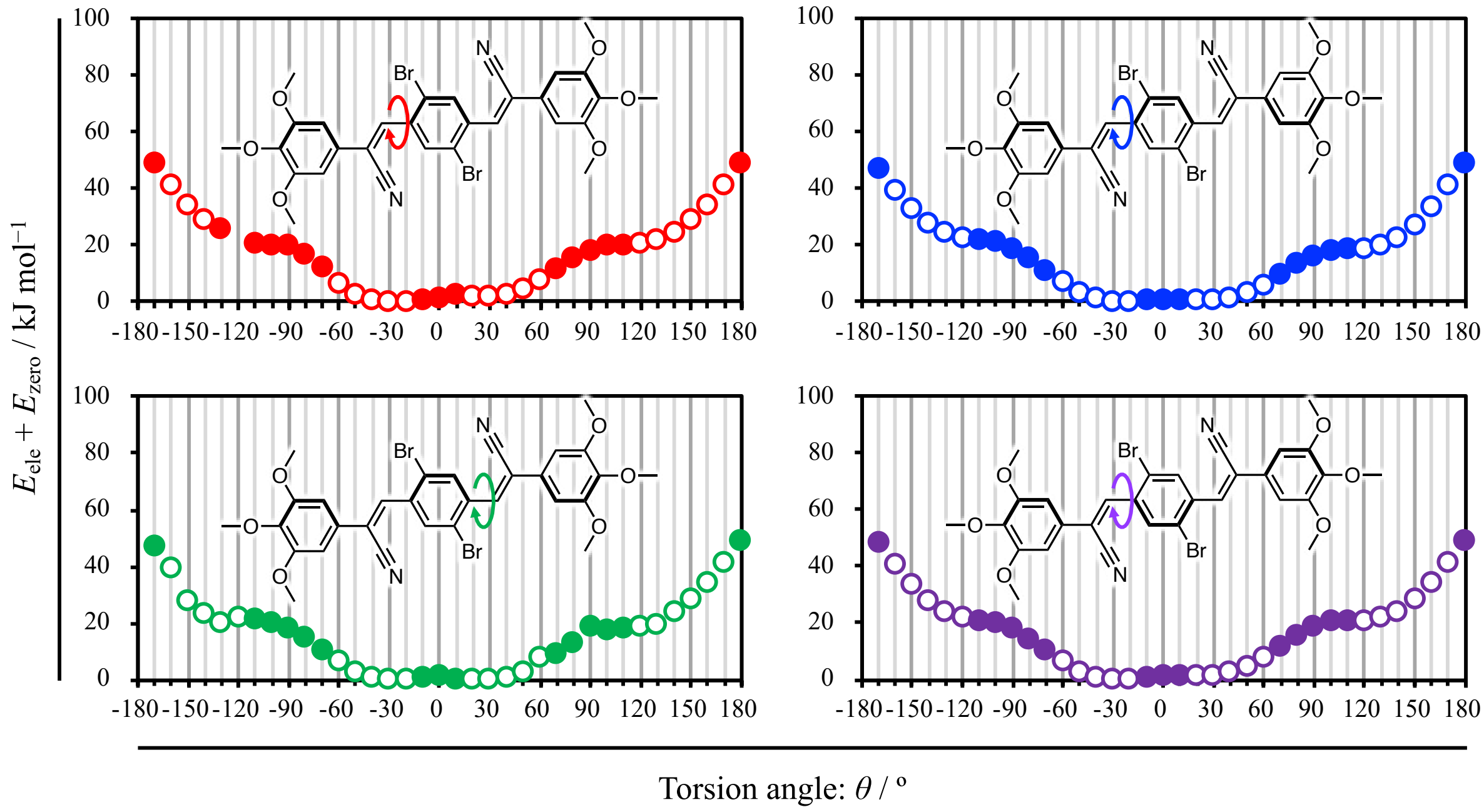

Figure S17. Conformation analysis corresponding to the initial structure of **1**. Hollow point: No imaginary frequency, Solid point: Possesses one imaginary frequency.

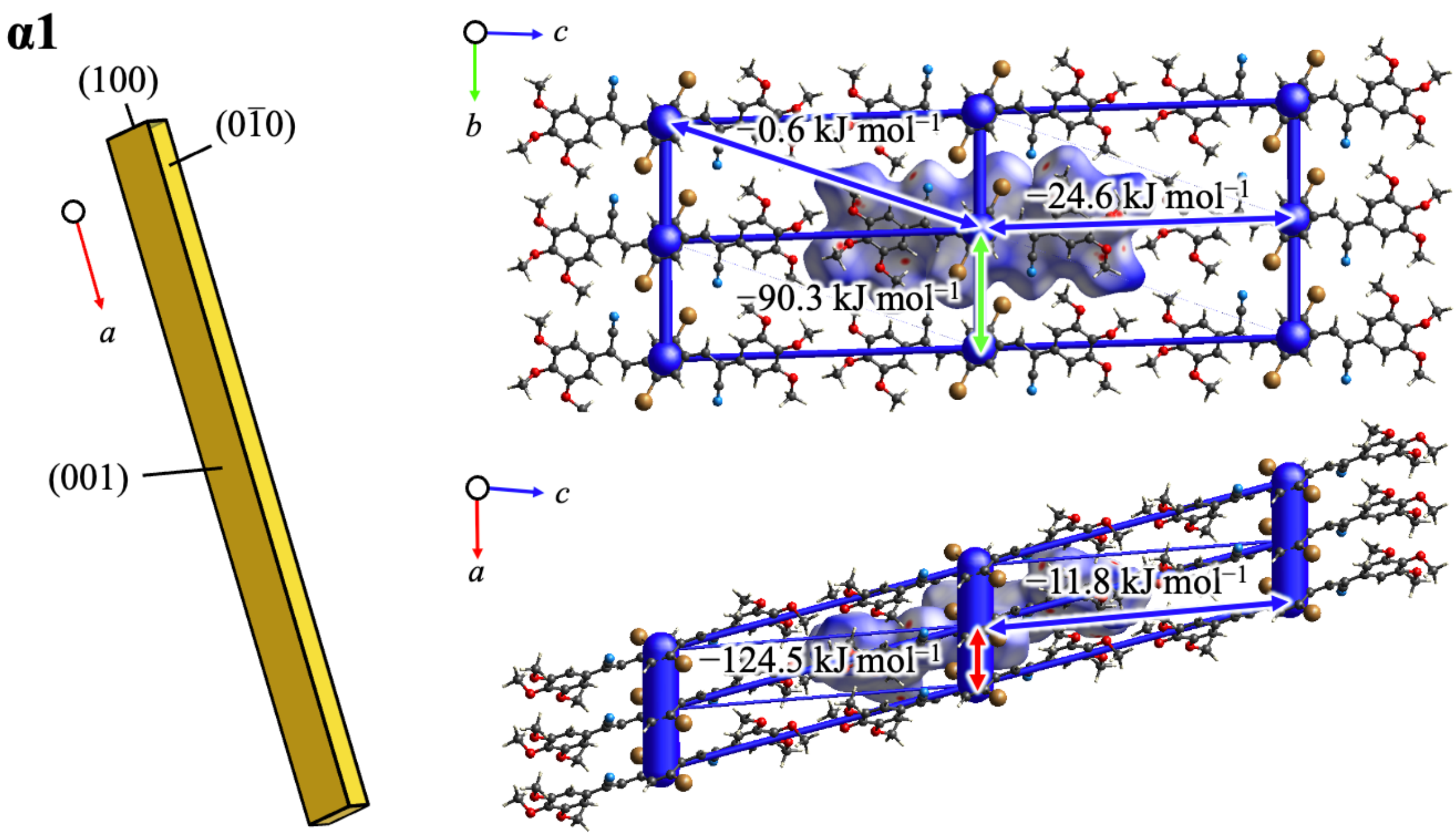

Figure S18. Relation between the structure of crystal **α1** and interaction energy (blue column width indicates energy).

Table S4. Energy Calculation Results for the Framework of **α1**

Symmetry operation	Distance[a] / Å	Electron density	Interaction energy[b] / kJ mol^{-1}				
			E_{ele}	E_{pol}	E_{dis}	E_{rep}	E_{tot}
x, y, z	9.54	HF / 3–21G	−17.2	−6.7	−24.7	13.1	−33.4
x, y, z	21.94	HF / 3–21G	−12.4	−5.9	−17.4	9.3	−24.6
x, y, z	4.04	HF / 3–21G	−20.5	− 19.9	−199.9	110.3	−124.5
x, y, z	8.05	HF / 3–21G	−16.4	−6.2	−53.4	30.3	−44.3
x, y, z	8.43	HF / 3–21G	−1.1	−0.9	−19.1	4.9	−12.6
x, y, z	22.37	HF / 3–21G	−0.8	−0.2	−1.5	0.1	−0.6
x, y, z	21.23	HF / 3–21G	−2.2	−0.8	−15.8	6.4	−11.8

[a] Distance is length between molecular centroids (mean atomic position). [b] E_{ele}: Electrostatic energy, E_{pol}: Polarization energy, E_{dis}: Dispersion energy, E_{rep}: Exchange-repulsion energy, E_{tot}: Total interaction energy

Table S5. Crystallographic Data for **β1**

	**β1**
CCDC No.	2523857
Empirical formula	$C_{30}H_{26}Br_2N_2O_6$
Formula weight	670.35
Temperature [K]	113
Radiation [Å]	0.71073 (MoKα)
Crystal system	monoclinic
Space group	$P2_1/c$
a [Å]	8.2297(3)
b [Å]	12.2974(4)
c [Å]	14.0528(6)
α [°]	90
β [°]	102.229(4)
γ [°]	90
Volume [$Å^3$]	1389.93(9)
Z	2
Z'	0.5
Density (calculated) [g/cm^3]	1.602
Absorption coefficient [mm^{-1}]	2.964
$F(000)$	676.0
2θ range [°]	4.446 to 52.728
Reflections collected	8997
Independent reflections	2842 [R_{int} = 0.0291]
Data / restraints / parameters	2842 / 0 / 184
Goodness-of-fit on F^2	1.055
R_1[a] [I≧2σ(I)]	0.0336
wR_2[b] (all data)	0.0880
Largest diff. peak / hole [e.$Å^{-3}$]	0.62 / −0.32

a: $R_1 = (\sum||F_o|-|F_c||)/(\sum|F_o|)$. b: $wR_2 = [\sum w(F_o^2-F_c^2)^2/\sum wF_o^4]^{1/2}$

β1

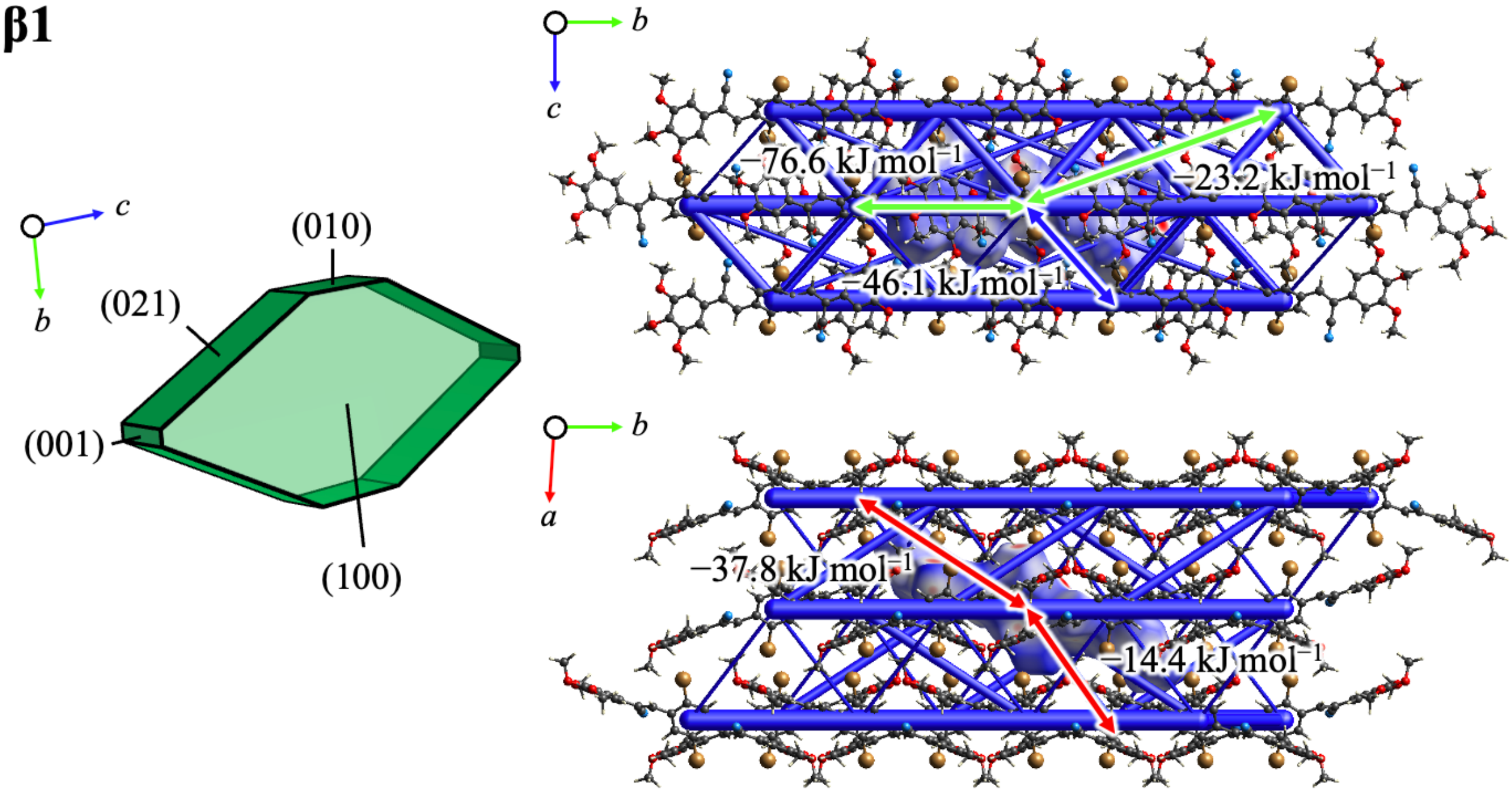

Figure S19. Relation between the structure of **β1** and interaction energy (blue column width indicates energy).

Table S6. Energy Calculation Results for the Framework of **β1**

Symmetry operation	Distance[a] / Å	Electron density	Interaction energy[b] / kJ mol^{-1}				
			E_{ele}	E_{pol}	E_{dis}	E_{rep}	E_{tot}
− x, y+1/2, −z+1/2	14.80	HF / 3–21G	−4.2	−1.8	−55.0	21.2	−37.8
x, y, z	11.42	HF / 3–21G	−6.3	−1.7	−24.2	18.4	−14.4
x, y, z	19.74	HF / 3–21G	−14.2	−4.2	−11.7	5.7	−23.2
− x, y+1/2, −z+1/2	12.30	HF / 3–21G	−31.0	−17.2	−93.6	61.5	−77.2
x, y, z	9.43	HF / 3–21G	−22.1	−6.8	−43.5	24.8	−46.1

[a] Distance is length between molecular centroids (mean atomic position). [b] E_{ele}: Electrostatic energy, E_{pol}: Polarization energy, E_{dis}: Dispersion energy, E_{rep}: Exchange-repulsion energy, E_{tot}: Total interaction energy

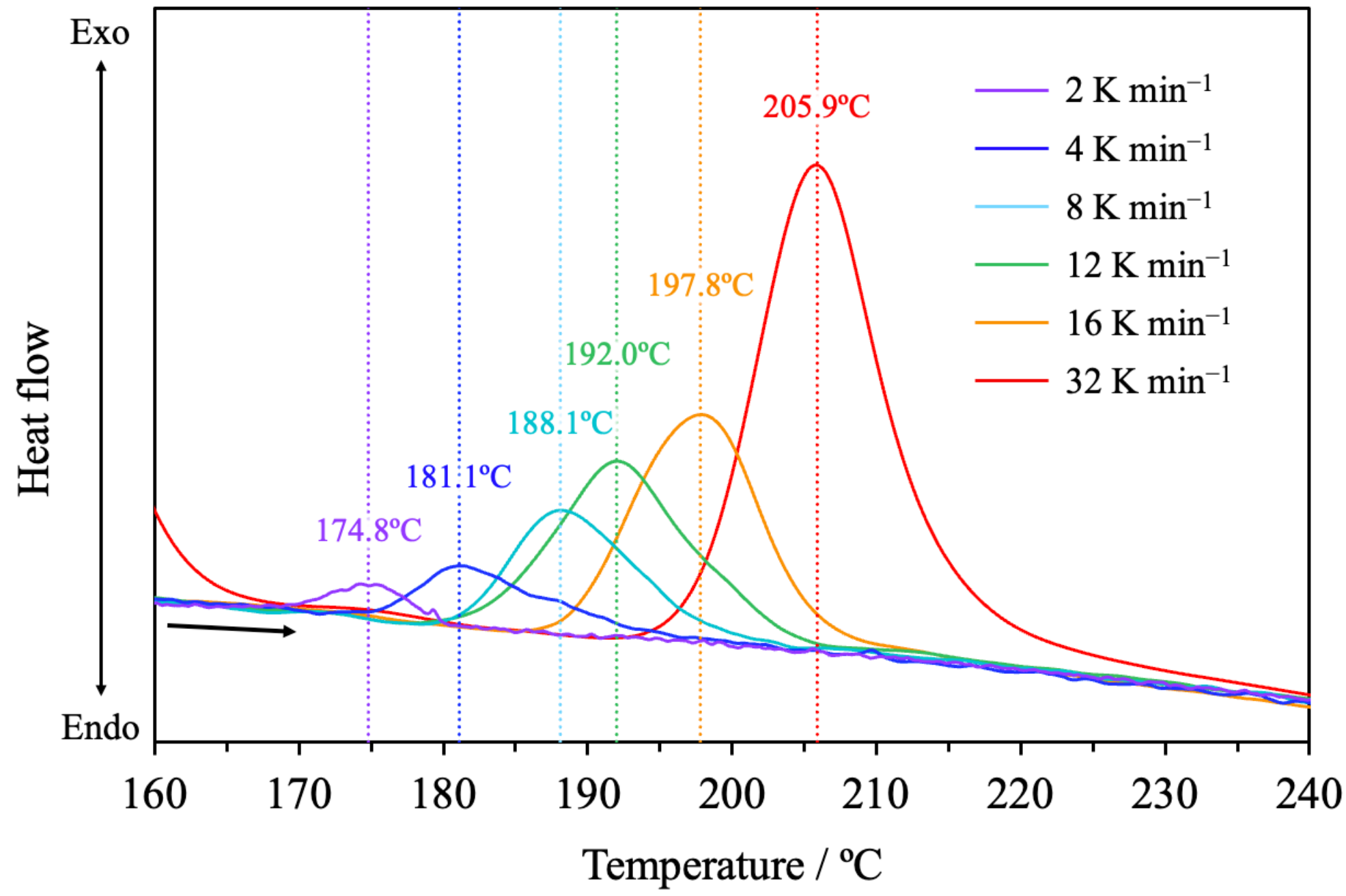

Figure S20. DSC trace of **α1** under different heating rate.

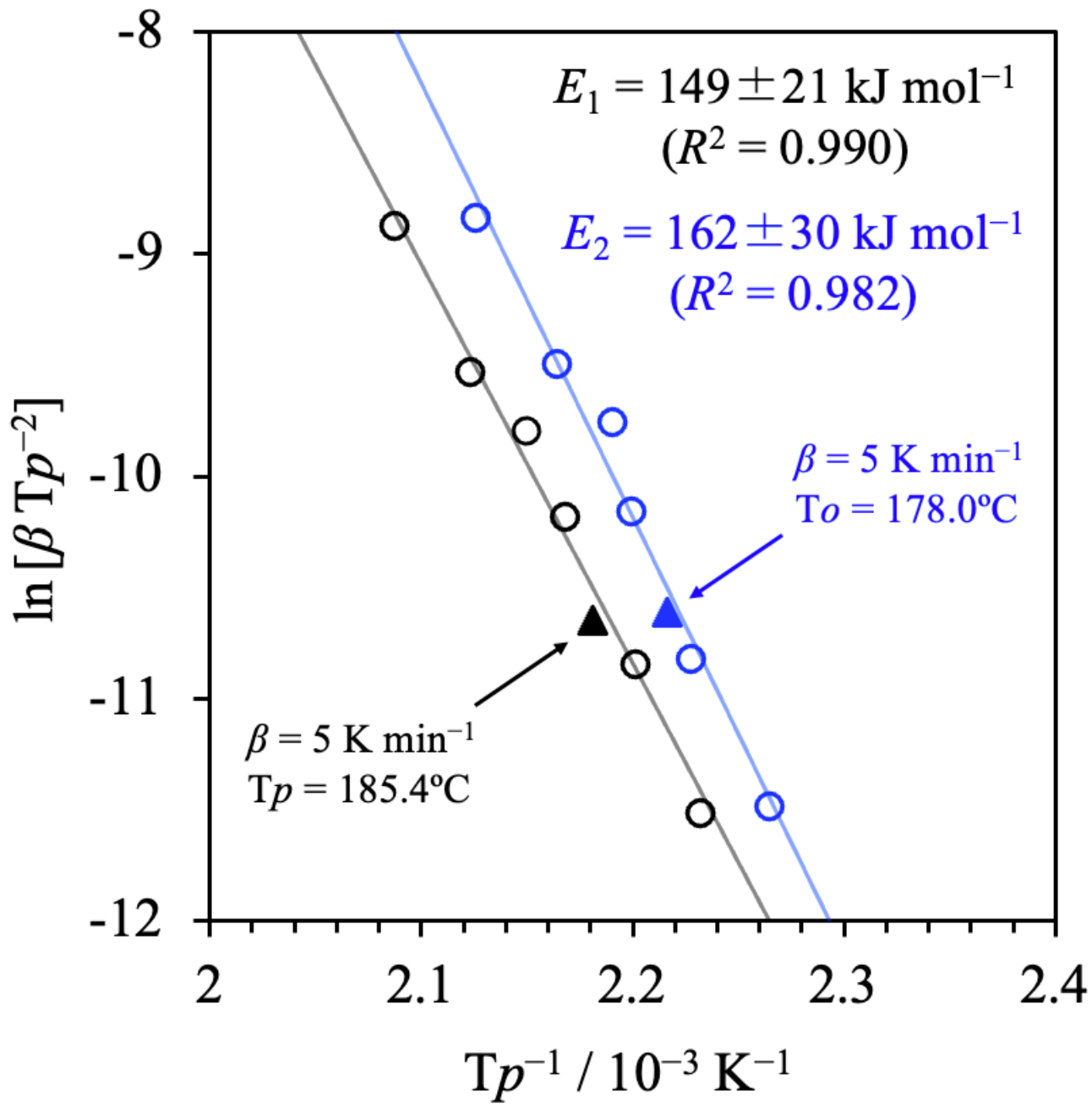

Figure S21. Linear regression of the Kissinger plot gave a slope of $-(1.80\pm0.09)\times10^4$ K, corresponding to an apparent activation energy of 149±21 kJ mol^{-1} (95% confidence interval, n = 6).

$$\ln\left(\frac{\beta}{T_p^2}\right) = -\frac{E_a}{R}\frac{1}{T_p} + \text{const.}$$

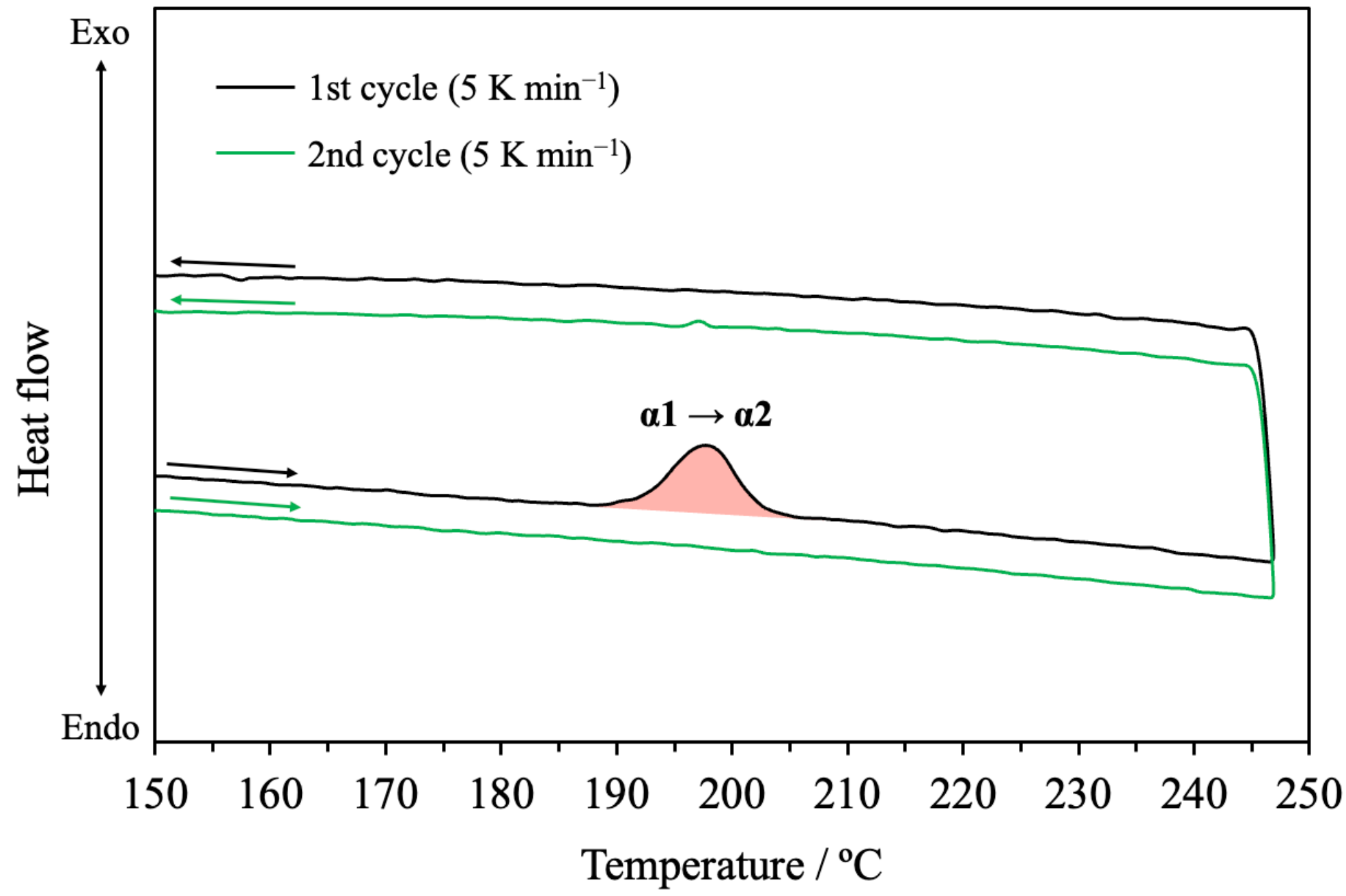

Figure S22. Cyclic DSC trace of **α1**.

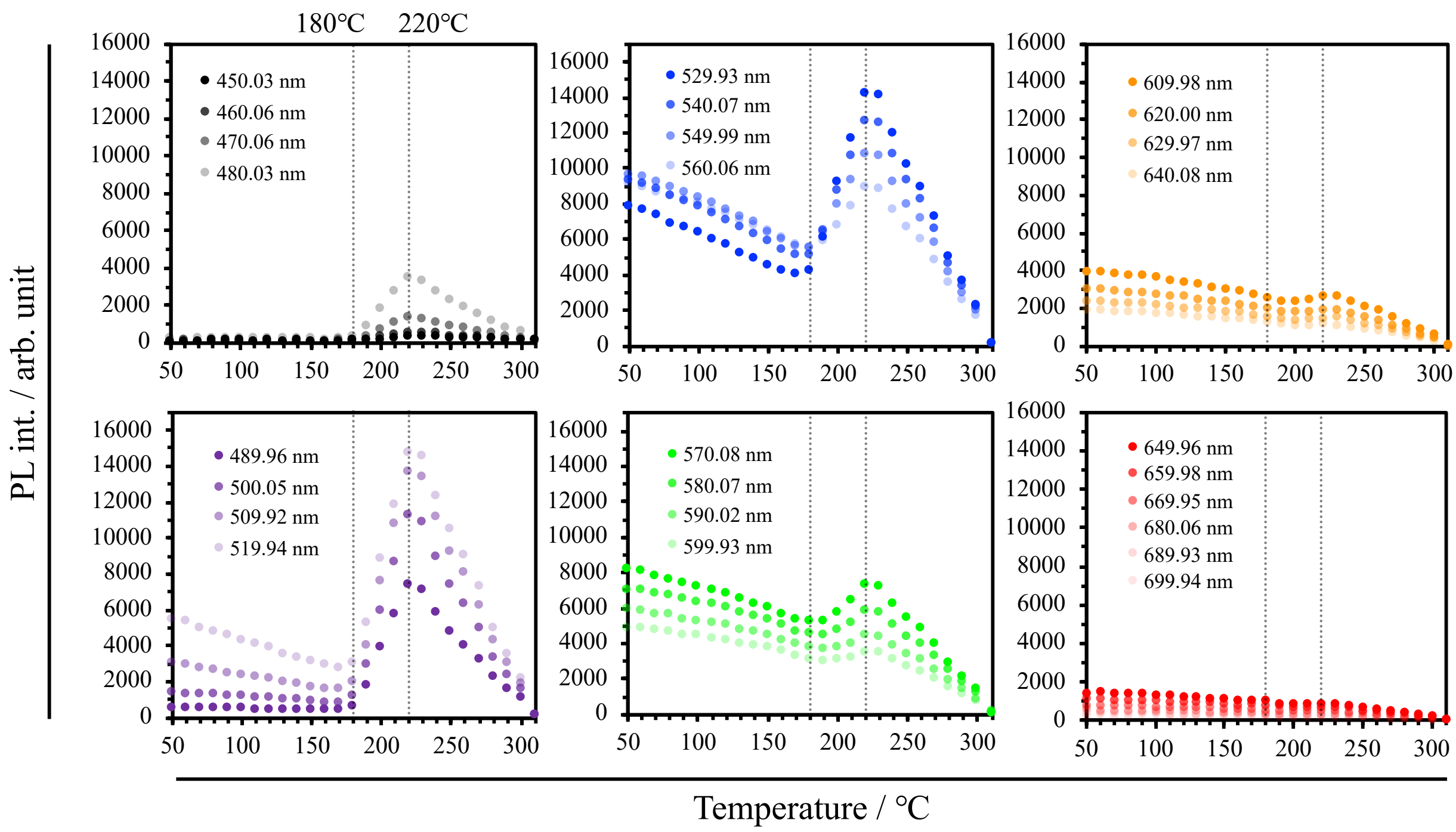

Figure S23. Monitoring of luminescence intensity at each PL wavelength in temperature-dependent PL spectra.

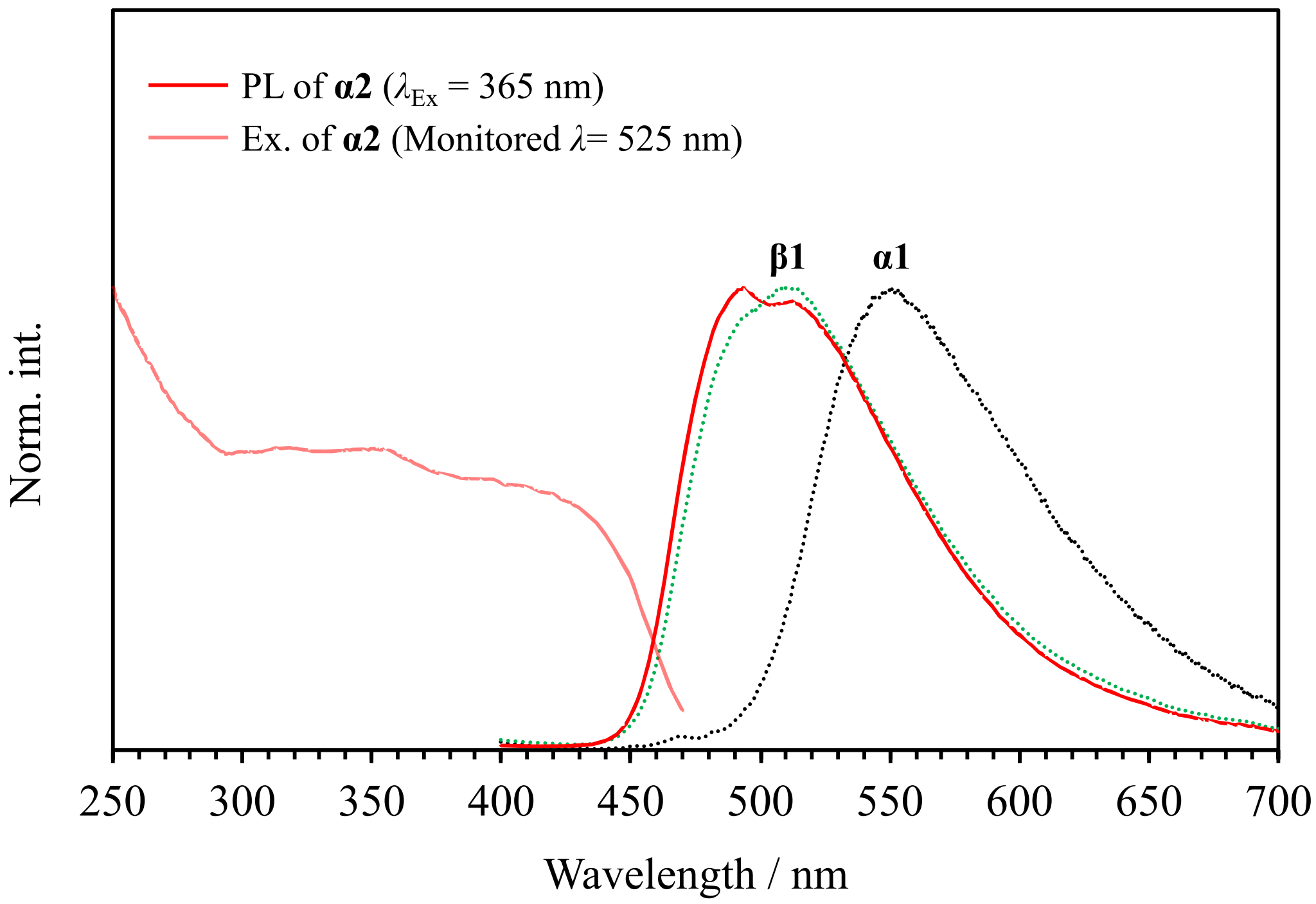

Figure S24. Photoluminescence (PL) and excitation (Ex.) spectra of **α2**.

Table S7. Crystallographic Data for Crystal **α2**

	**α2**
CCDC No.	–
Empirical formula	$C_{30}H_{26}Br_2N_2O_6$
Formula weight	670.35
Temperature [K]	113
Radiation [Å]	0.71073 (MoKα)
Crystal system	monoclinic
Space group	$P2_1/c$
a [Å]	8.2409(4)
b [Å]	12.2912(5)
c [Å]	14.0312Z(7)
α [°]	90
β [°]	102.515(4)
γ [°]	90
Volume [$Å^3$]	1387.46(11)
Z	2
Z'	0.5
Density (calculated) [g/cm^3]	1.605
Absorption coefficient [mm^{-1}]	2.969
F(000)	676.0
2θ range [°]	4.452 to 52.732
Reflections collected	11799
Independent reflections	2836 [R_{int} = 0.0330]
Data / restraints / parameters	2836 / 0 / 184
Goodness-of-fit on F^2	1.016
R_1[a] [I≧2σ(I)]	0.0285
wR_2[b] (all data)	0.671
Largest diff. peak / hole [e.$Å^{-3}$]	0.49 / −0.29

a: $R_1 = (\sum||F_o|-|F_c||)/(\sum|F_o|)$, b: $wR_2 = [\sum w(F_o^2-F_c^2)^2/\sum wF_o^4]^{1/2}$

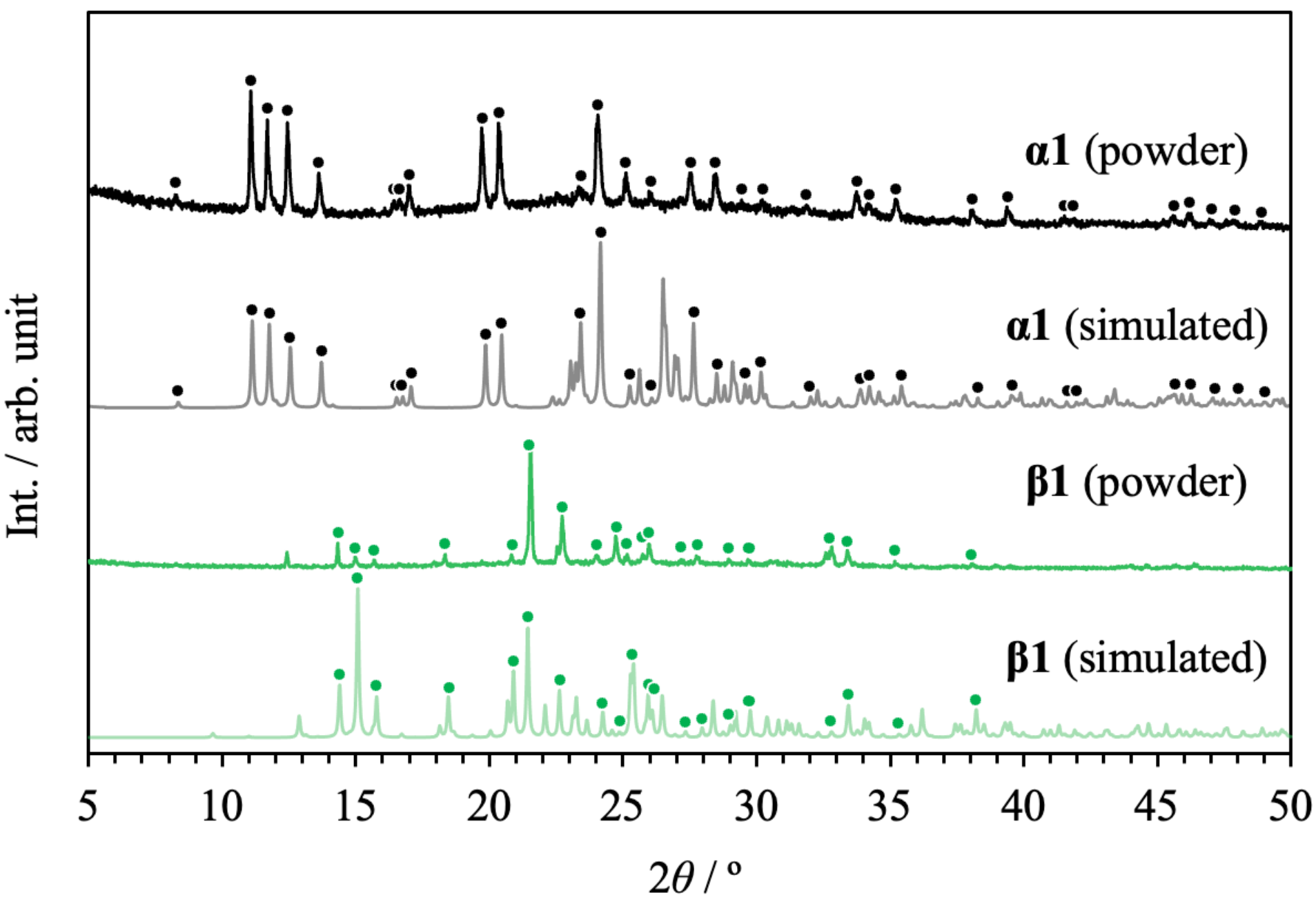

Figure S25. Powder X-ray diffraction and predicted patterns of **α1** and **β1**.

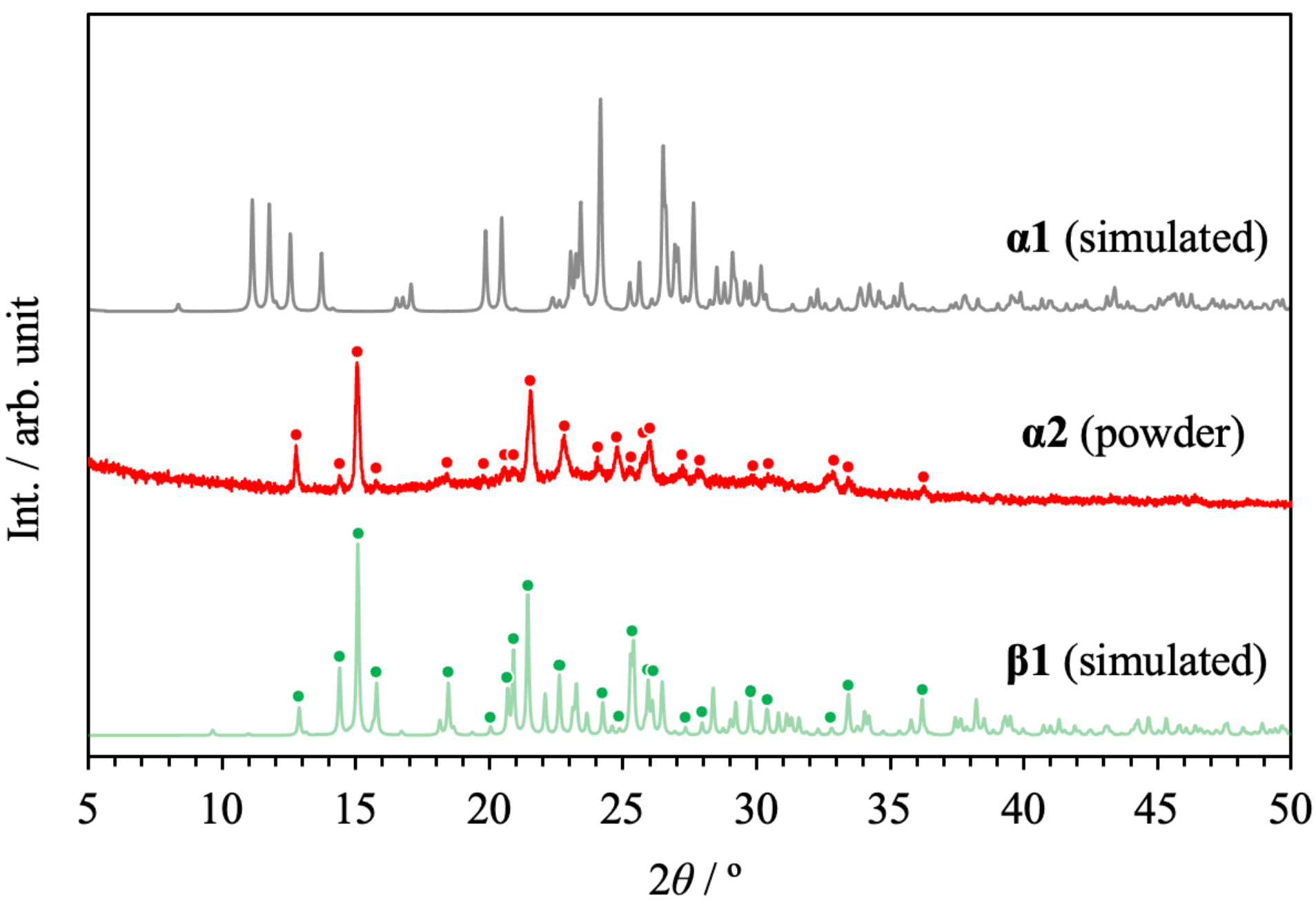

Figure S26. Powder X-ray diffraction of **α2**.

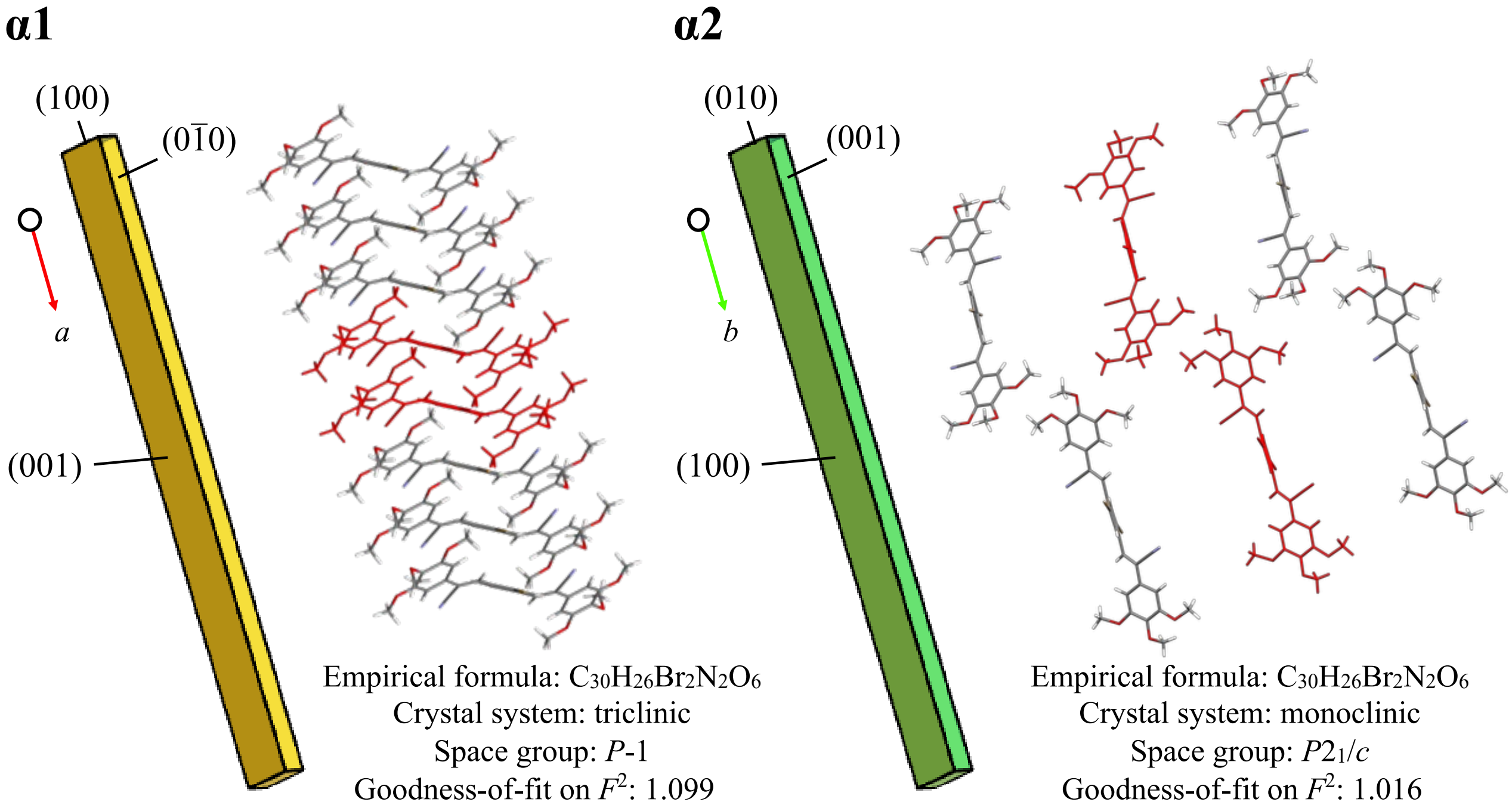

Figure S27. Crystal structure of **α1** and **α2**.

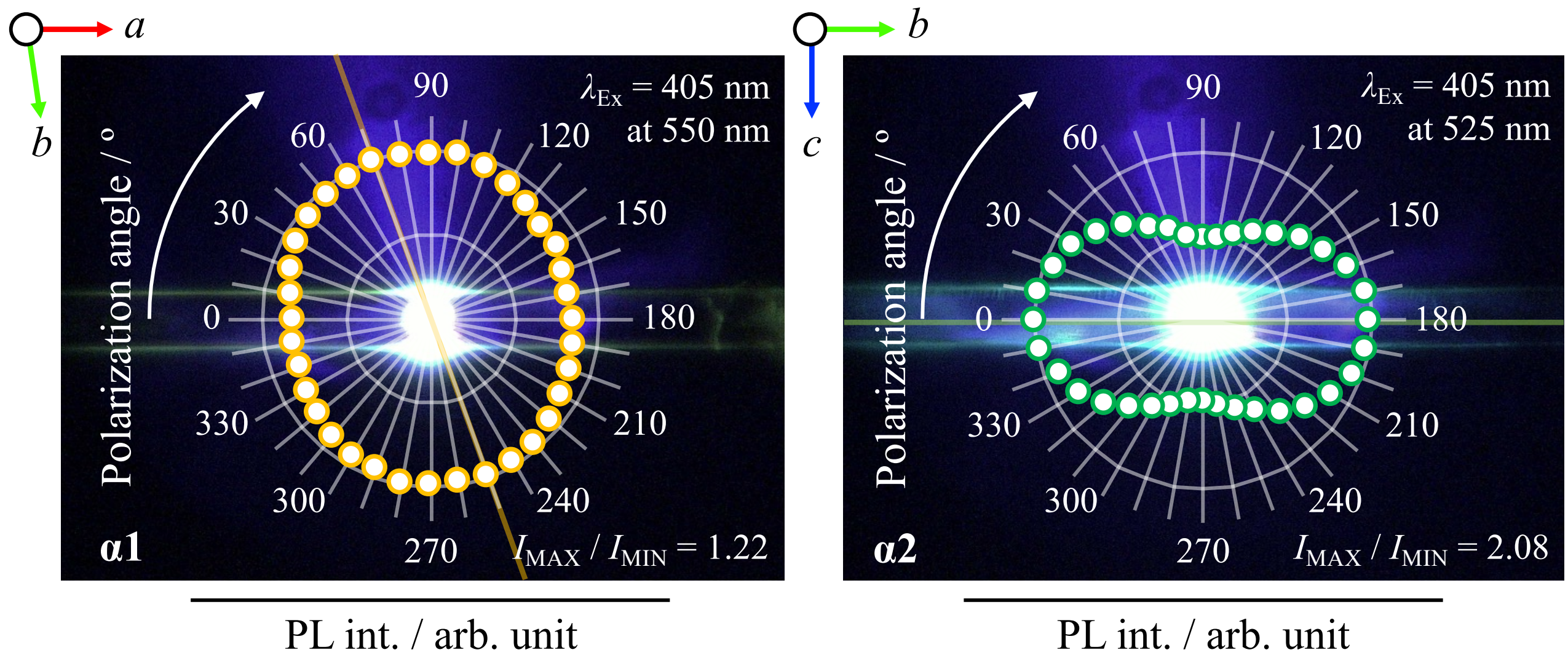

Figure S28. Incident polarization angle-dependent PL intensities for each face of **α1** and **α2**.

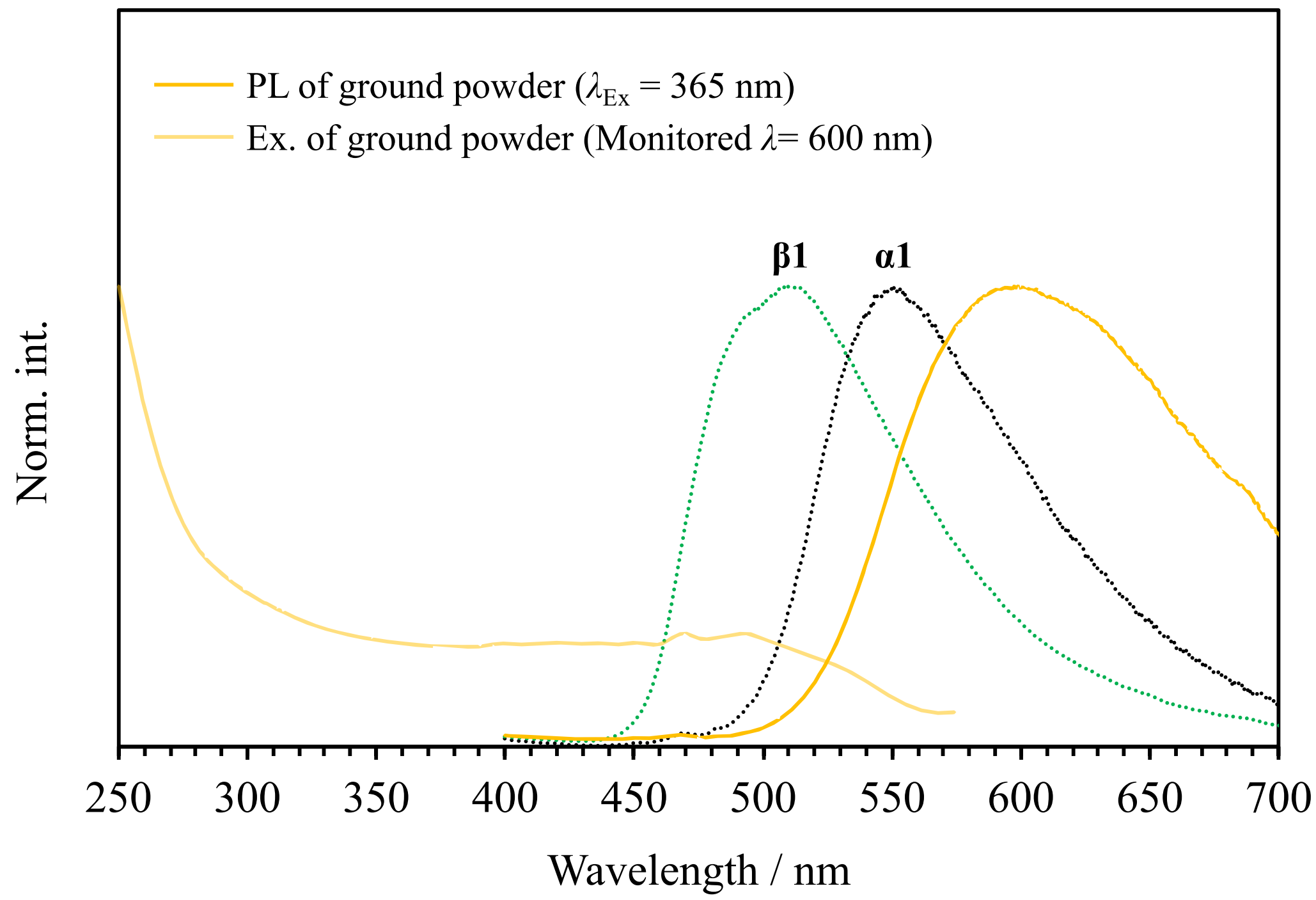

Figure S29. Photoluminescence (PL) and excitation (Ex.) spectra of powder obtained by grinding **α1**.

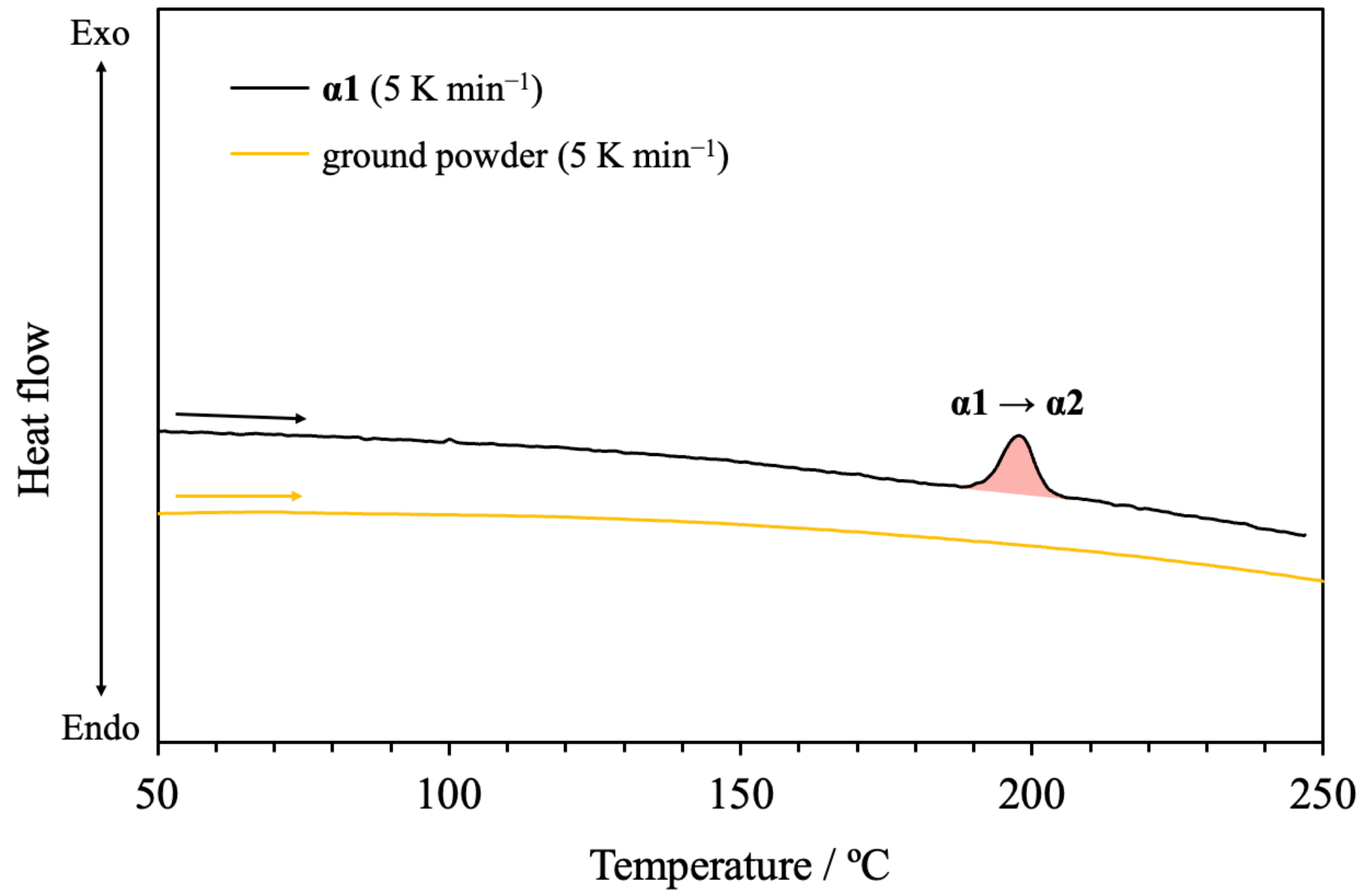

Figure S30. DSC trace of **α1** and ground powder.

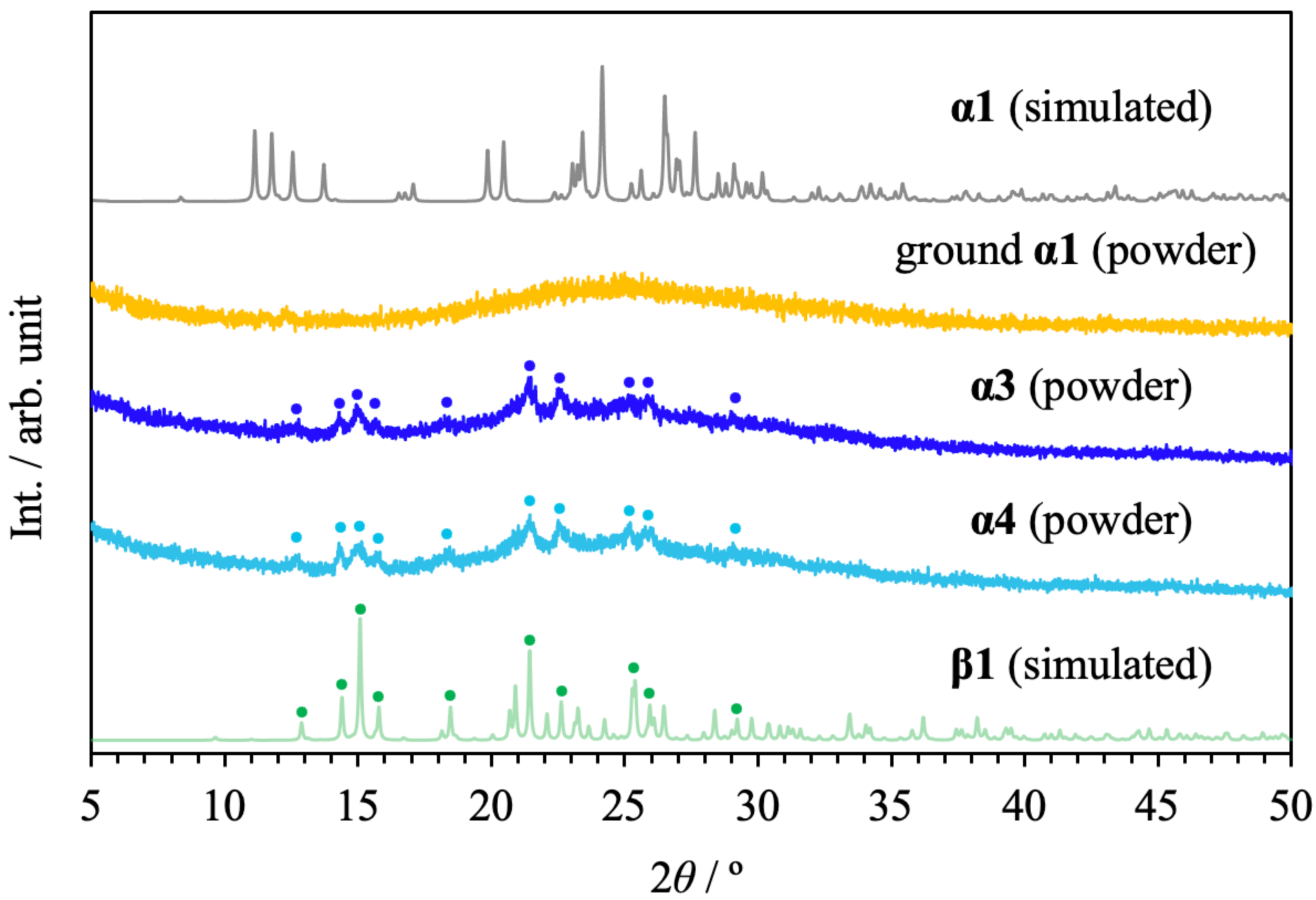

Figure S31. Powder X-ray diffraction patterns of ground **α1**, **α3** and **α4**.

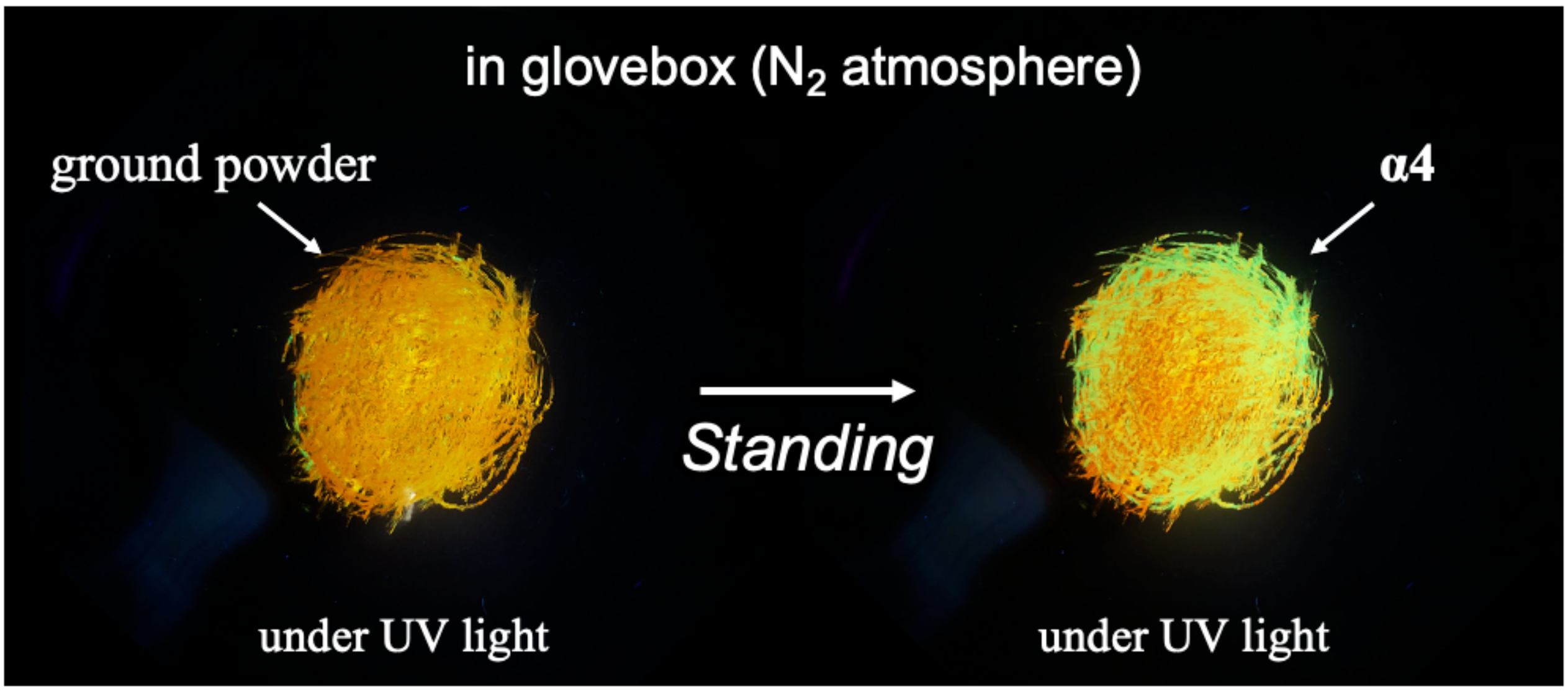

Figure S32. Fluorescent images of powders in glovebox.

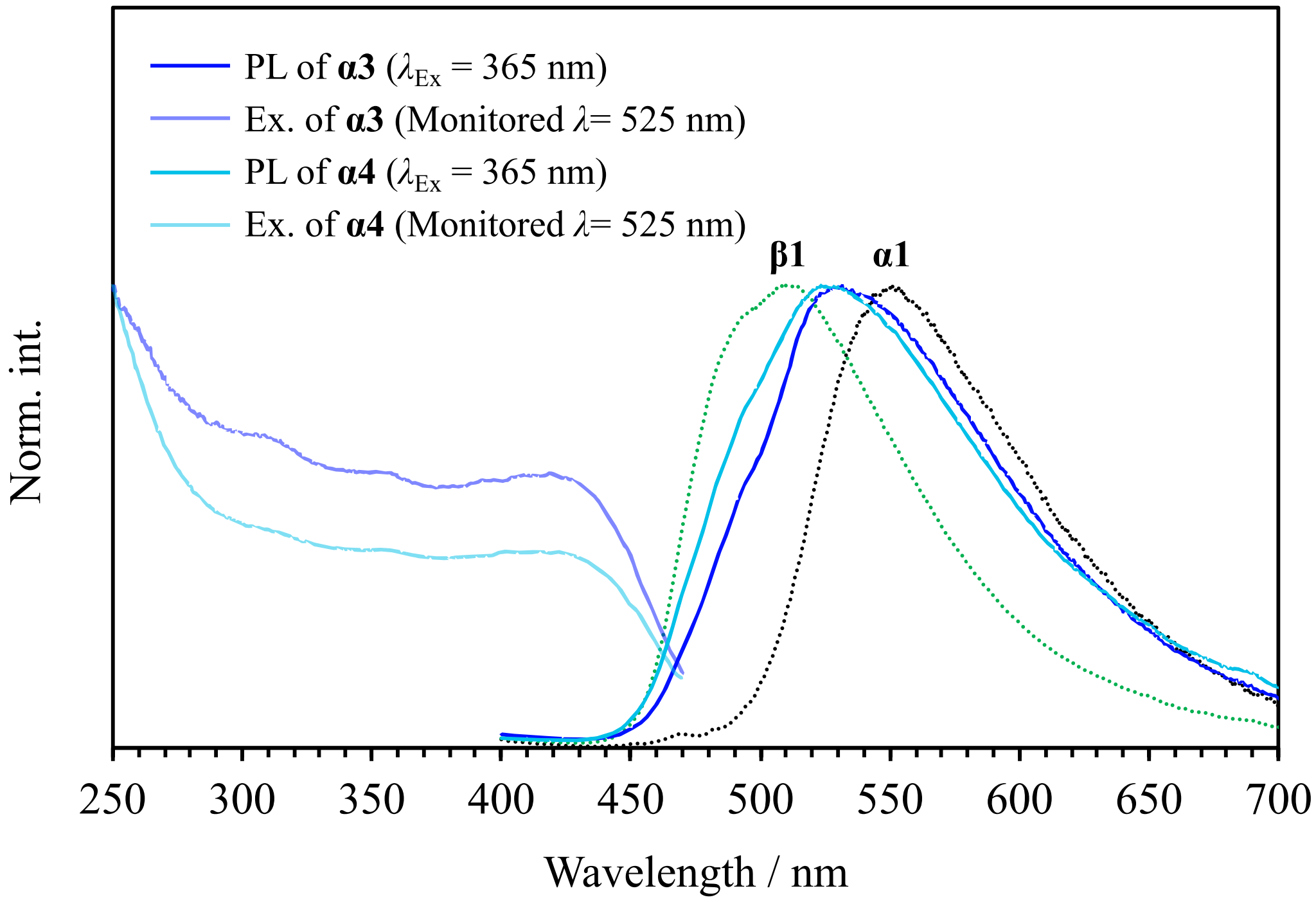

Figure S33. Photoluminescence (PL) and excitation (Ex.) spectra of **α3** and **α4**.

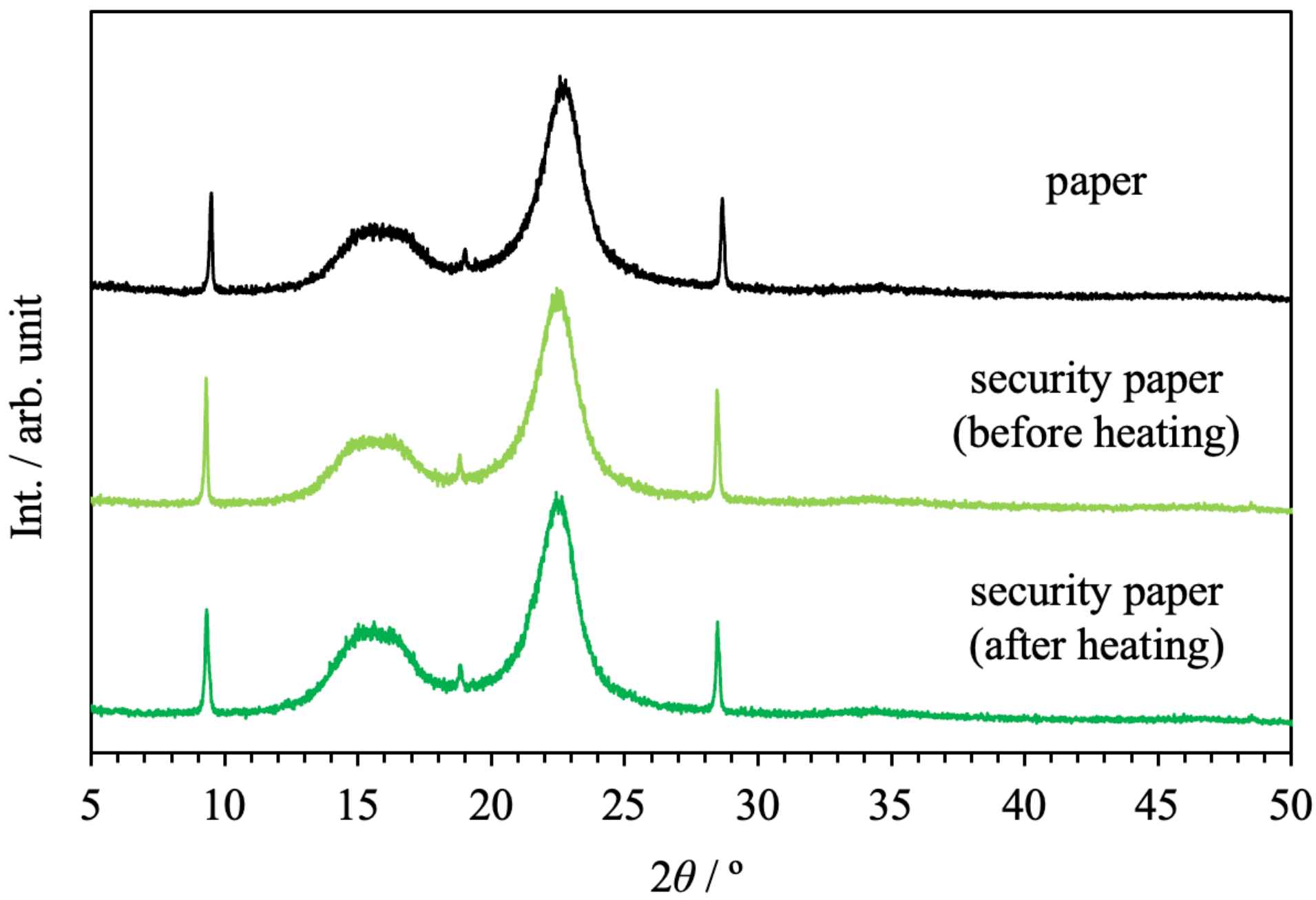

Figure S34. Powder X-ray diffraction patterns of papers.

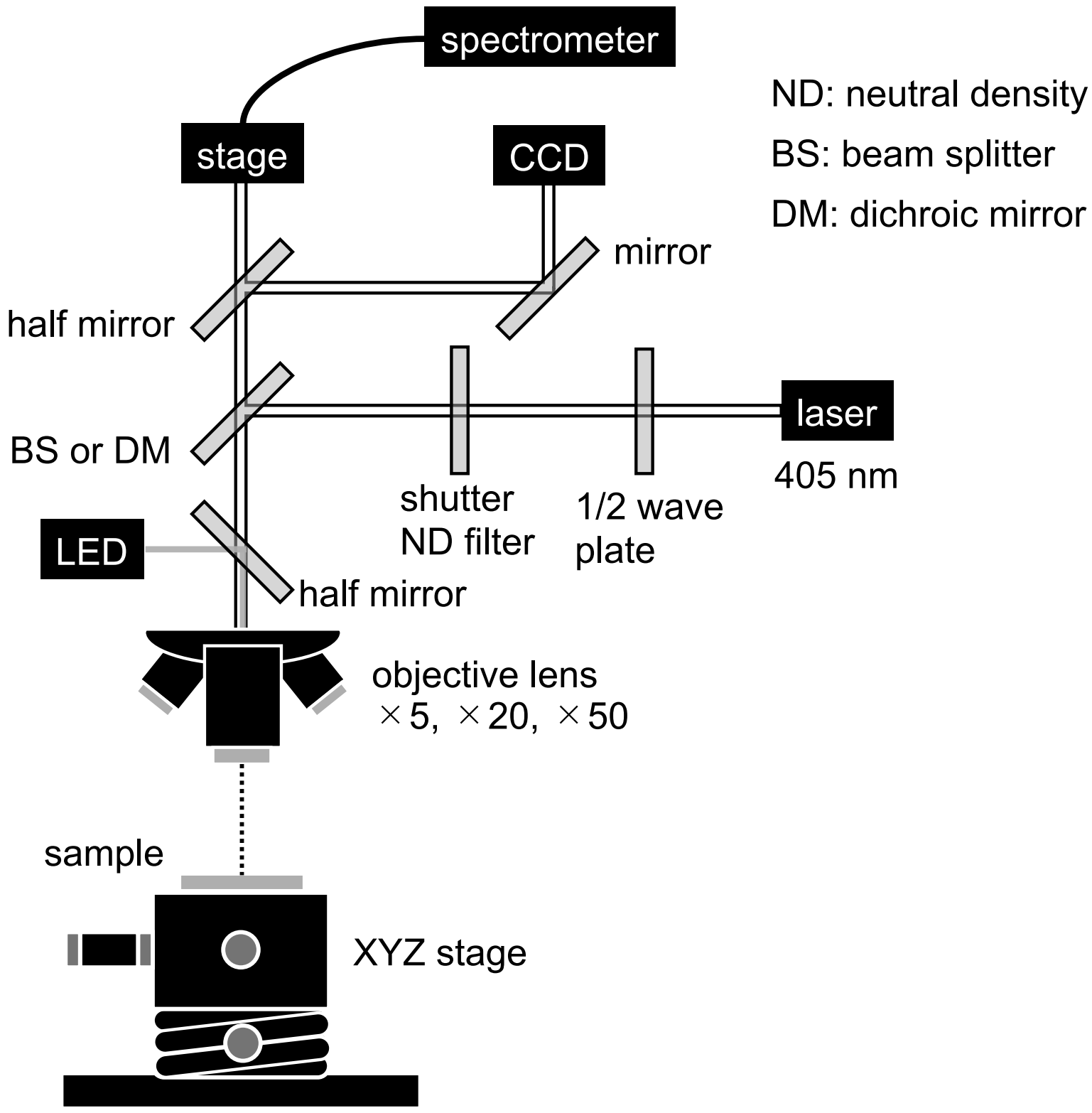

Figure S35. Measurement setup for polarization dependence of the PL intensity.

Alert level C

DIFMX02_ALERT_1_C The maximum difference density is > 0.1*ZMAX*0.75
The relevant atom site should be identified.
PLAT094_ALERT_2_C Ratio of Maximum / Minimum Residual Density .... 2.37 Report
PLAT097_ALERT_2_C Large Reported Max. (Positive) Residual Density 2.66 eA-3
PLAT341_ALERT_3_C Low Bond Precision on C-C Bonds .... 0.00915 Ang.

Alert level G

PLAT012_ALERT_1_G No _shelx_res_checksum Found in CIF ...... Please Check
PLAT072_ALERT_2_G SHELXL First Parameter in WGHT Unusually Large 0.11 Report
PLAT720_ALERT_4_G Number of Unusual/Non-Standard Labels .... 32 Note
Br01 O002 O003 C005 C006 N007 C008 H008
C009 H009 C00A C00B C00C C00D H00D C00E
H00E C00F C00G C00H C00I H00A H00B H00C
C00J H00F H00G H00H C00K H00I H00J H00K
PLAT933_ALERT_2_G Number of HKL-OMIT Records in Embedded .res File 1 Note
-1 0 1,
PLAT941_ALERT_3_G Average HKL Measurement Multiplicity .... 2.8 Low

0 **ALERT level A** = Most likely a serious problem - resolve or explain
0 **ALERT level B** = A potentially serious problem, consider carefully
4 **ALERT level C** = Check. Ensure it is not caused by an omission or oversight
5 **ALERT level G** = General information/check it is not something unexpected

2 ALERT type 1 CIF construction/syntax error, inconsistent or missing data
4 ALERT type 2 Indicator that the structure model may be wrong or deficient
2 ALERT type 3 Indicator that the structure quality may be low
1 ALERT type 4 Improvement, methodology, query or suggestion
0 ALERT type 5 Informative message, check

It is advisable to attempt to resolve as many as possible of the alerts in all categories. Often the minor alerts point to easily fixed oversights, errors and omissions in your CIF or refinement strategy, so attention to these fine details can be worthwhile. It is up to the individual to critically assess their own results and, if necessary, seek expert advice.

PLATON version of 26/09/2025; check.def file version of 20/09/2025

duplicate check

No duplication found

Datablock exp_1092 - ellipsoid plot

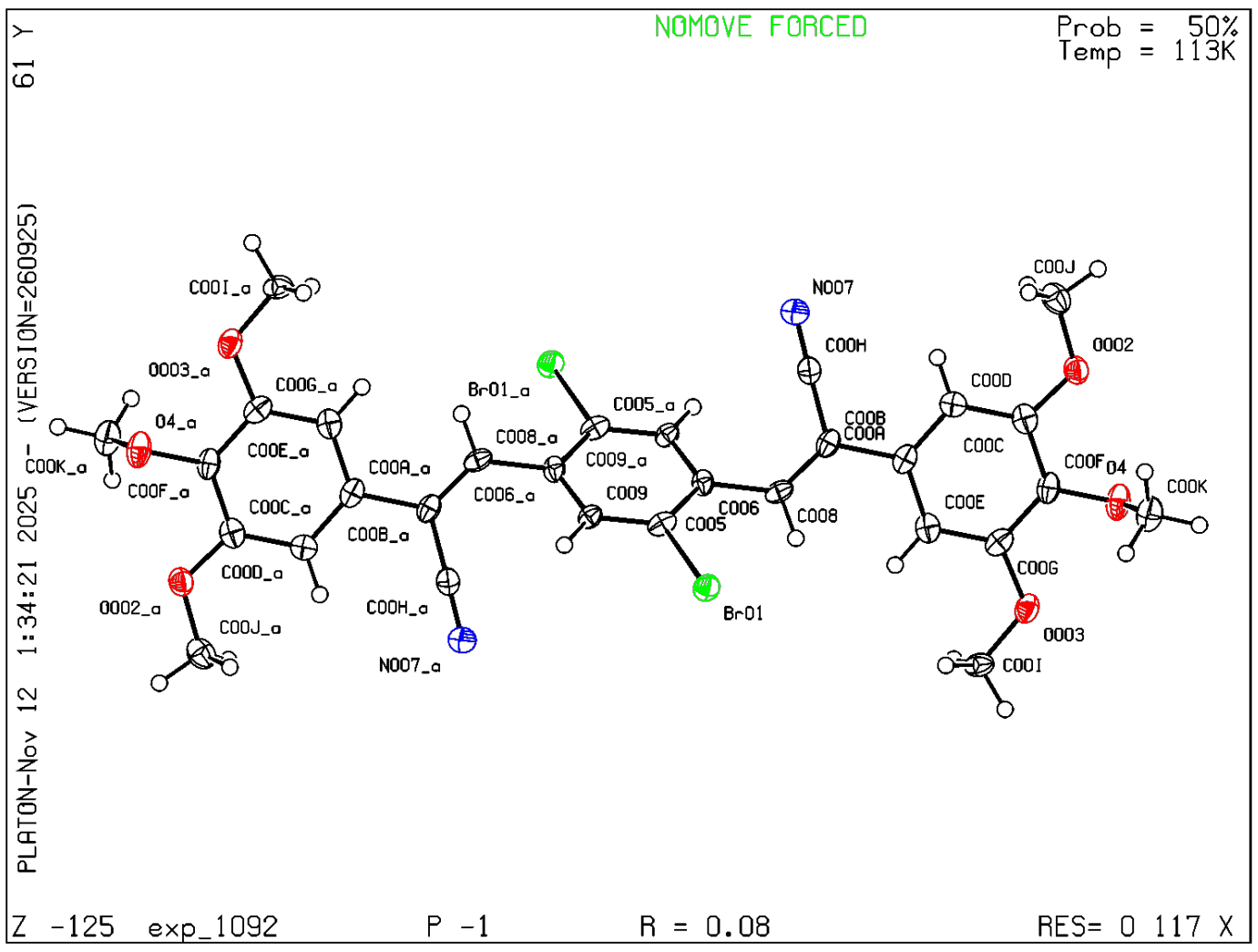

Download CIF editor (publCIF) from the IUCr
Download CIF editor (enCIFer) from the CCDC
Test a new CIF entry

Figure S36. CheckCIF report for crystal **α1** (IUCr: International Union of Crystallography).

Alert level B

PLAT431_ALERT_2_B Short Inter HL..A Contact Br01 ..O002 . 3.05 Ang.
1+x,3/2-y,1/2+z = 4_676 Check

Alert level C

PLAT094_ALERT_2_C Ratio of Maximum / Minimum Residual Density .... 2.33 Report

Alert level G

PLAT012_ALERT_1_G No _shelx_res_checksum Found in CIF ...... Please Check
PLAT720_ALERT_4_G Number of Unusual/Non-Standard Labels .... 33 Note
Br01 O002 O003 O004 N005 C006 H006 C007
C008 C009 C00A C00B C00C H00C C00D C00E
H00E C00F C00G H00G C00H C00I H00A H00B
H00D C00J H00F H00H H00I C00K H00J H00K
H00L
PLAT941_ALERT_3_G Average HKL Measurement Multiplicity .... 3.8 Low

0 **ALERT level A** = Most likely a serious problem - resolve or explain
1 **ALERT level B** = A potentially serious problem, consider carefully
1 **ALERT level C** = Check. Ensure it is not caused by an omission or oversight
3 **ALERT level G** = General information/check it is not something unexpected

1 ALERT type 1 CIF construction/syntax error, inconsistent or missing data
2 ALERT type 2 Indicator that the structure model may be wrong or deficient
1 ALERT type 3 Indicator that the structure quality may be low
1 ALERT type 4 Improvement, methodology, query or suggestion
0 ALERT type 5 Informative message, check

It is advisable to attempt to resolve as many as possible of the alerts in all categories. Often the minor alerts point to easily fixed oversights, errors and omissions in your CIF or refinement strategy, so attention to these fine details can be worthwhile. It is up to the individual to critically assess their own results and, if necessary, seek expert advice.

PLATON version of 26/09/2025; check.def file version of 20/09/2025

duplicate check

No duplication found

Datablock exp_1116 - ellipsoid plot

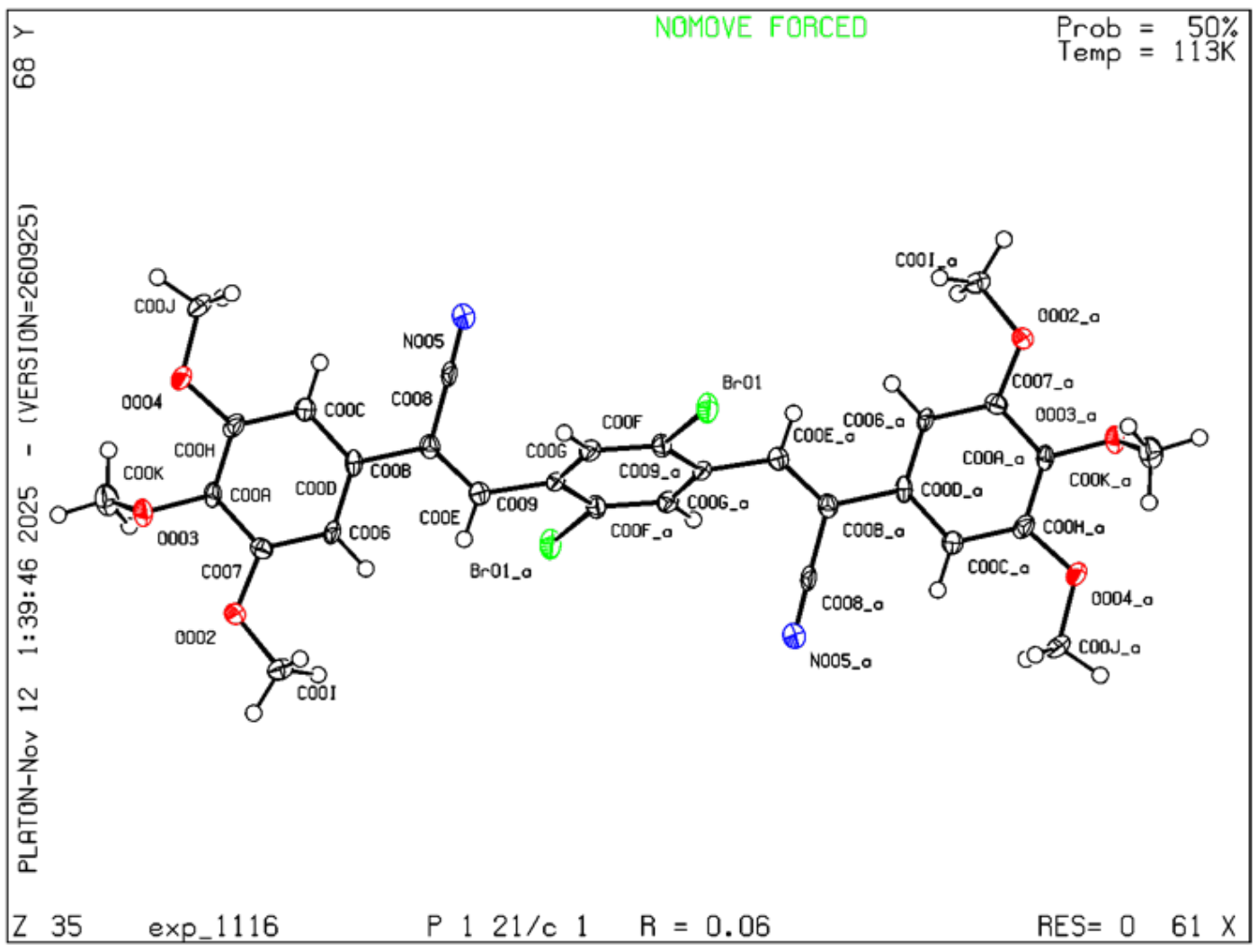

Download CIF editor (publCIF) from the IUCr
Download CIF editor (enCIFer) from the CCDC
Test a new CIF entry

Figure S37. CheckCIF report for crystal **β1** (IUCr).

PROBLEM: Short Inter HL..A Contact Br01..O002. 3.05 Ang. (PLAT431_2_B)

RESPONSE: The short intermolecular Br01···O002 contact (3.05 Å) corresponds to a directional halogen–oxygen interaction arising from the specific molecular packing in the crystal. This contact distance is within the reported range for Br···O halogen bonding and related attractive Br···O interactions, and no unusual displacement parameters or residual electron density are observed around Br01 or O002. Therefore, this contact is considered chemically reasonable and does not indicate a crystallographic error.

Alert level B

PLAT431_ALERT_2_B Short Inter HL..A Contact Br01 ..O002 . 3.05 Ang.
1+x,3/2-y,1/2+z = 4_676 Check

Alert level G

PLAT012_ALERT_1_G No _shelx_res_checksum Found in CIF ...... Please Check
PLAT720_ALERT_4_G Number of Unusual/Non-Standard Labels .... 33 Note
Br01 O002 O003 O004 N005 C006 H006 C007
C008 C009 H009 C00A H00A C00B C00C C00D
H00D C00E C00F C00G C00H C00I H00B H00C
H00E C00J H00F H00G H00H C00K H00I H00J
H00K
PLAT941_ALERT_3_G Average HKL Measurement Multiplicity .... 3.2 Low

0 **ALERT level A** = Most likely a serious problem - resolve or explain
1 **ALERT level B** = A potentially serious problem, consider carefully
0 **ALERT level C** = Check. Ensure it is not caused by an omission or oversight
3 **ALERT level G** = General information/check it is not something unexpected

1 ALERT type 1 CIF construction/syntax error, inconsistent or missing data
1 ALERT type 2 Indicator that the structure model may be wrong or deficient
1 ALERT type 3 Indicator that the structure quality may be low
1 ALERT type 4 Improvement, methodology, query or suggestion
0 ALERT type 5 Informative message, check

It is advisable to attempt to resolve as many as possible of the alerts in all categories. Often the minor alerts point to easily fixed oversights, errors and omissions in your CIF or refinement strategy, so attention to these fine details can be worthwhile. It is up to the individual to critically assess their own results and, if necessary, seek expert advice.

PLATON version of 26/09/2025; check.def file version of 20/09/2025

duplicate check

No duplication found

Datablock exp_1079 - ellipsoid plot

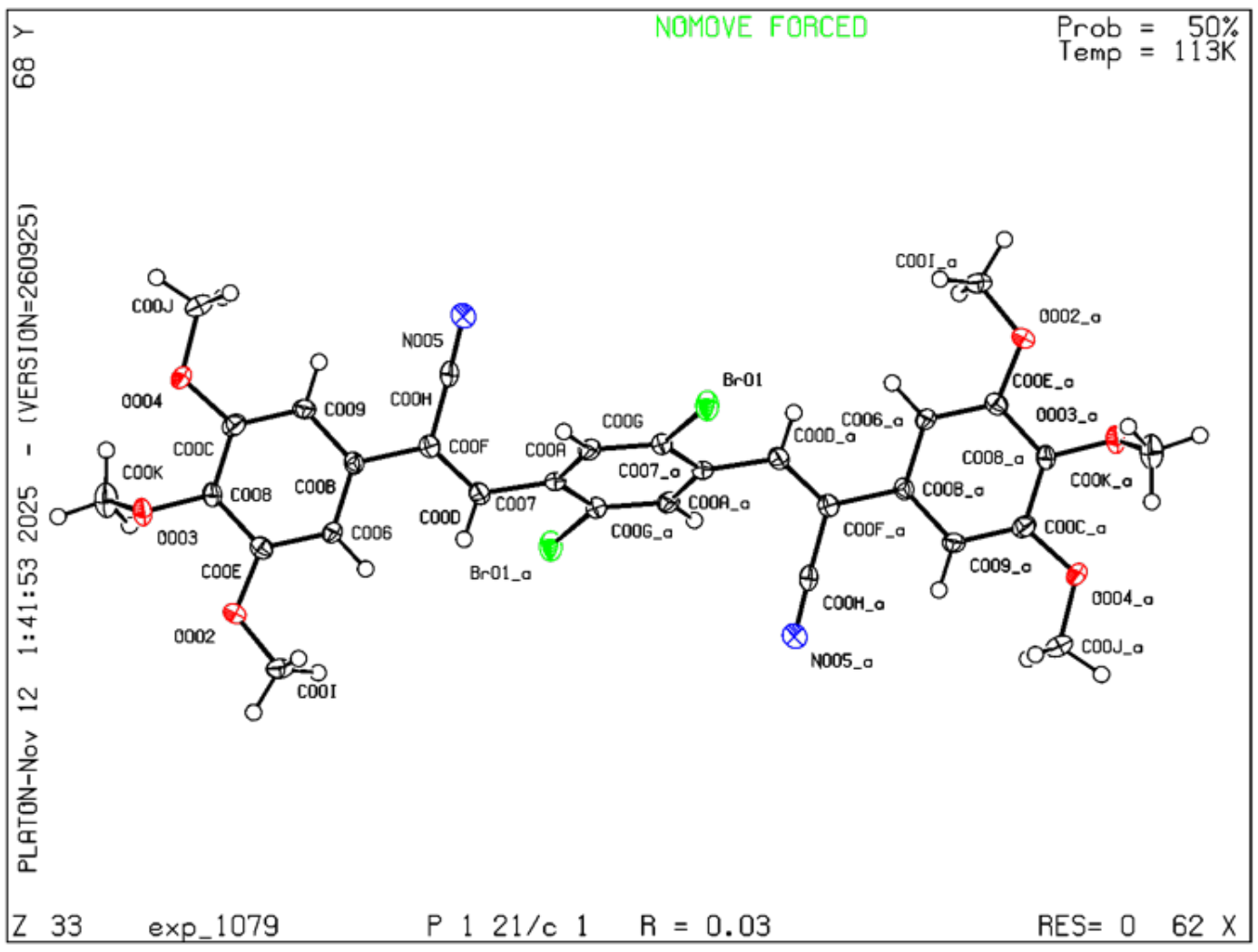

Download CIF editor (publCIF) from the IUCr
Download CIF editor (enCIFer) from the CCDC
Test a new CIF entry

Figure S38. CheckCIF report for crystal **α2** (IUCr).

PROBLEM: Short Inter HL..A Contact Br01..O002. 3.05 Ang. (PLAT431_2_B)

RESPONSE: The short intermolecular Br01···O002 contact (3.05 Å) corresponds to a directional halogen–oxygen interaction arising from the specific molecular packing in the crystal. This contact distance is within the reported range for Br···O halogen bonding and related attractive Br···O interactions, and no unusual displacement parameters or residual electron density are observed around Br01 or O002. Therefore, this contact is considered chemically reasonable and does not indicate a crystallographic error.

[1] Agilent: CrysAlis PRO. Agilent Technologies Ltd, Yarnton, Oxfordshire, England **2014**.

[2] O. V. Dolomanov, L. J. Bourhis, R. J. Gildea, J. A. K. Howard, H. Puschmann, *J. Appl. Crystallogr.* **2009**, *42*, 339–341.

[3] G. M. Sheldrick, *Acta Crystallogr., Sect. A: Found. Adv.* **2015**, *71*, 3–8.

[4] G. M. Sheldrick, *Acta Crystallogr., Sect. C: Struct. Chem.* **2015**, *71*, 3–8.

[5] C. F. Macrae, P. R. Edgington, P. McCabe, E. Pidcock, G. P. Shields, R. Taylor, M. Towler, J van de Streek *J. Appl. Cryst.* **2006**, *39*, 453–457.

[6] Gaussian 16, Revision B.01, M. J. Frisch, G. W. Trucks, H. B. Schlegel, G. E. Scuseria, M. A. Robb, J. R. Cheeseman, G. Scalmani, V. Barone, G. A. Petersson, H. Nakatsuji, X. Li, M. Caricato, A. V. Marenich, J. Bloino, B. G. Janesko, R. Gomperts, B. Mennucci, H. P. Hratchian, J. V. Ortiz, A. F. Izmaylov, J. L. Sonnenberg, D. Williams-Young, F. Ding, F. Lipparini, F. Egidi, J. Goings, B. Peng, A. Petrone, T. Henderson, D. Ranasinghe, V. G. Zakrzewski, J. Gao, N. Rega, G. Zheng, W. Liang, M. Hada, M. Ehara, K. Toyota, R. Fukuda, J. Hasegawa, M. Ishida, T. Nakajima, Y. Honda, O. Kitao, H. Nakai, T. Vreven, K. Throssell, J. A. Montgomery, Jr., J. E. Peralta, F. Ogliaro, M. J. Bearpark, J. J. Heyd, E. N. Brothers, K. N. Kudin, V. N. Staroverov, T. A. Keith, R. Kobayashi, J. Normand, K. Raghavachari, A. P. Rendell, J. C. Burant, S. S. Iyengar, J. Tomasi, M. Cossi, J. M. Millam, M. Klene, C. Adamo, R. Cammi, J. W. Ochterski, R. L. Martin, K. Morokuma, O. Farkas, J. B. Foresman, and D. J. Fox, Gaussian, Inc., Wallingford CT, **2016**.

[7] GaussView, Version 6.0, Roy Dennington, Todd A. Keith, and John M. Millam, Semichem Inc., Shawnee Mission, KS, **2016**.

[8] P. R. Spackman, M. J. Turner, J. J. McKinnon, S. K. Wolff, D. J. Grimwood, D. Jayatilaka, M. A. Spackman, *J. Appl. Cryst.* **2021**, *54*, 1006–1011.
